# Design and Synthesis of Novel Chalcogen-1,2,4-triazoles as Lead-like Glutathione Peroxidase-Mimetic Antioxidants

**DOI:** 10.1021/acsomega.6c00392

**Published:** 2026-06-25

**Authors:** Nathália L. B. Santos, Luana S. Gomes, Nathalia B. Sá, Pâmella Cordeiro, Aldo S. de Oliveira, Vanessa Nascimento

**Affiliations:** † SupraSelen Laboratory, Department of Organic Chemistry, Institute of Chemistry, 28110 Universidade Federal Fluminense, Campus of Valonguinho, 24020-141 Niterói, Rio de Janeiro, Brazil; ‡ LabSelen, Department of Chemistry, Universidade Federal de Santa Catarina, 88040-900 Florianópolis, Santa Catarina, Brazil; § Instituto Gulbenkian Institute de Medicina Molecular (GIMM), Faculdade de Medicina, Universidade de Lisboa, 1649-028 Lisboa, Portugal

## Abstract

Reactive oxygen species (ROS) play essential roles in cellular signaling; however, their dysregulation leads to oxidative stress associated with several chronic diseases. Organoselenium compounds, particularly selenocyanates, exhibit glutathione peroxidase (GPx)-mimetic activity, while the 1,2,4-triazole scaffold is a privileged pharmacophore in medicinal chemistry. In this work, a series of 15 new selenium- and sulfur-cyanides containing 1,2,4-triazole hybrids was designed, synthesized, and evaluated as potential GPx-mimetic antioxidants. The compounds were prepared via a three-step synthetic route and characterized by NMR spectroscopy and high-resolution mass spectrometry. GPx-like activity was assessed using a UV-based assay and supported by density functional theory (DFT) calculations and *in silico* ADME/Tox analyses. The synthetic approach afforded the target compounds in moderate to excellent yields (37–91%), with several selenium-containing derivatives displaying GPx-like activities up to 3.2-fold higher than the standard. Computational studies revealed correlations between enhanced activity, favorable electronic properties, and drug-like profiles, highlighting chalcogen–triazole hybrids as promising enzyme-mimetic antioxidants.

## Graphical abstract

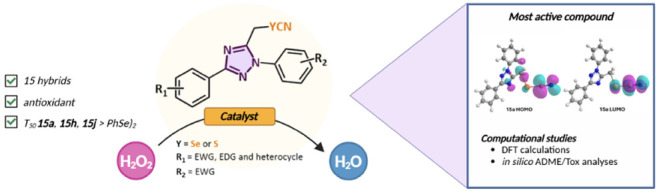

## 1 Introduction

Reactive oxygen species (ROS) are continuously generated in aerobic organisms as byproducts of metabolic and environmental processes. Under normal physiological conditions, ROS participate in signaling pathways and immune defense; however, excessive production or insufficient antioxidant defenses lead to oxidative stress, which contributes to several types of diseases such as cancer, neurodegenerative, cardiovascular, and inflammatory diseases among others.^1^ Consequently, the design of novel synthetic antioxidants with tunable redox properties has become one of the main goals in medicinal chemistry, aiming to restore cellular redox balance without causing cytotoxicity.^2,3^

Among emerging antioxidant scaffolds, organoselenium compounds have attracted particular attention because of their ability to mimic the enzymatic activity of glutathione peroxidase (GPx), thereby catalyzing the reduction of peroxides and protecting biomolecules from oxidative damage. In addition, other selenium-containing enzymes play essential roles in antioxidant defense and physiological regulation. For instance, thioredoxin reductase (TrxR) contributes to cellular redox homeostasis, iodothyronine deiodinases regulate thyroid hormone metabolism, and selenoprotein P functions as a selenium transporter with antioxidant properties. Notably, ebselen **1** and ethaselen **2** stand out as benchmark organoselenium potential drugs, both exhibiting potent GPx-like antioxidant properties. Moreover, ebselen has demonstrated broad pharmacological activity, including anti-inflammatory and neuroprotective effects. Likewise, ethaselen (BBSKE) has progressed to clinical trials for cancer therapy because of its ability to selectively induce oxidative stress in tumor cells. Similarly, in a recent study, the class of compounds **3** demonstrated antioxidant performance and showed effects on cerebral MAO-B activity, highlighting their potential as neuroprotective agents (Figure 1A).^4,5^

**Figure 1:**
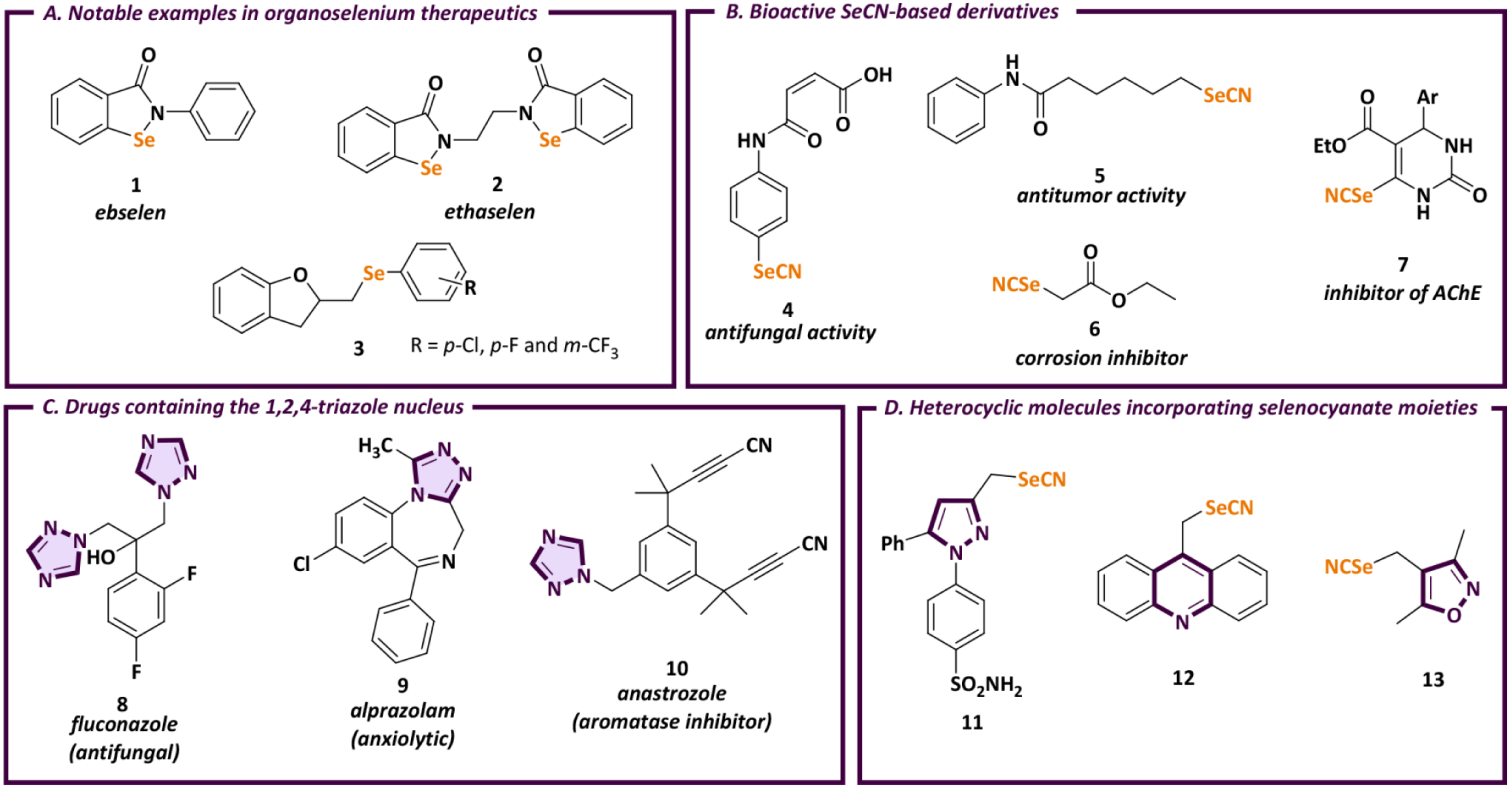
Overview of molecular scaffolds: (A) well-known organoselenium; (B) biologically active compounds bearing selenocyanate (SeCN) groups; (C) clinically used drugs featuring the 1,2,4-triazole nucleus; and (D) heterocyclic systems functionalized with selenocyanate moieties, highlighting the diversity of bioactive heteroatom-containing frameworks.

Along with that, it is reasonable to say that the incorporation of selenium into organic frameworks enhances redox flexibility and biological reactivity while maintaining low stoichiometric requirements for radical scavenging. In particular, selenocyanates (RSeCN) represent a versatile subclass of organoselenium compounds, exhibiting notable antioxidant activity and structural adaptability, which makes them attractive for biological applications, such as cytoprotection and redox modulation, and for the development of functional materials with oxidative stability, serving both as electrophilic selenium donors and precursors to selenols and diselenides with diverse biological profiles (Figure 1B).^6–8^ Several RSeCN derivatives have demonstrated cytoprotective and antioxidant behavior *in vitro*, although systematic investigations into structure–activity relationships, selectivity, and long-term stability remain limited.^9,10^

Parallel to this, the 1,2,4-triazole nucleus is recognized as a privileged heterocycle in drug design, showing greater representation in clinically established drugs when compared with other similar heterocycles. This core is present in widely used drugs such as fluconazole **8**, itraconazole **9**, and alprazolam **10** (Figure 1C). However, the synthetic development of 1,2,4-triazole derivatives remains comparatively underexplored in the literature.^11^ From a structural point of view, this nucleus combines aromatic stability, moderate hydrophobicity, and the ability to act as a hydrogen bond donor and acceptor, in addition to potential metal coordination sites,^12^ characteristics that support the multifunctional profile of biological activities reported for its derivatives, including antifungal, antibacterial, anti-inflammatory, anticancer, and antioxidant actions.^13^

Although heterocycles bearing selenocyanate groups have been described in the literature (Figure 1D), their integration with the 1,2,4-triazole nucleus remains largely unexplored.^14^ In view of the extensive interest in organoselenium chemistry and the well-established pharmacological relevance of 1,2,4-triazoles, this absence of studies represents a significant gap in both the structural diversification and biological exploration of this class of compounds. Guided by these considerations, the molecular hybridization of selenium-containing fragments with 1,2,4-triazole scaffolds represents a rational design strategy to merge two pharmacophores known for their redox activity into a single molecular entity. Such structural integration is expected to generate synergistic effects, and the electronic complementarity between the triazole ring and selenium moieties may promote efficient electron transfer processes, further supporting their potential as tunable redox modulators. Furthermore, considering the well-established capacity of organoselenium compounds to mimic glutathione peroxidase (GPx) and modulate thioredoxin reductase (TrxR) activity, these hybrid systems may also exhibit an enzyme-like catalytic behavior. Such properties could enable controlled peroxide reduction and support cellular redox homeostasis through biomimetic catalytic pathways.^9,15^

Therefore, the present study aimed at the synthesis and preliminary biological evaluation of new hybrid molecules composed of a 1,2,4-triazole core functionalized with a selenocyanate group (Scheme 1). The proposal aims to expand the library of available 1,2,4-triazole derivatives and investigate how the incorporation of selenium modulates the redox behavior and physicochemical profile of these structures, providing insights into their potential as GPx-mimetic and redox-active antioxidant candidates for medicinal chemistry exploration. To complement the experimental findings and enhance their translational relevance, a set of targeted *in silico* analyses was employed. Density functional theory (DFT) calculations were used to investigate frontier molecular orbital energetics and spatial distributions, enabling rationalization of electronic reactivity and redox participation of the chalcogen-containing moieties. In parallel, *in silico* ADMET profiling provided an early assessment of drug-likeness, pharmacokinetic behavior, and developability, allowing the integration of electronic, catalytic, and physicochemical descriptors into a coherent structure–activity framework.

**Scheme 1:**
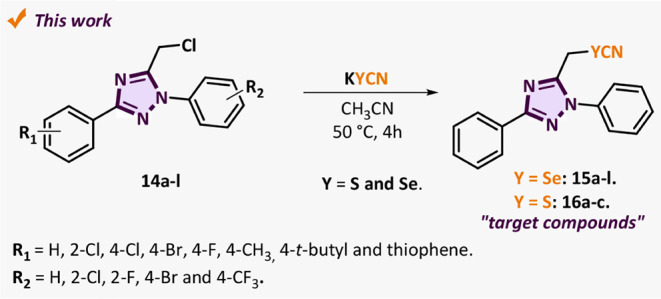
General Synthetic Route for 1,2,4-Triazole-Based Hybrids Bearing Selenocyanate and Thiocyanate Moieties

## 2 Materials and Methods

### 2.1 Chemistry

All solvents and reagents used in the synthesis, purification, and characterization were obtained from commercial sources, Sigma-Aldrich, Merck (Darmstadt, Germany), and Synth (São Paulo, Brazil), and were used without prior purification. For the isolation and purification of the compounds using column chromatography, a glass column was used, with silica gel as the stationary phase (0.063–0.2 mesh, Merck, Darmstadt, Germany), and a suitable solvent or solvent mixture was applied as the eluent. The fractions and compounds obtained were analyzed by thin-layer chromatography (TLC), using aluminum plates coated with silica gel 60 GF_254_ provided by Merck (Darmstadt, Germany), 0.25 mm thick, and with particles between 5 and 40 μm in diameter. The substances separated on the chromatographic plates were visualized using several development methods: in an iodine chamber, under ultraviolet light, or with a vanillin reagent followed by heating. Melting points were obtained on a Fisatom 430D apparatus and were uncorrected. APPI-Q-TOFMS measurements were taken on a mass spectrometer equipped with an automatic syringe pump for sample injection. The ^13^C and ^1^H NMR spectra were obtained on a Bruker Advance NEO spectrometer, operating at 500 MHz, using a direct broadband probe at 125 MHz, with chloroform-*d* as the solvent. Chemical shifts (δ) are reported in parts per million (ppm) relative to tetramethylsilane (TMS) as the internal standard. Multiplicities are indicated as s (singlet), d (doublet), t (triplet), dd (doublet of doublets), td (triplet of doublets), and m (multiple). Shielding constants (*J*) are reported in hertz (Hz). Assignments of individual ^13^C and ^1^H resonances are corroborated by 2D correlation experiments (COSY, HSQC, or HMBC) when necessary.

### 2.2 Synthesis Experimental Procedure

#### 2.2.1 General Procedure for the Synthesis of Hydrazones (**19a**–**l**).^16^

In a round-bottom flask, 0.5 mmol of benzaldehyde derivative (**17a**–**h**), 0.5 mmol of phenylhydrazine derivative (**18a**–**e**), and 5 mL of dichloromethane were mixed and stirred at room temperature for 30 min. After the reaction was complete, 50 mL of hexane was added to the bottom of the flask. The resulting mixture was filtered to obtain the desired product.

Most intermediate products were characterized by ^1^H and ^13^C NMR, showing spectroscopic data consistent with those reported in the literature. Derivatives **19g** and **19l** were used in the subsequent step without prior characterization.

##### 2.2.1.1 (*E*)-1-Benzylidene-2-phenylhydrazine (**19a**).^17^

0.0913 g, yield: 93%, yellow solid, mp: 155–158 °C (lit. 155–157 °C), ^1^H NMR (CDCl_3_, 500 MHz) δ = 7.63 (dd, *J* = 8.7 and 1.2 Hz; 2H), 7.60 (s, 1H), 7.34 (t, *J* = 7.5 Hz; 2H), 7.30–7.25 (m, 3H), 7.09 (dd, *J* = 8.7 and 1.2 Hz; 2H), 6.86 (t, *J* = 7.5 Hz; 1H). ^13^C NMR (CDCl_3_, 125 MHz) δ = 144.70, 137.35, 135.34, 129.35, 128.63, 128.44, 126.22, 120.13, 112.81.

##### 2.2.1.2 (*E*)-1-(4-Chlorobenzylidene)-2-phenylhydrazine (**19b**).^18^

0.0980 g, yield: 85%, white solid, mp: 126–128 °C (lit. 126–128 °C), ^1^H NMR (CDCl_3_, 500 MHz) δ = 7.59 (s, 1H), 7.56 (d, *J* = 8.6 Hz; 2H), 7.32 (d, *J* = 8.6 Hz; 2H), 7.29–7.25 (m, 2H), 7.09 (dd, *J* = 8.7 and 1.2 Hz; 2H), 6.88 (t, *J* = 7.3 Hz; 1H). ^13^C NMR (CDCl_3_, 125 MHz) δ = 144.42, 135.83, 133.98, 133.89, 129.37, 128.85, 127.28, 120.38, 112.81.

##### 2.2.1.3 (*E*)-1-(4-Bromobenzylidene)-2-phenylhydrazine (**19c**).^18^

0.1238 g, yield: 90%, brownish solid, mp: 121–124 °C (lit. 120–122 °C), ^1^H NMR (CDCl_3_, 500 MHz) δ = 7.56 (s, 1H), 7.50–7.46 (m, 4H), 7.27 (t, *J* = 8.0 Hz; 2H), 7.09 (d, *J* = 8.6 Hz; 2H), 6.88 (t, *J* = 6.8 Hz; 1H). ^13^C NMR (CDCl_3_, 125 MHz) δ = 144.38, 135.86, 134.33, 131.78, 129.38, 127.57, 122.19, 120.41, 112.83.

##### 2.2.1.4 (*E*)-1-(4-Fluorobenzylidene)-2-phenylhydrazine (**19d**).^18^

0.0932 g, yield: 87%, brownish solid, mp: 139–142 °C (lit. 145–147 °C), ^1^H NMR (CDCl_3_, 500 MHz) δ = 7.63–7.60 (m, 3H), 7.27 (t, *J* = 8.7 Hz; 2H), 7.09 (dd, *J* = 8.8 and 1.1 Hz; 2H), 7.05 (t, *J* = 8.8 Hz; 2H), 6.87 (t, *J* = 7.4 Hz; 1H). ^13^C NMR (CDCl_3_, 125 MHz) δ = 162.86 (d, *J* = 249.1 Hz), 144.61, 136.11, 131.54 (d, *J* = 3.7 Hz), 129.32, 127.75 (d, *J* = 8.3 Hz), 120.16, 115.65 (d, *J* = 22.1 Hz), 112.74.

##### 2.2.1.5 (*E*)-1-(3-(Thiophen-2-ylmethylene))-2-phenylhydrazine (**19e**).^19^

0.0841 g, yield: 83%, yellow solid, mp: 138–141 °C (lit. 139–141 °C), ^1^H NMR (CDCl_3_, 500 MHz) δ = 7.62–7.59 (m, 3H), 7.26 (t, *J* = 8.5 Hz; 2H), 7.09 (dd, *J* = 8.5 and 1.0 Hz; 2H), 7.04 (t, *J* = 9.0 Hz, 2H), 6,87 (t, J = 7.5 Hz; 1H). ^13^C NMR (CDCl_3_, 125 MHz) δ = 144.61, 136.11, 129.32, 127.79, 127.72, 120.17, 115.74, 115.57, 112.74.

##### 2.2.1.6 (*E*)-1-(2-Chlorobenzylidene)-2-phenylhydrazine (**19f**).^20^

0.0738 g, yield: 64%, yellow solid, mp: 86–88 °C (lit. 82–84 °C), ^1^H NMR (CDCl_3_, 500 MHz) δ = 8.10 (s, 1H), 8.09 (dd, *J* = 8.0 and 2.0 Hz; 1H), 7.35 (dd, *J* = 8.0 and 1.5 Hz; 1H), 7.30 (m, 3H), 7.22 (td, *J* = 7.6, 7.3, and 1.8 Hz; 1H), 7.14 (d, *J* = 7.0 Hz; 2H), 6.91 (t, *J* = 7.3 Hz; 1H). ^13^C NMR (CDCl_3_, 125 MHz) δ = 144.31, 133.50, 132.69, 132.54, 129.68, 129.33, 129.12, 126.89, 126.59, 120.48, 112.86.

##### 2.2.1.7 (*E*)-1-(4-Methylbenzylidene)-2-phenylhydrazine (**19h**).^21^

0.0746 g, yield: 71%, brownish solid, mp: 109–112 °C (lit. 110–112 °C), ^1^H NMR (CDCl_3_, 500 MHz) δ = 7.64 (s, 1H), 7.54 (d, *J* = 8.3 Hz; 2H), 7.26 (m, 2H), 7.17 (d, *J* = 7.9 Hz; 2H), 7.10 (dd, *J* = 8.6 and 1.1 Hz; 1H), 6.9 (t, *J* = 7.3 Hz; 1H), 2.36 (s, 3H). ^13^C NMR (CDCl_3_, 125 MHz) δ = 144.82, 138.48, 137.56, 132.56, 130.22, 129.33, 126.15, 119.93, 112.71, 21.38.

##### 2.2.1.8 (*E*)-1-Benzylidene-2-(2-chlorophenyl)­hydrazine (**19i**).^22^

0.0531 g, yield: 46%, pink solid, mp: 72–75 °C (lit. 73 °C), ^1^H NMR (CDCl_3_, 500 MHz) δ = ^1^H NMR (CDCl_3_, 500 MHz) δ = 7.84 (s), 7.70 (dd, *J* = 8.4 and 1.4 Hz; 2H), 7.65 (dd, *J* = 8.2 and 1.5 Hz; 1H), 7.40 (t, *J* = 7.3 Hz; 2H), 7.34 (t, *J* = 7.3 Hz; 1H), 7.29 (dd, *J* = 8.0 and 1.5 Hz; 1H), 7.26 (m, 1H), 6.81 (td, *J* = 7.7, 7.6, and 1.6 Hz; 1H). ^13^C NMR (CDCl_3_, 125 MHz) δ = 140.58, 139.54, 134.95, 130.20, 129.10, 128.87, 128.67, 127.96, 126.43, 120.02, 114.23.

##### 2.2.1.9 (*E*)-1-Benzylidene-2-(4-bromophenyl)­hydrazine (**19j**).^23^

0.0949 g, yield: 69%, brownish solid, mp: 119–122 °C (lit. 125 °C), ^1^H NMR (CDCl_3_, 500 MHz) δ = 7.28–7.37 (m, 5H), 7.65 (s, 1H), 7.63 (d, *J* = 7.7 Hz, 2H), 6.98 (d, *J* = 8.3 Hz, 2H). ^13^C NMR (CDCl_3_, 125 MHz) δ = 143.75, 138.11, 135.02, 132.09, 128.74, 128.69, 126.32, 114.40, 111.85.

##### 2.2.1.10 (*E*)-1-Benzylidene-2-(2-fluorophenyl)­hydrazine (**19k**)

0.0621 g, yield: 58%, brownish solid, mp: 101–103 °C, ^1^H NMR (CDCl_3_, 500 MHz) δ = 7.76 (s, 1H), 7.66 (d, *J* = 7.0 Hz, 2H), 7.61 (td, *J* = 8.3 and 1.8 Hz; 1H), 7.38 (t, *J* = 7.1 Hz; 2H), 7.31 (t, *J* = 7.4 Hz; 1H), 7.10 (t, *J* = 7.8 Hz; 1H), 7.02–7.01 (m, 1H). ^13^C NMR (CDCl_3_, 125 MHz) δ = 149.65 (d, *J* = 240 Hz), 139.29, 135.02, 133.12 (d, *J* = 9.2 Hz), 128.80, 128.67, 126.38, 124.90 (d, *J* = 3.7 Hz), 119.43 (d, *J* = 7.3 Hz), 114.80 (d, *J* = 17.5 Hz), 114.60 (d, *J* = 2.8 Hz).

#### 2.2.2 General Procedure for the Synthesis of 1,2,4-Triazoles (**14a**–**l**).^16^

A mixture of phenylhydrazone (0.15 mmol), CO­(NO_3_)_2_·6H_2_O (0.6 equiv) in 1,2-dichloroethane (3.0 mL) was stirred in a sealed tube at 110 °C for 3 h. After the reaction was complete, the mixture was cooled to room temperature, ethyl acetate was added, and the organic phase was washed with brine (3 × 100 mL). The organic phase was dried with anhydrous sodium sulfate and filtered, and the solvent was evaporated under reduced pressure. The product was purified by silica gel column chromatography using a hexane/ethyl acetate gradient mixture as eluent.

All compounds were characterized by ^1^H and ^13^C NMR, in agreement with the proposed structures and literature data. Derivatives **14h** and **14l** were confirmed by reaction monitoring and were used directly in the next step.

##### 2.2.2.1 5-(Chloromethyl)-1,3-diphenyl-1*H*-1,2,4-triazole (**14a**)

0.0181 g, yield: 45%, orange solid, mp: 125–127 °C (lit. 128–129 °C), ^1^H NMR (CDCl_3_, 500 MHz) δ = 8.17 (dd, *J* = 8.2 and 1.8 Hz; 2H), 7.67 (dd, *J* = 7.1 and 1.6 Hz; 2H), 7.60–7.52 (m, 3H), 7.48–7.43 (m, 3H), 4.71 (s, 2H). ^13^C NMR (CDCl_3_, 125 MHz) δ = 162.20, 151.71, 136.86, 130.22, 129.72, 129.66, 129.54, 128.63, 126.54, 124.69, 34.41.

##### 2.2.2.2 5-(Chloromethyl)-3-(4-chlorophenyl)-1-phenyl-1*H*-1,2,4-triazole (**14b**)

0.0159 g, yield: 35%, yellow liquid, ^1^H NMR (CDCl_3_, 500 MHz) δ = 8.04 (dd, *J* = 9.0 and 2.0 Hz; 2H), 7.58 (dd, *J* = 6.9 and 1.5 Hz; 2H), 7.53–7.48 (m, 3H), 7.36 (dd, *J* = 9.0 and 2.0 Hz; 2H), 4.62 (s, 2H). ^13^C NMR (CDCl_3_, 125 MHz) δ = 160.31, 150.87, 135.73, 134.62, 128.76, 128.65, 127.89, 127.77, 126.84, 123.66, 33.31.

##### 2.2.2.3 3-(4-Bromophenyl)-5-(chloromethyl)-1-phenyl-1*H*-1,2,4-triazole (**14c**)

0.0217 g, yield: 42%, yellow solid, mp: 118–120 °C (lit. 98–99 °C), ^1^H NMR (CDCl_3_, 500 MHz) δ = 8.03 (dd, *J* = 9.0 and 2.4 Hz; 2H), 7.63 (dd, *J* = 6.9 and 1.6 Hz; 2H), 7.59–7.51 (m, 5H), 4.68 (s, 2H). ^13^C NMR (CDCl_3_, 125 MHz) δ = 161.37, 151.91, 136.74, 131.86, 129.79, 129.69, 129.25, 128.11, 124.67, 123.97, 34.33.

##### 2.2.2.4 5-(Chloromethyl)-3-(4-fluorophenyl)-1-phenyl-1*H*-1,2,4-triazole (**14d**)

0.0172 g, yield: 40%, yellow solid, mp: 118–120 °C, ^1^H NMR (CDCl_3_, 500 MHz) δ = 8.12–8.09 (m, 2H), 7.61–7.59 (m, 2H), 7.55–7.47 (m, 3H), 7.11–7.08 (m, 2H), 4.65 (s, 2H). ^13^C NMR (CDCl_3_, 125 MHz) δ = 164.77 (d, *J* = 248.8 Hz), 162.78, 161.42, 151.78, 136.78, 129.74, 129.59, 128.52 (d, *J* = 7.5 Hz), 128.46, 126.53 (d, *J* = 2.5 Hz), 126.51, 124.65, 115.73 (d, *J* = 21.3 Hz), 115.56, 34.35. MS (ESI^+^, low resolution): *m*/*z* 239 ([M – Cl]^+^).

##### 2.2.2.5 5-(Chloromethyl)-1-phenyl-3-(Thiophen-2-yl)-1*H*-1,2,4-triazole (**14e**)

0.0156 g, yield: 38%, yellow solid, mp: 127–130 °C (lit. 128–128.5 °C), ^1^H NMR (CDCl_3_, 500 MHz) δ = 7.69 (dd, *J* = 5.0 and 1.2 Hz; 1H), 7.58 (dd, *J* = 7.0 and 1.7 Hz; 2H), 7.51–7.43 (m, 3H), 7.31 (dd, *J* = 5.0 Hz, 1.2 Hz; 1H), 7.06–7.04 (m, 1H), 4.60 (s, 2H). ^13^C NMR (CDCl_3_, 125 MHz) δ = 161.11, 150.79, 135.79, 132.67, 129.18, 129.10, 128.72, 128.58, 127.65, 127.46, 125.56, 123.70, 33.27.

##### 2.2.2.6 5-(Chloromethyl)-3-(2-chlorophenyl)-1-phenyl-1*H*-1,2,4-triazole (**14f**)

0.0113 g, yield: 25%, yellow liquid, ^1^H NMR (CDCl_3_, 500 MHz) δ = 7.90–7.87 (m, 1H), 7.62–7.60 (m, 2H), 7.52–7.49 (m, 2H), 7.47–7.43 (m, 2H), 7.31–7.27 (m, 2H), 4.67 (s, 2H). ^13^C NMR (CDCl_3_, 125 MHz) δ = 159.67, 150.17, 135.76, 131.90, 130.52, 129.72, 129.39, 128.72, 128.57, 128.36, 125.72, 123.63, 33.36.

##### 2.2.2.7 3-(4-(tert-Butyl)­phenyl)-5-(chloromethyl)-1-phenyl-1*H*-1,2,4-triazole (**14g**)

0.0170 g, yield: 35%, orange solid, mp: 103–105 °C (lit. 110–111 °C), ^1^H NMR (CDCl_3_, 500 MHz) δ = 8.01 (dd, *J* = 8.8 and 2.2 Hz; 2H), 7.58 (dd, *J* = 6.9 and 1.5 Hz; 2H), 7.52–7.46 (m, 3H), 7.40 (dd, *J* = 8.8 and 2.2 Hz; 2H), 4.63 (s, 2H), 1.29 (s, 9H). ^13^C NMR (CDCl_3_, 125 MHz) δ = 161.23, 151.86, 150.56, 135.91, 128.69, 128.46, 126.38, 125.27, 124.56, 123.69, 33.79, 33.42, 30.25.

##### 2.2.2.8 5-(Chloromethyl)-1-(2-chlorophenyl)-3-phenyl-1*H*-1,2,4-triazole (**14i**)

0.0136 g, yield: 30%, yellow liquid, ^1^H NMR (CDCl_3_, 500 MHz) δ = 8.09 (dd, *J* = 8.2 and 1.8 Hz; 2H), 7.55 (dd, *J* = 7.7 and 1.5 Hz; 1H), 7.52 (dd, *J* = 7.7 and 1.8 Hz; 1H), 7.48 (td, *J* = 7.7 and 1.8 Hz; 1H), 7.44 (dd, *J* = 7.7 and 1.5 Hz; 1H), 7.41–7.43 (m, 3H), 4.53 (s, 2H). ^13^C NMR (CDCl_3_, 125 MHz) δ = 162.46, 153.20, 134.33, 131.85, 131.83, 130.68, 130.03, 129.77, 129.63, 128.65, 128.03, 126.60, 34.01.

##### 2.2.2.9 1-(4-Bromophenyl)-5-(chloromethyl)-3-phenyl-1*H*-1,2,4-triazole (**14j**)

0.0171 g, yield: 33%, yellow solid, mp: 118–121 °C, ^1^H NMR (CDCl_3_, 500 MHz) δ = 8.07 (dd, *J* = 8.1 and 2.1 Hz; 2H), 7.63 (d, *J* = 8.5 Hz; 2H), 7.48 (d, *J* = 8.5 Hz; 2H), 7.40–7.36 (m, 3H), 4.62 (s, 2H). ^13^C NMR (CDCl_3_, 125 MHz) δ = 161.29, 150.65, 134.83, 131.91, 128.92, 128.81, 127.66, 125.53, 125.06, 122.47, 33.31. MS (ESI^+^, low resolution): *m*/*z* 369 ([M Na]^+^).

##### 2.2.2.10 5-(Chloromethyl)-1-(2-fluorophenyl)-3-phenyl-1*H*-1,2,4-triazole (**14k**)

0.0129 g, yield: 30%, yellow solid, mp: 90–92 °C (lit. 78–79 °C), ^1^H NMR (CDCl_3_, 500 MHz) δ = 8.08 (dd, *J* = 8.2 and 1.9 Hz; 2H), 7.54 (td, *J* = 7.6 and 1.7 Hz; 1H), 7.51–7.46 (m, 1H), 7.40–7.35 (m, 3H), 7.31–7.24 (m, 2H), 4.59 (s, 2H). ^13^C NMR (CDCl_3_, 125 MHz) δ = 161.68, 156.51 (d, *J* = 250 Hz), 154.50, 152.28, 131.00 (d, *J* = 7.5 Hz), 130.94, 129.06, 128.76, 127.73, 127.64, 125.56, 124.19 (d, *J* = 2.5 Hz), 124.16, 123.80 (d, *J* = 11.3 Hz), 123.71, 116.09 (d, *J* = 18.9 Hz), 115.94, 33.11.

#### 2.2.3 General Procedure for the Synthesis of Seleno and Sulfur-Cyanate Derivatives (**15a**–**l**, **16a**–**c**).^24^

In a round-bottom flask, 0.1 mmol of 1,2,4-triazole derivatives (**14a**–**l**), 0.11 mmol of potassium selenocyanate, and 1 mL of acetonitrile were mixed and heated to 50 °C for 4 h. Subsequently, after cooling the mixture to room temperature, ethyl acetate was added and the organic phase was washed with brine (3 × 100 mL). The organic phase was dried with anhydrous sodium sulfate and filtered, and the solvent was evaporated under reduced pressure. The product was purified by silica gel column chromatography using a hexane/ethyl acetate gradient mixture as eluent.

##### 2.2.3.1 1,3-Diphenyl-5-(selenocyanatomethyl)-1*H*-1,2,4-triazole (**15a**)

0.0309 g, yield: 91%, yellow solid, mp: 151–154 °C, ^1^H NMR (CDCl_3_, 500 MHz) δ = 8.13 (d, *J* = 7.3 Hz; 2H), 7.61–7.54 (m, 5H), 7.48–7.43 (m, 3H), 4.48 (s, 2H). ^13^C NMR (CDCl_3_, 125 MHz) δ = 162.09, 150.20, 136.49, 129.98, 129.87, 129.81, 128.67, 126.53, 124.85, 100.87, 21.04. HRMS (ESI-QTOF) calculated mass for C_16_H_12_N_4_Se [M + H]^+^: 341.0307, found: 341.0305.

##### 2.2.3.2 3-(4-Chlorophenyl)-1-phenyl-5-(selenocyanatomethyl)-1*H*-1,2,4-triazole (**15b**)

0.0299 g, yield: 80%, yellow solid, mp: 143–146 °C, ^1^H NMR (CDCl_3_, 500 MHz) δ = 7.99 (d, *J* = 8.6 Hz; 2H), 7.55–7.45 (m, 5H), 7.36 (d, *J* = 8.5 Hz; 2H), 4.31 (s, 2H). ^13^C NMR (CDCl_3_, 125 MHz) δ = 160.18, 149.43, 135.34, 134.77, 129.02, 128.99, 127.93, 127.46, 126.83, 123.84, 99.74, 19.87. HRMS (ESI-QTOF) calculated mass for C_16_H_11_ClN_4_Se [M + H]^+^: 374.9917, found: 374.9971.

##### 2.2.3.3 3-(4-Bromophenyl)-1-phenyl-5-(selenocyanatomethyl)-1*H*-1,2,4-triazole (**15c**)

0,0292g, yield: 70%, yellow solid, mp: 127–130 °C, ^1^H NMR (CDCl_3_, 500 MHz) δ = 8.00 (d, *J* = 8.5 Hz; 2H), 7.62–7.56 (m, 5H), 7.55–7.53 (m, 2H), 4.46 (s, 2H). ^13^C NMR (CDCl_3_, 125 MHz) δ = 161.25, 150.46, 136.36, 131.88, 130.02, 130.00, 128.93, 128.09, 124.85, 100.69, 20.88. HRMS (ESI-QTOF) calculated mass for C_16_H_11_BrN_4_Se [M + H]^+^: 418.9412, found: 418.9408.

##### 2.2.3.4 3-(4-Fluorophenyl)-1-phenyl-5-(selenocyanatomethyl)-1*H*-1,2,4-triazole (**15d**)

0.0268 g, yield: 75%, yellow solid, mp: 143–146 °C, ^1^H NMR (CDCl_3_, 500 MHz) δ = 8.13–8.10 (m, 2H), 7.62–7.54 (m, 5H), 7.13 (t, *J* = 8.6 Hz; 3H), 4.47 (s, 2H). ^13^C NMR (CDCl_3_, 125 MHz) δ = 164.84 (d, *J* = 247.5 Hz), 162.86, 161.31, 150.31, 136.41, 130.00, 129.93, 128.54 (d, *J* = 8.8 Hz), 128.47, 126.22 (d, *J* = 3.8 Hz), 126.19, 124.84, 115.80 (d, *J* = 21.3 Hz), 115.63, 100.73, 20.93. HRMS (ESI-QTOF) calculated mass for C_16_H_11_FN_4_Se [M + H]^+^: 359.0213, found: 359.0210.

##### 2.2.3.5 1-Phenyl-5-(selenocyanatomethyl)-3-(thiophen-2-yl)-1*H*-1,2,4-triazole (**15e**)

0.0242 g, yield: 70%, brownish solid, mp: 118–121 °C, ^1^H NMR (CDCl_3_, 500 MHz) δ = 7.66 (d, *J* = 3.4 Hz; 1H), 7.53–7.45 (m, 5H), 7.31 (d, *J* = 4.9 Hz; 1H), 7.04 (t, *J* = 4.2 Hz; 1H), 4.37 (s, 2H). ^13^C NMR (CDCl_3_, 125 MHz) δ = 158.36, 150.34, 136.22, 132.61, 130.01, 129.99, 127.83, 127.19, 127.04, 125.03, 100.66, 20.71. HRMS (ESI-QTOF) calculated mass for C_14_H_10_N_4_SSe [M + H]^+^: 346.9871, found: 346.9865.

##### 2.2.3.6 3-(2-Chlorophenyl)-1-phenyl-5-(selenocyanatomethyl)-1*H*-1,2,4-triazole (**15f**)

0.0257 g, yield: 54%, yellow solid, mp: 92–95 °C, ^1^H NMR (CDCl_3_, 500 MHz) δ = 7.94–7.92 (m, 1H), 7.62–7.54 (m, 5H), 7.52–7.50 (m, 1H), 7.38–7.34 (m, 2H), 4.51 (s, 2H). ^13^C NMR (CDCl_3_, 125 MHz) δ = 160.58, 149.72, 136.40, 132.94, 131.42, 130.81, 130.53, 129.98, 129.90, 129.01, 126.77, 124.78, 100.83, 20.98. HRMS (ESI-QTOF) calculated mass for C_16_H_11_ClN_4_Se [M + H]^+^: 374.9917, found: 374.9856.

##### 2.2.3.7 3-(4-Tert-butylphenyl)-1-phenyl-5-(selenocyanatomethyl)-1*H*-1,2,4-triazole (**15g**)

0.0231 g, yield: 56%, yellow solid, mp: 115–118 °C, ^1^H NMR (CDCl_3_, 500 MHz) δ = 7.97 (d, *J* = 8.5 Hz; 2H), 7.53–7.41 (m, 5H), 7.40 (d, *J* = 8.5 Hz; 2H), 4.41 (s, 2H), 1,28 (s, 9H). ^13^C NMR (CDCl_3_, 125 MHz) δ = 161.07, 152.08, 149.19, 135.48, 128.95, 128.82, 126.03, 125.29, 124.62, 123.90, 100.04, 33.81, 30.23, 20.03. HRMS (ESI-QTOF) calculated mass for C_20_H_20_N_4_Se [M + H]^+^: 397.0933, found: 397.0871.

##### 2.2.3.8 1-Phenyl-5-(selenocyanatomethyl)-3-(p-tolyl)-1*H*-1,2,4-triazole (**15h**)

0.0288 g, yield: 77%, yellow solid, mp: 138–141 °C, ^1^H NMR (CDCl_3_, 500 MHz) δ = 8.01 (d, *J* = 8.2 Hz; 2H), 7.61–7.53 (m, 5H), 7.26 (d, *J* = 8.2 Hz; 2H), 4.48 (s, 2H), 2.40 (s, 3H). ^13^C NMR (CDCl_3_, 125 MHz) δ = 162.19, 150.17, 139.94, 136.52, 130.00, 129.87, 129.43, 127.13, 126.49, 124.91, 101.05, 21.49, 21.07. HRMS (ESI-QTOF) calculated mass for C_17_H_14_N_4_Se [M + H]^+^: 355.0464, found: 355.0461.

##### 2.2.3.9 1-(2-Chlorophenyl)-3-phenyl-5-(selenocyanatomethyl)-1*H*-1,2,4-triazole (**15i**)

0.0176 g, yield: 48%, brownish solid, mp: 83–86 °C, ^1^H NMR (CDCl_3_, 500 MHz) δ = 8.05 (td, *J* = 8.2 and 2.0 Hz; 2H), 7.58–7.55 (m, 2H), 7.49 (td, *J* = 7.5 and 1.8 Hz; 1H), 7.45 (td, *J* = 7.5 and 1.8 Hz; 1H), 7.42–7.37 (m, 3H), 4.25 (s, 2H). ^13^C NMR (CDCl_3_, 125 MHz) δ = 161.64, 151.33, 132.87, 132.57, 131.12, 130.54, 129.81, 129.14, 128.93, 128.77, 127.71, 127.45, 127.39, 125.59, 99.75, 19.35. HRMS (ESI-QTOF) calculated mass for C_16_H_11_ClN_4_Se [M + H]^+^: 374.9917, found: 374.9973.

##### 2.2.3.10 1-(4-Bromophenyl)-3-phenyl-5-(selenocyanatomethyl)-1*H*-1,2,4-triazole (**15j**)

0,0296 g, yield: 72%, yellow solid, mp: 145–148 °C, ^1^H NMR (CDCl_3_, 500 MHz) δ = 8.04 (d, *J* = 8.0 Hz; 2H), 7.65 (d, *J* = 8.7 Hz; 2H), 7.41–7.35 (m, 5H), 4.40 (s, 2H). ^13^C NMR (CDCl_3_, 125 MHz) δ = 161.25, 149.43, 134.41, 132.17, 128.98, 128.64, 127.72, 125.55, 125.33, 122.90, 99.80, 19.79. HRMS (ESI-QTOF) calculated mass for C_16_H_11_BrN_4_Se [M + H]^+^: 418.9412, found: 418.9410.

##### 2.2.3.11 1-(2-Fluorophenyl)-3-phenyl-5-(selenocyanatomethyl)-1*H*-1,2,4-triazole (**15k**)

0.0201 g, yield: 54%, orange solid, mp: 115–118 °C, ^1^H NMR (CDCl_3_, 500 MHz) δ = 8.14–8.12 (m, 2H), 7.64 (td, *J* = 7.7 and 1.7 Hz; 1H), 7.59–7.57 (m, 1H), 7.48–7.44 (m, 3H), 7.42–7.38 (m, 1H), 7.36 (ddd, *J* = 9.8, 8.4, and 1.3 Hz; 1H), 4.39 (s, 2H). ^13^C NMR (CDCl_3_, 125 MHz) δ = 162.83, 157.03 (d, *J* = 248.8 Hz), 155.02, 152.08, 132.22 (d, *J* = 7.5 Hz), 132.16, 129.93, 129.76, 128.78, 128.70, 126.58, 125.59 (d, *J* = 3.8 Hz), 125.56, 124.35 (d, *J* = 11.3 Hz), 124.26, 117.26 (d, *J* = 20.0 Hz), 117.10, 100.73, 20.44. HRMS (ESI-QTOF) calculated mass for C_16_H_11_FN_4_Se [M + H]^+^: 359.0213, found: 359.0166.

##### 2.2.3.12 3-Phenyl-5-(selenocyanatomethyl)-1-(4-(trifluoromethyl)­phenyl)-1*H*-1,2,4-triazole (**15l**)

0.0079 g, yield: 37%, yellow solid, mp: 133–136 °C, ^1^H NMR (CDCl_3_, 500 MHz) δ = 8.14–8.11 (m, 2H), 7.88 (d, *J* = 8.3 Hz; 2H), 7.74 (d, *J* = 8.3 Hz; 2H), 7.49–7.45 (m, 3H), 4.53 (s, 2H). ^13^C NMR (CDCl_3_, 125 MHz) δ = 162.51, 150.44, 133.57, 130.14 (d, *J* = 3.8 Hz), 130.11, 129.51, 128.75 (q, *J* = 268.0 Hz), 128.47, 127.29 (q, *J* = 3.8 Hz), 127.26, 127.23, 127.20, 126.61, 124.90, 100.68, 20.93. HRMS (ESI-QTOF) calculated mass for C_17_H_11_F_3_N_4_Se [M + H]^+^: 409.0181, found: 409.0181.

##### 2.2.3.13 1,3-Diphenyl-5-(thiocyanatomethyl)-1*H*-1,2,4-triazole (**16a**)

0.0123 g, yield: 41%, yellow solid, mp: 104–107 °C, ^1^H NMR (CDCl_3_, 500 MHz) δ ppm: 8.16 (d, *J* = 6,1 Hz; 2H), 7.62–7.58 (m, 3H), 7.50–7.43 (m, 5H), 4.42 (s, 2H). ^13^C NMR (CDCl_3_, 125 MHz) δ = 162.40, 149.65, 136.34, 133.62, 130.07, 130.00, 129.84, 128.69, 128.47, 126.61, 125.30, 110.39, 28.23. HRMS (ESI-QTOF) calculated mass for C_16_H_12_N_4_S [M + H]^+^: 293.0863, found: 293.0848.

##### 2.2.3.14 1-Phenyl-5-(thiocyanatomethyl)-3-(p-tolyl)-1*H*-1,2,4-triazole (**16b**)

0.0162 g, yield: 74%, yellow solid, mp: 108–111 °C, ^1^H NMR (CDCl_3_, 500 MHz) δ = 7.96 (d, *J* = 8.3 Hz; 2H), 7.54–7.50 (m, 3H), 7.48–7.46 (m, 2H), 7.20 (d, *J* = 8.3 Hz; 2H), 4.34 (s, 2H), 2.34 (s, 3H). ^13^C NMR (CDCl_3_, 125 MHz) δ = 161.50, 148.44, 138.91, 135.37, 129.20, 128.98, 128.39, 128.19, 126.13, 125.58, 124.28, 109.45, 27.25, 20.45. HRMS (ESI-QTOF) calculated mass for C_17_H_14_N_4_S [M + H]^+^: 307.1019, found: 307.1002.

##### 2.2.3.15 3-(4-Chlorophenyl)-1-phenyl-5-(thiocyanatomethyl)-1*H*-1,2,4-triazole (**16c**)

0.0171 g, yield: 52%, yellow solid, mp: 121–124 °C, ^1^H NMR (CDCl_3_, 500 MHz) δ = 8.02 (d, *J* = 8.5 Hz; 2H), 7.55–7.50 (m, 3H), 7.47–7.45 (m, 2H), 7.36 (d, *J* = 8.5 Hz; 2H), 4.31 (s, 2H). ^13^C NMR (CDCl_3_, 125 MHz) δ = 160.51, 148.75, 135.23, 134.78, 129.15, 129.03, 127.93, 127.51, 126.87, 124.22, 109.29, 27.18. HRMS (ESI-QTOF) calculated mass for C_16_H_11_ClN_4_S [M + H]^+^: 327.0473, found: 327.0460.

### 2.3 Gpx-like Catalytic Activity Assay

#### 2.3.1 General Procedure for Evaluation of the Gpx-like Activity of Chalcogenides

The experiments to evaluate the catalytic activity of the selenides as glutathione peroxidase (GPx) enzyme mimetics were carried out according to the Tomoda method.^25^ In a quartz cuvette, the selenium catalyst (final concentration = 0.01 mM), thiophenol (final concentration = 5 mM), and MeOH were mixed at 25 (±3) °C. The measurements were performed using a Varian Cary 50 UV–vis spectrophotometer (wavelength range: 200–800 nm; resolution: 1 nm; scan rate: 600 nm/min). The spectrophotometer was programmed to record UV absorbance readings at a wavelength of 305 nm every 10 s, corresponding to the formation of PhSSPh. After approximately 120 s from the start of the experiment, the model GPx catalytic reaction (H_2_O_2_ + 2PhSH → 2H_2_O + PhSSPh) was initiated by the addition of H_2_O_2_ (final concentration = 10 mM). The reaction was monitored for an additional 100 s. Each experiment was performed in triplicate. GPx-like activities are reported as mean ± deviation from triplicate measurements (n = 3) and are intended for preliminary trend comparison rather than inferential statistical testing. The reaction was also monitored in the absence of a catalyst and using diphenyl diselenide as a catalytic reference. This assay provides preliminary evidence of GPx-like catalytic activity and should be interpreted as an initial screening method, rather than a definitive mechanistic or quantitative evaluation of peroxide reduction.

#### 2.3.2 Calculation of T_50_

The stoichiometric ratio between PhSSPh and PhSH was 1:2. The rate of PhSSPh formation was determined from the linear portion of the spectrophotometric curve, corresponding to the peroxidase-like catalytic activity. On the basis of the total concentration of PhSH and the rate of PhSSPh formation, the T_50_ value, defined as the time required for the consumption of 50% of the initial PhSH, was obtained by extrapolation.

### 2.4 *In Silico* Studies

#### 2.4.1 Selection of Compounds for *In Silico* Analysis

Considering the size of the compound library and the exploratory scope of the present study, a focused and representative subset of compounds was selected for in silico evaluation. This strategy was adopted to enable qualitative interpretation of pharmacokinetic and electronic trends while avoiding overparameterization or unjustified quantitative extrapolation across the full series. Compound selection was guided by (i) experimental glutathione peroxidase (GPx)-like catalytic activity, expressed as relative rate (Vrel), and (ii) structural diversity with respect to chalcogen identity (selenium versus sulfur) and aromatic substitution patterns. Based on these criteria, the selected subset comprised three highly active selenocyanate derivatives (**15a**, **15h,** and **15j**), a sulfur-containing analog displaying comparable catalytic performance (**16c**), and three compounds exhibiting significantly lower GPx-like activity (**15i**, **15l**, and **16a**). This selection enabled direct comparison between high- and low-activity profiles and supported qualitative structure–property and structure–activity analyses.

#### 2.4.2 *In Silico* Pharmacokinetic and Drug-Likeness Assessment

Pharmacokinetic and drug-likeness properties of the selected compounds were evaluated using the SwissADME web server and the ADMETlab 3.0 platform. SwissADME was used to calculate key physicochemical descriptors and medicinal chemistry parameters, including molecular weight, lipophilicity (consensus LogP), topological polar surface area (TPSA), hydrogen-bonding capacity, number of rotatable bonds, and compliance with widely accepted drug-likeness filters (Lipinski, Ghose, Veber, Egan, and Muegge). These descriptors were used to position the investigated compounds within drug-like chemical space and to support comparative analysis across the series. Complementary ADMET predictions were obtained using ADMETlab 3.0, including parameters related to absorption, distribution, metabolism, excretion, and toxicity. In addition, structural alerts associated with pan-assay interference compounds (PAINS), promiscuity, and toxicophore rules were evaluated. Notably, the most active derivatives did not present critical PAINS alerts, supporting their suitability as lead-like candidates. The bioavailability radar plots presented in this study were generated using ADMETlab 3.0 and used as qualitative tools to integrate multiple physicochemical descriptors relevant to oral drug-likeness, including molecular size, lipophilicity, polarity, solubility, flexibility, and saturation. All pharmacokinetic and drug-likeness predictions were interpreted qualitatively, with the aim of supporting experimental observations and guiding structure–property relationships, rather than providing definitive pharmacokinetic conclusions.

#### 2.4.3 Similarity-Based Target Class Profiling

Similarity-based target prediction was performed by using the SwissTargetPrediction platform. Predicted associations were analyzed at the level of target classes rather than individual molecular targets in order to maintain a hypothesis-generating and nonspeculative interpretation. This approach enabled comparison between high- and low-activity derivatives and allowed the identification of trends suggesting enrichment of redox-related and thiol-interacting enzyme classes among the most active compounds. These observations are consistent with the proposed GPx-mimetic behavior and support the relevance of the selected molecular scaffold in redox-modulating systems.

#### 2.4.4 Quantum Chemical Calculations (DFT) and Orbital Analysis

Quantum chemical calculations were performed to investigate electronic features associated with redox behavior and GPx-like catalytic activity. All calculations were carried out using the ORCA 5.0 software package. Geometry optimizations were performed at the B3LYP/def2-SVP level of theory, followed by single-point energy calculations using the same functional and basis set. This level of theory has been widely applied for the investigation of organochalcogen compounds and provides a reliable balance between computational cost and accuracy. Frontier molecular orbital energies (E_HOMO and E_LUMO) and HOMO–LUMO energy gaps (ΔE_gap) were calculated for all selected compounds. Orbital spatial distributions were analyzed through visualization of HOMO and LUMO isosurfaces using ChemCraft, enabling qualitative assessment of orbital localization over the chalcogen atom and the conjugated triazole–aryl framework. The results indicate that the most active derivatives (**15a**, **15h**, and **15j**) tend to present higher HOMO energies and reduced HOMO–LUMO gaps compared to less active compounds, suggesting enhanced electron-donating capacity and increased redox flexibility. In addition, HOMO localization over the selenium-containing moiety supports its direct involvement in catalytic redox processes. These findings are consistent with the proposed mechanism of GPx-mimetic activity and provide electronic-level support for the observed experimental trends.

## 3 Results and Discussion

### 3.1 Chemistry

First, the synthesis of the target molecules started by the preparation of hydrazones **19a**–**l**, obtained through a condensation reaction between hydrazines **18a**–**e** and aldehydes **17a**–**h**, both commercially available, according to procedures described in the literature. Hydrazones **19a**–**l** were isolated by recrystallization and obtained as yellow to brown solids, with moderate to excellent yields (40–93%). The melting points obtained were consistent with literature values (Table S1). In addition, the structural identity of the derivatives was corroborated by spectroscopic analyses, in accordance with previously reported data (Scheme 2).^16^

**Scheme 2:**
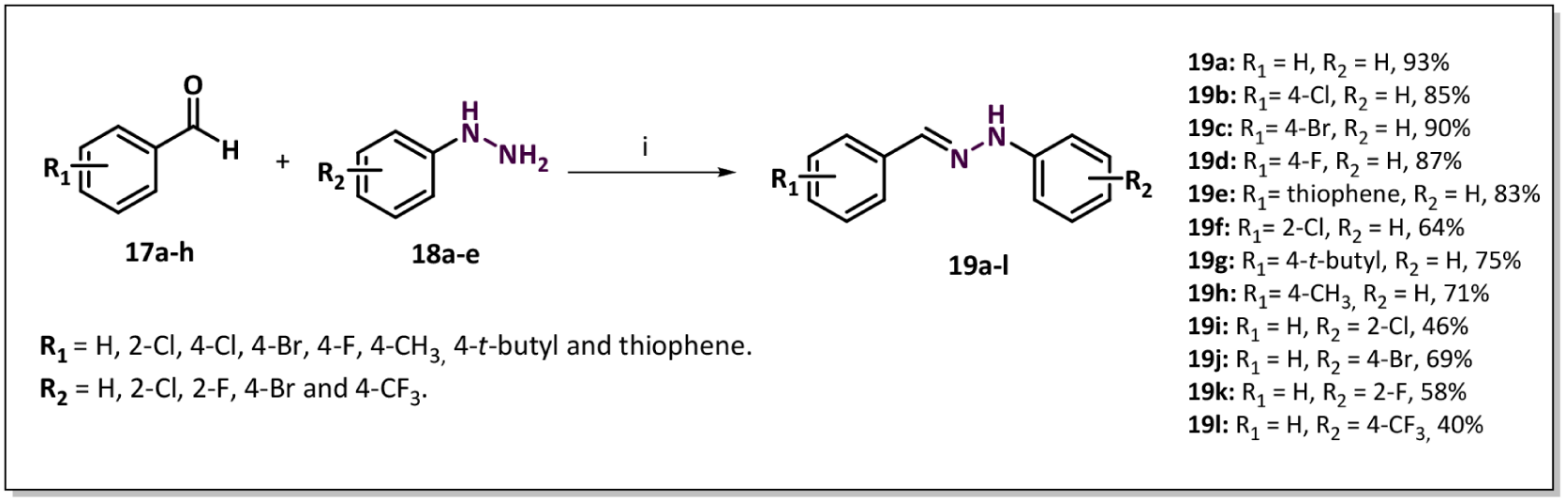
Synthesis of Hydrazones **19a**–**l** ^a^

a Reagents and conditions: (i) CH_2_Cl_2_, r.t., 30 min.

The analysis indicates that electronic and steric effects in the aldehyde portion were not significant, as both electron-donating and electron-withdrawing substituents provided similar yields, consistent with previous reports. In contrast, variations in the hydrazine portion, all bearing electron-withdrawing groups, led to lower yields, likely due to the purity of the commercial reagent.

Inspired by the work of Hao et al.,^16^ the hydrazones were subjected to a tandem reaction involving sequential nitrification, reduction, and cyclization steps to form the 1,2,4-triazole ring. The process employed cobalt nitrate as a redox agent and catalyst, using 1,2-dichloroethane as the solvent at 110 °C for 3 h. At the end of the reaction, the 1,2,4-triazole derivatives **14a**–**l** were purified by column chromatography and obtained predominantly as yellow or orange solids, with moderate to good yields (22–45%). The chemical structures of these compounds were confirmed by spectrometric methods and were consistent with the literature (Scheme 3).

**Scheme 3:**
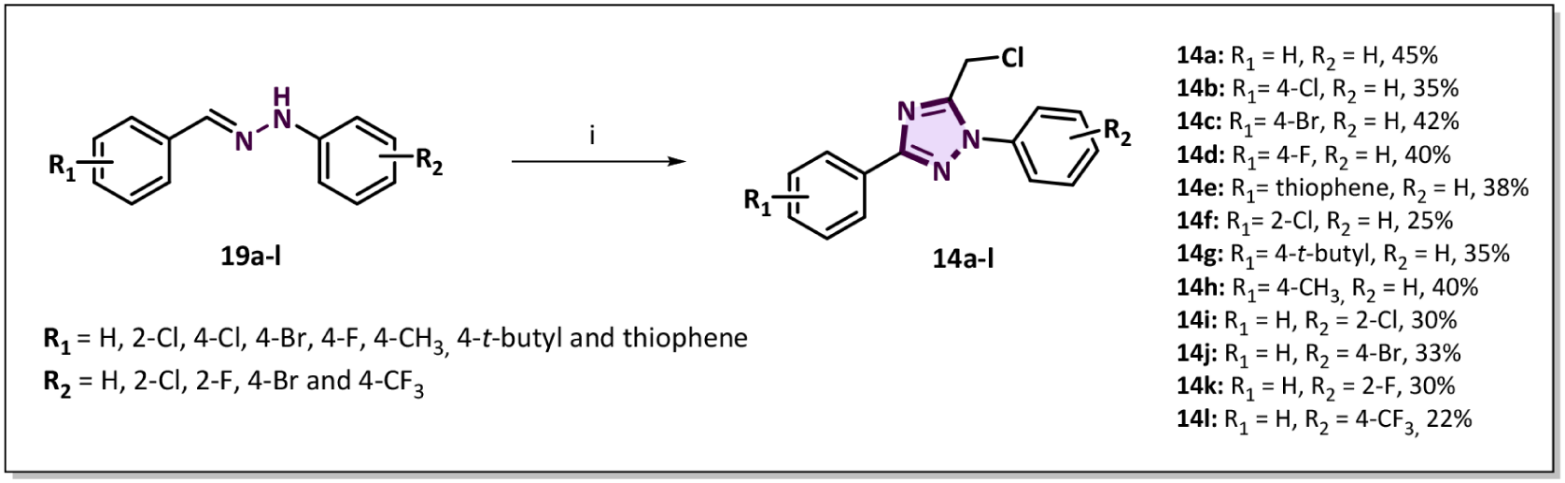
Synthesis of 1,2,4-Triazole Derivatives **14a**–**l** ^a^

a Reagents and conditions: (i) Co­(NO_3_)_2_·6H_2_O, 1,2-dichloroethane, 110 °C, 3 h.

To obtain the selenocyanate compounds, the reaction conditions were initially optimized. Potassium selenocyanate (KSeCN) was chosen as the selenium source, since this reagent is widely used as a versatile precursor, allowing the selective introduction of the selenocyanate group (−SeCN) into activated substrates.^14^ Therefore, the reaction was conducted under different experimental conditions, varying parameters such as temperature, reaction time, amount of nucleophile, and solvent in order to determine the optimal methodology for the formation of the desired product. The results are summarized in Table 1.

**Table 1:**
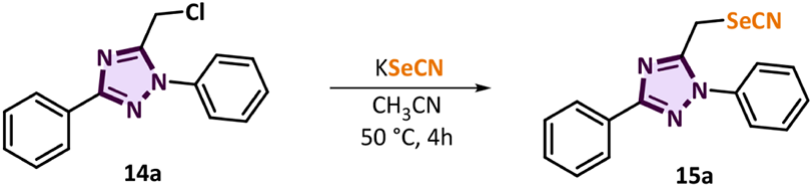
Optimization of Reaction Parameters for the Target compounds^a^

entry	temp. (°C)	KSeCN (mmol)	time (h)	solvent	yield (%)^b^
1	r.t.	0.11	4	CH_3_CN	80
2	r.t.	0.11	6	CH_3_CN	44
**3**	**50**	**0.11**	**4**	**CH**_**3**_ **CN**	**91**
4	100	0.11	4	CH_3_CN	traces
5	50	0.22	4	CH_3_CN	68
6	50	0.055	4	CH_3_CN	traces
7	50	0.11	4	EtOH	58
8	50	0.11	4	DMSO	70
9	50	0.11	4	MeOH	86
10	50	0.11	4	Acetone	68

a Reaction conditions: 1,2,4-triazole **14a** (0.1 mmol) and an open system.

b Isolated yields.

Initially, the reaction was performed at room temperature using 0.1 mmol of substrate **14a** and 0.11 mmol of KSeCN in acetonitrile (CH_3_CN) for 4 h, yielding the product in 80% (entry 1).^24^ In order to evaluate the reaction progress and the possible complete conversion of the substrate, the reaction time was extended to 6 h under the same conditions (entry 2). However, a significant decrease in yield (44%) was observed, due to the formation of byproducts, as detected by thin-layer chromatography (TLC). To evaluate the influence of temperature, the protocol was conducted at 50 and 100 °C (entries 3 and 4) keeping other parameters constant, and under these conditions, the best result was achieved in entry 3, with a 91% yield, indicating that a moderate increase in temperature improved the substrate conversion.^8,26^ The effect of the KSeCN amount was also assessed but was not advantageous (entries 5 and 6). The influence of the solvent was investigated, and among those tested, acetonitrile showed the best performance (Table 1, entries 7–10). This result may be associated with the use of a polar aprotic solvent, which is often beneficial for nucleophilic substitution reactions.^27^ Based on these screenings, the optimal conditions were determined to be 0.1 mmol of 1,2,4-triazole substrate and 0.11 mmol of KSeCN in acetonitrile at 50 °C for 4 h, resulting in the formation of product **15a** in 91% yield (Table 1, entry 3).

After establishing the optimized conditions for the synthesis of the novel 1,3-diphenyl-5-(selenocyanatomethyl)-1*H*-1,2,4-triazole **15a**, the scope of the protocol was evaluated. In the initial phase of our study, reactions were conducted with various 1,2,4-triazole derivatives **14a**–**l**, featuring structural variations in both the aldehyde and hydrazine portions, using KSeCN as the selenium source. Subsequently, with the aim of investigating the influence of the chalcogen on the biological performance, analogous reactions were carried out using potassium thiocyanate (KSCN), leading to the formation of the corresponding sulfur-containing derivatives. This strategy led to the achievement of a diverse series of organochalcogen-containing 1,2,4-triazoles, including selenium derivatives (**15a**–**l**) and sulfur derivatives (**16a**–**c**), with moderate to excellent yields (37–91%), as described in Scheme 4. However, the yields obtained under the present conditions were slightly lower than those reported for related triazole derivatives in the literature, which may be associated with differences in substrate architecture and the specific reaction parameters employed.^7^

**Scheme 4:**
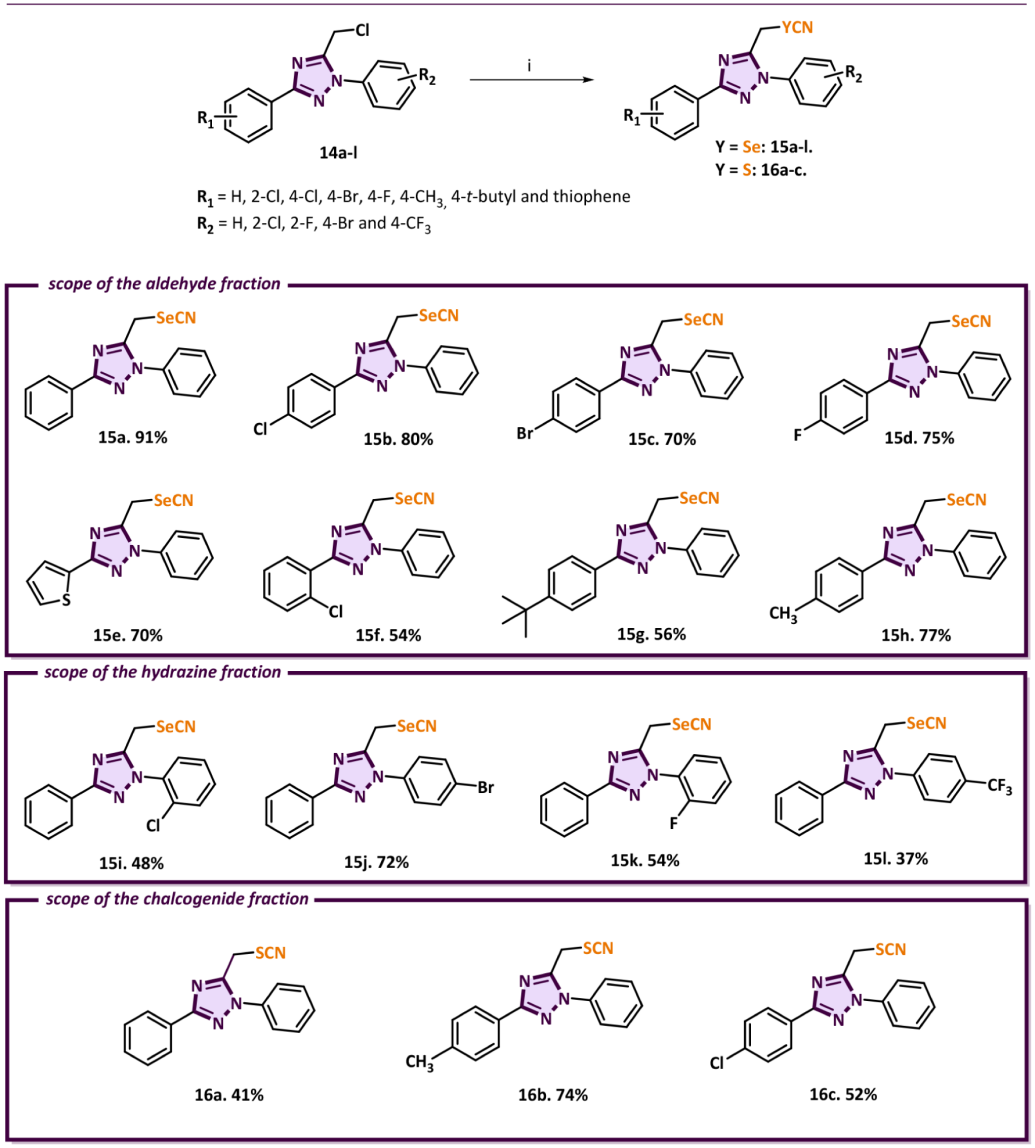
Variation of Reaction Scope^a^

a Reagents and conditions: (i) KYCN, CH_3_CN, 50 °C, 4 h.

As illustrated in Scheme 4, electron-withdrawing substituents at the *para* position of the aldehyde (−Cl, −Br, −F) led to moderate decreases in yields, producing compounds in 80%, 70%, and 75%, respectively. When the halogen (−Cl) was shifted to the *ortho* position **15f**, the yield decreased considerably to 54%, suggesting that *ortho* steric effects hinder product formation.^28^ Bulky substituents, such as *tert*-butyl **15g**, also reduced the yield (56%) probably due to steric hindrance. An electron-donating group like *p-*methyl **15h** led to a good yield (77%), and the thiophene derivative **15e** afforded a 70% yield. In general, electron-withdrawing substituents on the hydrazine moiety, such as 2-Cl (**15i**), 2-F (**15k**), 4-Br (**15j**), and 4-CF_3_ (**15l**), led to a significant decrease in the yields obtained, suggesting that the electron-withdrawing nature of these groups affects the product conversion (48%, 54%, 72% and 37% yields, respectively). The reaction was also performed with potassium thiocyanate, and using this reagent, it was possible to obtain 3 compounds **16a**–**c** with yields ranging from 41% to 74% (Scheme 4).

The structural elucidation of the synthesized chalcogenyl cyanate compounds was carried out through a comprehensive spectroscopic analysis, including proton nuclear magnetic resonance (^1^H NMR), carbon-13 nuclear magnetic resonance ^13^C NMR), as well as high-resolution mass spectrometry (HRMS). As a representative example, the signals assigned to compound **15h** are described as follows (Figure 2). For an accurate structural analysis, two-dimensional NMR experiments, including ^1^H-^13^C HSQC (^1^J_CH_) and ^1^H-^13^C HMBC (^2^J_CH_ and ^3^J_CH_), were employed. The ^1^H NMR spectrum of compound **15h** (Figure S64) exhibits a singlet at 2.40 ppm, attributed to the methyl group attached to the phenyl ring of the aldehyde moiety. Another singlet observed at 4.48 ppm is attributed to the methylene protons bound to the selenocyanate moiety. The aromatic region exhibits signals between 7.26 and 8.01 ppm, where the five protons of the hydrazine moiety ring and the four protons of the aldehyde moiety, characteristic of the substitution, can be observed. The ^13^C NMR spectrum (Figure S65) shows all expected carbon resonances. The signal at 21.07 ppm is attributed to the methylene carbon bonded to the selenocyanate group, while the resonance at 21.49 ppm corresponds to the methyl carbon bonded to the phenyl ring of the aldehyde portion. The signal observed at 101.05 ppm is assigned to the nitrile carbon, which is a characteristic resonance for this functional group. The resonances at 124.91, 129.87, 130.0, and 136.52 ppm are attributed to the phenyl carbons of the hydrazine portion, while the resonances at 126.49, 127.13, 129.43, and 139.94 ppm correspond to the phenyl carbons of the aldehyde moiety. Finally, the resonances at 150.17 and 162.19 ppm are assigned to the triazole-ring carbons. Full details of the spectroscopic data are provided in the Section 2.2 and in the Supporting Information.

**Figure 2:**
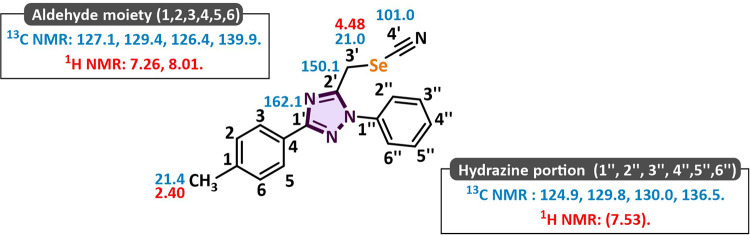
^1^H (in red) and ^13^C (in blue) NMR chemical shifts for compound **15h** (average data).

### 3.2 Evaluation of Gpx-like Catalytic Activity

Glutathione peroxidase (GPx) is a key antioxidant enzyme that is responsible for reducing both organic and inorganic peroxides, thereby protecting biological systems from oxidative stress. Its catalytic mechanism relies on the presence of selenium at the active site, which enables highly efficient redox cycling. In this context, organochalcogen compounds capable of reproducing this redox behavior have been extensively investigated as GPx mimetics due to their biomedical relevance and potential use in chemical transformations that require controlled oxidative processes.^29,30^

With the 15 chalcogenyl-triazole compounds (**15a**–**l** and **16a**–**c**) synthesized and fully characterized, their potential as GPx mimetics was evaluated using the classical UV-based assay commonly applied to estimate the GPx-like activity of chalcogen-containing species.^25,31,32^ In this method, the oxidation kinetics of the thiol cofactor (typically thiophenol, PhSH) are monitored, and the time required for the consumption of 50% of its initial concentration (T_50_) is determined. This parameter enables a comparative assessment of catalytic efficiency among different compounds. Accordingly, T_50_ values were determined for the uncatalyzed reaction, for diphenyl diselenide (reference standard), and for the series of chalcogenyl-triazoles under investigation.^29^

Relative rate values (V_rel_) were calculated using the uncatalyzed reaction as a reference. All compounds were evaluated under identical conditions (methanol, 25 °C), with UV monitoring at 305 nm corresponding to PhSSPh formation. The results, summarized in Table 2, suggest that several chalcogenides may reach or exceed the activity of diphenyl diselenide in this preliminary screening, warranting further kinetic/statistical evaluation under the tested conditions. Among them, compound **15a**, bearing two phenyl substituents on the triazole core, showed the highest activity in the series, with the lowest T_50_ value and a relative rate of 3.2. Derivatives **15b**, **15h**, **15j**, and **16c** also displayed enhanced relative rates (V_rel_ = 1.7–2.8) compared to the reference. Values are reported as mean ± deviation from triplicate measurements (n = 3). These compounds differ mainly in the nature of the aryl substituents on the triazole ring (e.g., methyl, chloro, or bromo groups) and, in some cases, in the identity of the chalcogen atom (Se or S). Within the experimental uncertainty of this assay, selenium–sulfur exchange did not produce pronounced differences in catalytic behavior. In contrast, compounds such as **15c**, **15l**, and **16a** showed lower relative rates than diphenyl diselenide under the conditions tested, consistent with their larger T_50_ values and slower apparent catalytic performance under identical conditions. These comparisons are based on relative T_50_ and V_rel_ values obtained under standardized conditions and are consistent with the approach adopted in the literature for preliminary screening of GPx-mimetic activity.^33^

**Table 2:**
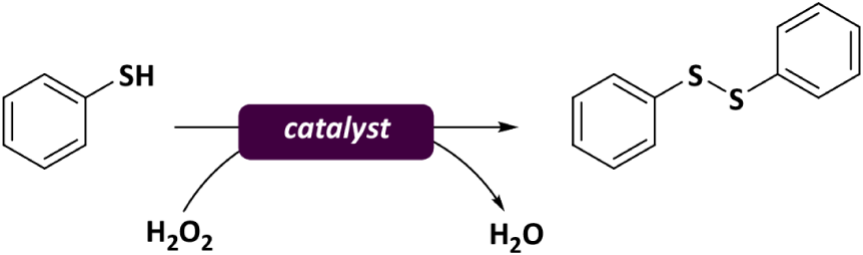
Gpx-like Activities of Organoselenium Catalysts^a^

entry	catalyst	*T*_50_ (h)^b^	*V*_rel_ ^c^
1	–	1.55 ± 0.22	–
2	diphenyl diselenide	0.90 ± 0.05	1.72
3	**15a**	**0.48 ± 0.04**	**3.23**
4	**15b**	**0.88 ± 0.07**	**1.76**
5	**15c**	2.31 ± 0.56	0.67
6	**15d**	1.06 ± 0.02	1.46
7	**15e**	1.27 ± 0.09	1.22
8	**15f**	1.52 ± 0.42	1.02
9	**15g**	1.39 ± 0.06	1.11
10	**15h**	**0.80 ± 0.08**	**1.94**
11	**15i**	3.23 ± 0.88	0.48
12	**15j**	**0.55 ± 0.03**	**2.82**
13	**15k**	1.51 ± 0.34	1.02
14	**15l**	2.46 ± 0.51	0.63
15	**16a**	3.75 ± 0.64	0.41
16	**16b**	3.50 ± 0.75	0.44
17	**16c**	**0.74 ± 0.05**	**2.09**

a Assay conditions: H_2_O_2_ (final concentration = 10.4 mM in MeOH), PhSH (final concentration = 10.0 mM in MeOH), and organoselenium catalyst (final concentration = 10.0 μM in MeOH) at 276.8 ± 0.4 K.

b Values of T_50_ were corrected for the uncatalyzed background reaction and are averages ± standard deviations for three independent runs.

c The relative rate (V_rel_) was calculated with respect to the uncatalyzed reaction.

When compared with previously reported organochalcogen derivatives evaluated under similar thiol oxidation conditions, where T_50_ values typically range from 76 to 300 min, the most active compounds of the present series (e.g., **15a**, **15j**, and **16c**; T_50_ = 29–44 min) exhibit faster oxidation profiles. These observations suggest that the triazole-based scaffold investigated herein provides competitive GPx-like performance relative to earlier reported aryl chalcogen systems under comparable experimental conditions.^31^

Nevertheless, it is important to emphasize that these antioxidant/GPx-like assays were employed as exploratory redox profiling to identify promising scaffolds and structure–activity trends; they do not, by themselves, support claims of clinical suitability or long-term systemic antioxidant use. Further stability, metabolism, and safety assessments are required prior to any translational considerations.

The GPx-like catalytic activity evaluated in this study should be interpreted as an initial screening approach, allowing comparative assessment within the compound series rather than a definitive characterization of enzymatic behavior.

### 3.3 *In Silico* Pharmacokinetic and Drug-Likeness Assessment

On the basis of the experimental GPx-like activity data, a focused subset of compounds was selected to explore potential relationships between redox performance and predicted pharmacokinetic properties. Compounds **15a**, **15j**, and **15h** were chosen as representative high-activity selenocyanate derivatives, whereas compound **16c** was included as a sulfur analogue exhibiting comparable catalytic efficiency. In contrast, compounds **15i**, **15l**, and **16a**, which displayed significantly lower GPx-like activity, were incorporated to serve as low-activity references. This comparative design allowed the identification of trends associated with chalcogen substitution and aromatic functionalization, while avoiding overinterpretation of in silico predictions across the full library. Importantly, the selected subset does not aim to provide a quantitative predictive model but rather to offer qualitative pharmacokinetic and medicinal chemistry insights that complement the experimental redox activity data.

The pharmacokinetic profiles of the selected chalcogen–triazole derivatives were evaluated using a combination of SwissADME and ADMETlab 3.0, with the most relevant physicochemical, pharmacokinetic, and assay-interference parameters summarized in Table 3. Overall, all analyzed compounds exhibited physicochemical properties compatible with orally bioavailable small molecules, including moderate molecular weight, limited hydrogen bond donor capacity, and acceptable polar surface areas.

**Table 3:** Summary of Key *In Silico* ADMET Properties for the Selected Chalcogen–Triazole Derivatives^a^

compound	chalcogen	MW range	TPSA trend	GI absorption	BBB permeation	P-gp substrate	promiscuity/assay interference	major ADMET flags
**15a**	Se	moderate	low	high	yes	yes	low	no genotoxicity
**15h**	Se	moderate	low	high	yes	yes	low	no genotoxicity
**15i**	Se	moderate–high	low	high	yes	yes	low–moderate	no genotoxicity
**15j**	Se	moderate	low	high	yes	yes	low	no genotoxicity
**15l**	Se	high	low	high	yes	yes	low–moderate	no genotoxicity
**16a**	S	moderate	high	high	no	no	low	no genotoxicity
**16c**	S	moderate	high	high	no	no	low	no genotoxicity

a ADMET, absorption–distribution–metabolism–excretion–toxicity; MW, molecular weight; TPSA, topological polar surface area; GI, gastrointestinal; BBB, blood–brain barrier; P-gp, P-glycoprotein; Se, selenium; S, sulfur.

All selected derivatives complied with Lipinski’s rule of five, as well as the Ghose, Veber, Egan, and Muegge filters, with no violations observed, supporting their classification as drug-like chemical entities. In agreement with these findings, all compounds displayed a predicted bioavailability score of 0.55. Analysis of polar surface area revealed a clear distinction between chalcogen classes, with calculated TPSA values of approximately 54.5 Å^2^ for selenocyanate derivatives and 79.8 Å^2^ for thiocyanate analogs, indicating that sulfur incorporation increases molecular polarity and may influence permeability-related properties (Table 3).

Consensus LogP values were calculated by using the SwissADME web server. The numerical values, along with their qualitative classification, are presented in Table 2, allowing direct comparison of lipophilicity across the compound series.^34^ Notably, the most catalytically active GPx mimetics (**15a**, **15j**, **15h**, and **16c**) exhibited balanced lipophilicity, with consensus LogP values between approximately 2.1 and 3.8, whereas less active derivatives tended to display higher lipophilicity (Table S2). This trend suggests that excessive hydrophobicity may adversely affect solvation and accessibility to redox-active sites, thereby limiting the catalytic efficiency.

From a pharmacokinetic standpoint, all analyzed compounds were predicted to exhibit high gastrointestinal absorption, supporting their potential suitability for oral administration. A consistent differentiation between selenium- and sulfur-containing analogs was observed with respect to blood–brain barrier (BBB) permeability and P-glycoprotein (P-gp) substrate prediction. Most selenocyanate derivatives were predicted to be BBB-permeant and P-gp substrates, whereas thiocyanate analogs generally lacked BBB permeability and were not predicted to interact with P-gp. These differences indicate that selenium incorporation may modulate central nervous system exposure and efflux susceptibility, which could be relevant in the context of oxidative stress-related neurological applications.

Predicted cytochrome P450 inhibition involved several isoforms, particularly CYP1A2, CYP2C19, and CYP3A4. Although such liabilities may warrant attention at more advanced stages of drug development, they are frequently associated with aromatic and moderately lipophilic scaffolds and should therefore be interpreted cautiously at this exploratory stage. Importantly, no pan-assay interference compound (PAIN) alerts were identified, and Brenk alerts were limited to motifs intrinsically associated with chalcogen-containing functionalities, which are well documented in organoselenium chemistry.

Overall, the combined SwissADME and ADMETlab 3.0 analyses indicate that the investigated chalcogen–triazole hybrids display favorable drug-likeness, differentiated permeability and efflux profiles, and low predicted assay interference and toxicological risk, thereby supporting the robustness of the experimental GPx-like activity data and their further exploration as redox-active, enzyme-mimetic antioxidant candidates.

Among the analyzed derivatives, compound **15a** was selected as a representative high-activity GPx mimetic to illustrate the overall balance between redox performance and predicted pharmacokinetic behavior. As summarized in Figure 3, the normalized radar plot indicates that the key physicochemical and pharmacokinetic descriptors of **15a** fall predominantly within the optimal ranges associated with orally bioavailable, drug-like small molecules.

**Figure 3:**
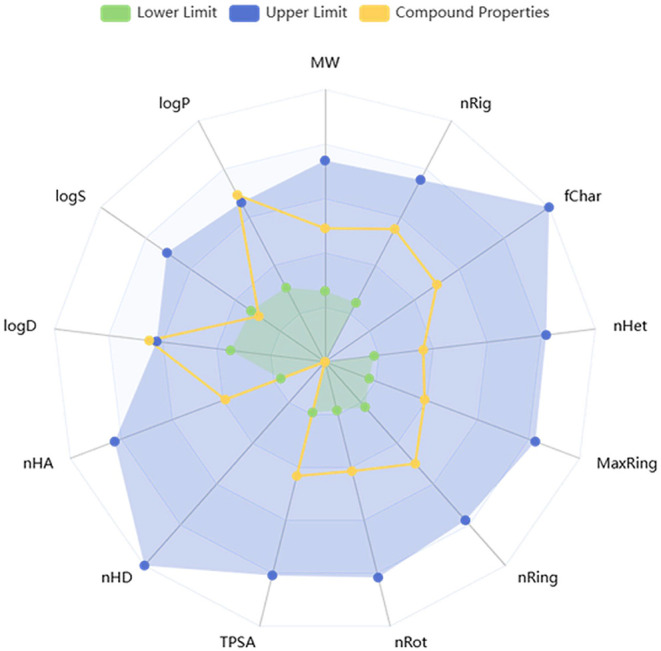
Normalized pharmacokinetic (bioavailability) radar profile of compound **15a**. The plot compares the calculated compound properties of **15a** with the predefined lower limit and upper limit (acceptable boundaries) for each descriptor. Abbreviations: MW, molecular weight; XlogP (or XlogP3), calculated lipophilicity; logS, aqueous solubility; TPSA, topological polar surface area; HBA, hydrogen-bond acceptors; HBD, hydrogen-bond donors; RB (or nROT), number of rotatable bonds; fCsp^3^, fraction of sp^3^ carbons; nRING, number of rings; nAROM, number of aromatic rings; nHET, number of heteroatoms. The compound profile is shown against the predefined limits used to indicate the favorable oral “sweet spot”.

Compound **15a** displays a favorable balance between molecular size, lipophilicity, polarity, flexibility, and saturation, parameters that are commonly associated with efficient membrane permeability and adequate solubility. This balanced profile is consistent with its high predicted gastrointestinal absorption and supports the experimental observation of an enhanced GPx-like catalytic activity.

From a pharmacokinetic standpoint, the radar analysis further suggests that **15a** occupies a well-defined chemical space within the present series, avoiding extreme values that could compromise the developability. Importantly, predicted liabilities related to nonspecific reactivity, assay interference, and promiscuity were low, reinforcing the robustness of the experimental redox data.

Collectively, these features position compound **15a** as a suitable reference structure within the investigated chalcogen–triazole library, providing a rational framework for the interpretation of structure–property relationships and for the future optimization of enzyme-mimetic antioxidant candidates.

#### 3.3.1 *In Silico* Target and Activity Profile Prediction

To gain further qualitative insight into potential mechanistic trends underlying the experimentally observed GPx-like activity, a similarity-based target and activity profile prediction analysis was performed for selected compounds. This approach was used to explore the functional chemical space associated with the most active derivatives without aiming to assign specific molecular targets.

The most catalytically active compounds (**15a**, **15j**, and **15h**) consistently exhibited an enrichment in predicted associations with enzyme classes involved in redox regulation and thiol-dependent processes, including oxidoreductase-related functional categories. In contrast, compounds displaying lower GPx-like activity (**15i**, **15l**, and **16a**) showed a more heterogeneous and less specific distribution of predicted target classes (Table S2), suggesting reduced functional coherence within redox-related enzymatic pathways.

Importantly, these predictions are intended to provide qualitative mechanistic context rather than definitive target identification. The preferential association of the most active derivatives with redox- and thiol-related enzyme classes is nevertheless in good agreement with their experimentally observed GPx-like behavior and supports the proposed enzyme-mimetic antioxidant profile of the chalcogen–triazole scaffold. When considered alongside the predicted pharmacokinetic properties and the electronic and reactivity descriptors obtained from quantum chemical calculations, these results suggest that both electronic structure and functional target-class compatibility contribute to the enhanced catalytic performance of the most active derivatives. Together, the combined in silico analyses reinforce the robustness of the experimental findings and provide a coherent framework for the interpretation of structure–activity relationships within this compound series.

#### 3.3.2 Density Functional Theory and Frontier Orbital Analysis

Density functional theory calculations were carried out to elucidate the electronic factors underlying the GPx-like activity of the chalcogen–triazole derivatives. Representative HOMO and LUMO isosurfaces of compounds **15a** and **16a** are selected for the main text to illustrate (Figure 4) the electronic contrast between the highly active selenocyanate and the weakly active thiocyanate derivatives, while the complete set of frontier orbital surfaces is provided in the Supporting Information. The calculated frontier molecular orbital energies, including HOMO, LUMO, and the corresponding HOMO–LUMO energy gaps (ΔE_gap_), are summarized in Table 4.

**Figure 4:**
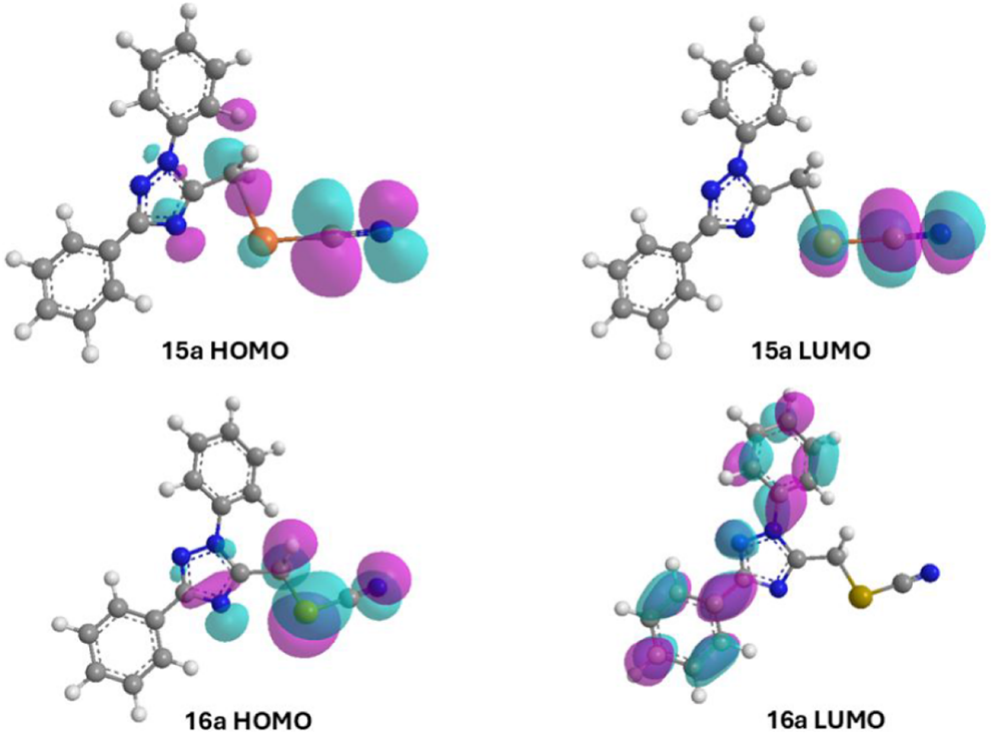
Frontier molecular orbital isosurfaces of representative chalcogen–triazole derivatives. HOMO and LUMO isosurfaces of the highly active selenocyanate derivative **15a** and the weakly active thiocyanate analog **16a**, obtained from DFT calculations. The HOMO of compound **15a** is predominantly localized on the selenocyanate–triazole fragment, whereas compound **16a** exhibits a more delocalized frontier orbital distribution, consistent with their distinct GPx-like activities.

**Table 4:** Frontier Molecular Orbital Energies of Selected Chalcogen–Triazole Derivatives

compound	chalcogen	*E*_HOMO_ (eV)	*E*_LUMO_ (eV)	Δ*E*_gap_ (eV)
**15a**	Se	–7.53	–2.23	5.30
**15h**	Se	–7.58	–2.14	5.44
**15j**	Se	–7.72	–2.03	5.69
**15i**	Se	–7.85	–2.24	5.61
**15l**	Se	–7.66	–2.53	5.13
**16c**	S	–7.63	–0.57	7.06
**16a**	S	–10.07	–2.33	7.74

In addition to the spatial distribution of the frontier orbitals, the calculated orbital energies provide further insight into the observed activity trends. The most catalytically active selenocyanate derivatives (**15a**, **15h,** and **15j**) exhibit relatively higher HOMO energies and moderate HOMO–LUMO energy gaps, indicative of enhanced electronic flexibility and a greater propensity for electron donation during redox processes. In contrast, the less active derivatives, particularly compound **16a**, display a markedly stabilized HOMO and a significantly larger HOMO–LUMO gap, suggesting reduced redox reactivity and electronic adaptability. From a mechanistic standpoint, a smaller HOMO–LUMO gap is commonly associated with higher electronic softness/polarizability and a lower energetic penalty for charge transfer, which favors the electron-transfer steps required for catalytic redox cycling in GPx-like mimetics. Conversely, an increased ΔE_gap_ reflects a more electronically rigid system, typically less able to engage in efficient charge redistribution during peroxide reduction and thiol oxidation. This rationale explains the weaker performance of compound **16a**: it shows the largest ΔE_gap_ in the set (7.74 eV) together with a strongly stabilized HOMO (E_HOMO_ = −10.07 eV), indicating reduced electron-donor ability and diminished propensity to participate in the redox steps that sustain GPx-like turnover, consistent with its lower experimental activity (V_rel_ = 0.41).

For the most catalytically active selenocyanate derivatives (**15a**, **15h**, and **15j**), the HOMO is predominantly localized on the selenocyanate moiety and the adjacent 1,2,4-triazole ring, indicating that the chalcogen-containing fragment acts as the principal electron-donating center. This spatial localization supports the direct involvement of the selenium atom in the redox cycle associated with the GPx-like activity. In contrast, less active selenocyanate derivatives (**15i** and **15l**) exhibit a more diffuse HOMO distribution, with an increased contribution from the aromatic substituents and reduced localization on the selenocyanate group, suggesting diminished redox participation of the selenium center.

The corresponding LUMO orbitals for the active compounds are more delocalized over the triazole and aromatic frameworks, which may facilitate the stabilization of the oxidized state during catalysis. The sulfur-containing analog **16c** displays a distinct electronic profile, characterized by a higher LUMO energy and a more localized HOMO, indicating a different yet still viable redox behavior. Conversely, compound **16a** presents highly delocalized frontier orbitals combined with an increased HOMO–LUMO gap, consistent with its reduced experimental GPx-like activity.

Overall, the combined analysis of frontier orbital energies (Table 4) and spatial orbital distributions (Figure 4) indicates that effective GPx-like activity is associated not only with favorable HOMO energies and reduced HOMO–LUMO gaps but also with the localization of the HOMO on the chalcogen-containing moiety, highlighting the central role of electronic structure in modulating enzyme-mimetic antioxidant performance.

The integration of experimental and computational data provides a coherent structure–activity framework for the investigated compounds. The most active derivatives (**15a**, **15h,** and **15j**) displayed favorable pharmacokinetic profiles, absence of critical structural alerts, and electronic features associated with enhanced redox reactivity, including higher HOMO energies and reduced HOMO–LUMO gaps. These combined characteristics suggest that both electronic and physicochemical properties contribute to the observed GPx-like catalytic activity, reinforcing the potential of chalcogen–triazole hybrids as tunable redox-active scaffolds for medicinal chemistry applications.

## 4 Conclusions

In this study, a structurally diverse series of 15 selenium- and sulfur-containing 1,2,4-triazole hybrids was rationally designed, synthesized, and evaluated as enzyme-mimetic antioxidants with glutathione peroxidase (GPx)-like activity. An efficient and scalable synthetic approach enabled the rapid generation of both selenocyanate and thiocyanate derivatives, providing a flexible platform for structure–activity relationship exploration and future lead optimization.

The experimental GPx-like activity assessment identified several derivatives with catalytic performance superior to the reference compound diphenyl diselenide, demonstrating the potential of the chalcogen–triazole scaffold as a source of functional antioxidant leads. Compounds **15a**, **15h**, and **15j** emerged as the most active members of the series, while the activity observed for the sulfur-containing analog **16c** highlights that appropriate electronic and structural tuning can partially compensate for chalcogen substitution. These results indicate that both chalcogen identity and peripheral aromatic functionalization can be strategically modulated to optimize redox performance.

From a drug discovery perspective, the integration of in silico pharmacokinetic and drug-likeness analyses revealed that the most active compounds occupy a favorable lead-like chemical space, comply with established medicinal chemistry guidelines, and exhibit predicted oral bioavailability, low assay interference risk, and manageable pharmacokinetic liabilities. The differentiated permeability and efflux profiles observed between selenium- and sulfur-containing derivatives further suggest opportunities for rational modulation of tissue exposure, including potential central nervous system applications relevant to oxidative stress-driven pathologies.

Mechanistic insight provided by density functional theory calculations established a clear link between electronic structure and catalytic efficiency. Enhanced GPx-like activity correlated with higher HOMO energies, moderate HOMO–LUMO gaps, and spatial localization of the HOMO on the chalcogen–triazole fragment, supporting the concept that redox flexibility and controlled electron donation are critical determinants of enzyme-mimetic behavior. These electronic descriptors provide valuable design criteria for the next generation of redox-active triazole derivatives.

Taken together, this work positions chalcogen–triazole hybrids as a promising and tunable molecular class for the development of small-molecule enzyme-mimetic antioxidants. In particular, compound **15a** represents a compelling lead candidate, combining high catalytic efficiency with a balanced electronic and pharmacokinetic profile. The results presented herein lay the groundwork for subsequent translational studies, including cellular antioxidant evaluation, target engagement assessment, and lead optimization aimed at advancing these compounds toward biologically relevant models of oxidative stress and redox imbalance.

## Supplementary Material



## Data Availability

The data underlying this study are available in the published article and its online Supporting Information.

## Notes

Melting points and spectroscopic characterization for the synthesized compounds; in silico pharmacokinetic analysis; computational details; frontier molecular orbital analysis; Figure S1 to S89; Tables S1 and S2 (PDF)
