# Supplementary material for: Design and Synthesis of Novel Chalcogen-1,2,4-triazoles as Lead-like Glutathione Peroxidase-Mimetic Antioxidants
Source: ACS Omega. 2026 Jun 25;11(26):38553–66. doi: 10.1021/acsomega.6c00392 (PMC13347327; doi:10.1021/acsomega.6c00392)
Supplement: Supplementary file 1 [file ao6c00392_si_001.pdf]

Electronic Supplementary Information (ESI) for

Design and Synthesis of Novel Chalcogen-1,2,4-Triazoles as Lead-Like Glutathione Peroxidase (GPx)-Mimetic Antioxidants

Nathália L. B. Santos,[a] Luana S. Gomes,[a] Nathalia B. Sá,[a] Pâmella Cordeiro,[b] Aldo S. de Oliveira*[c] and Vanessa Nascimento*[a]

[a]*SupraSelen Laboratory, Department of Organic Chemistry, Universidade Federal Fluminense, Institute of Chemistry, Campus of Valonguinho, 24020-141, Niterói-RJ, Brazil.*
[b]*LabSelen, Department of Chemistry, Universidade Federal de Santa Catarina, 88040-900, Florianópolis-SC, Brazil*
[c]*Instituto Gulbenkian Institute de Medicina Molecular (GIMM), Faculdade de Medicina, Universidade de Lisboa, 1649-028, Lisboa, Portugal.*

**Contents**	**Page**
Melting points and Spectroscopic characterization for the Synthesized Compounds	**S2**
In silico pharmacokinetic analysis	**S54**
Computational details	**S56**
Frontier molecular orbital analysis	**S57**

S1. Melting points and Spectroscopic characterization for the Synthesized Compounds

Tabela S1. Melting points of hydrazones.

Compounds	Experimental Melting Point (°C)	Melting Point (literature) (°C)	Physical appearance
**19a**	155-158°C	155-157 °C [1]	yellow solid
**19b**	126-128°C	126-128°C [2]	white solid
**19c**	121-124°C	120-122°C [2]	brownish solid
**19d**	139-142°C	145-147°C [2]	brownish solid
**19e**	138-141°C	139-141°C [3]	yellow solid
**19f**	86-88°C	82-84°C [4]	yellow solid
**19g**	144-146°C	-	yellow solid
**19h**	109-112°C	110-112°C [5]	brownish solid
**19i**	72-75°C	73°C [6]	pink solid
**19j**	119-122°C	125°C [7]	brownish solid
**19k**	101-103°C	-	brownish solid
**19l**	127-131°C	-	pink solid

---

[1] Yatham, V. R.; Harnying, W.; Kootz D.; Neudörfl, J. M.; Schlörer, N. E.; Berkessel, A. 1,4-Bis-Dipp/Mes-1,2,4-Triazolylidenes: Carbene Catalysts That Efficiently Overcome Steric Hindrance in the Redox Esterification of α- and β-Substituted α, β-Enals. *J. Am. Chem. Soc.,* **2016**, *138*, 2670–2677.

[2] Zabaleta, N.; Uria, U.; Reyes, E.; Carrillo, L.; Vicario, J. L., Ion-Pairing Catalysis in the Enantioselective Addition of Hydrazones to N-Acyldihydropyrrole Derivatives**.** *Chem. Commun.,* **2018**, *54*, 8905-8908.

[3] La Regina, G.; Gatti, V.; Piscitelli, F.; Silvestri, R. Open Vessel and Cooling while Heating Microwave-Assisted Synthesis of Pyridinyl *N*-Aryl Hydrazones. *ACS. Comb. Sci.*, **2011**, *13,* 1, 2–6.

[4] Kaupp, G. Waste-free synthesis and production all across chemistry with the benefit of self-assembled crystal packings. *J. Phys. Org. Chem.,* **2008**, *21, 630.*

[5] Hania, M.M. Synthesis and Antibacterial Activity of Some Transition Metal Complexes of Oxime, Semicarbazone and Phenylhydrazone. *J. Chem*., **2009**, *6*, 204714.

[6] Busch, M. *J. Prakt. Org. Chem.,* **1936**, *144*, 291-312.

[7] Purkait, A.; Jana, C. K. N-Aminations of Benzylamines and Alicyclic Amines with Nitrosoarenes to Hydrazones and Hydrazides. *Synthesis*, **2019**, *51*, 2687-2696.

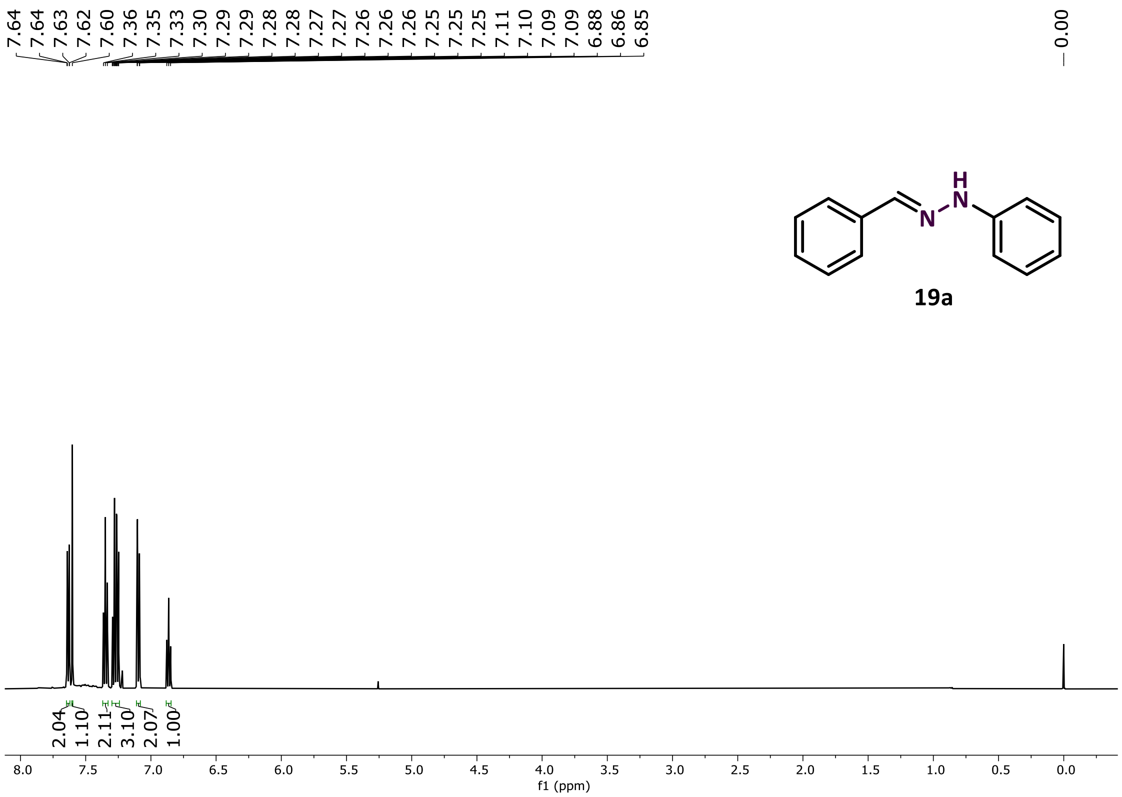

Figure S1. ^{1}H NMR spectrum of compound **19a** in $CDCl_3$ at 500 MHz.

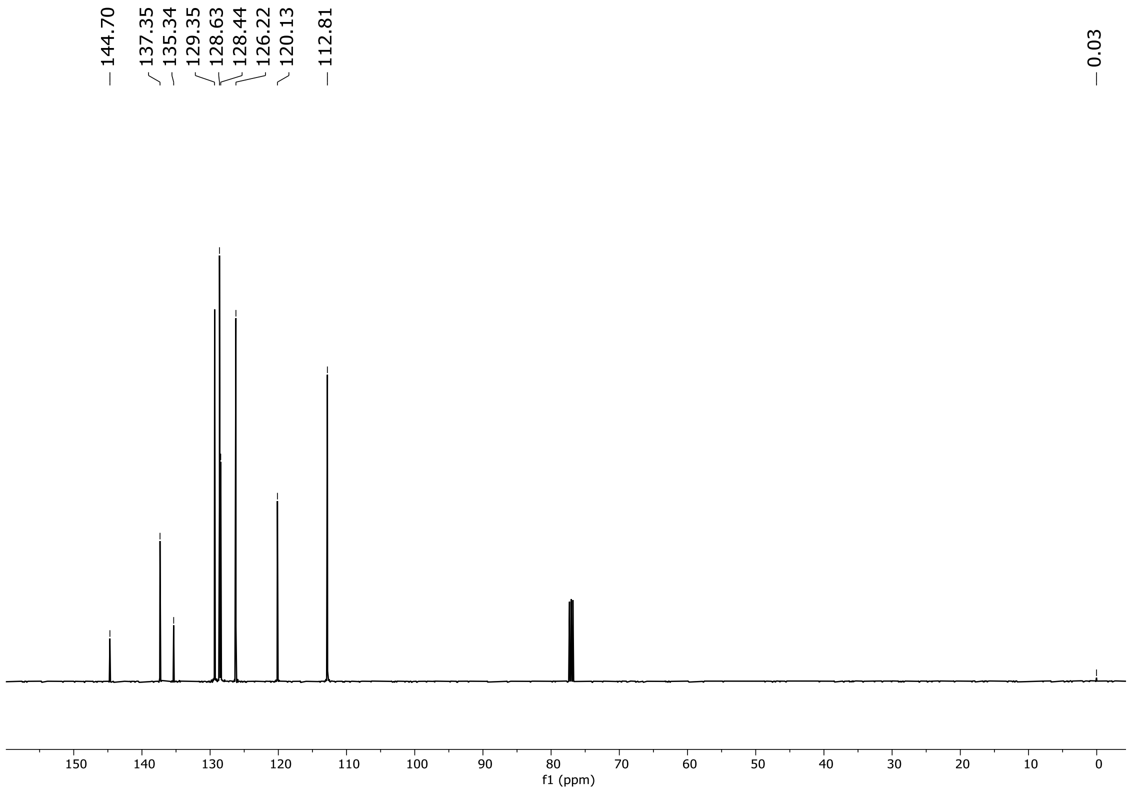

Figure S2. ^{13}C NMR spectrum of compound **19a** in $CDCl_3$ at 125 MHz.

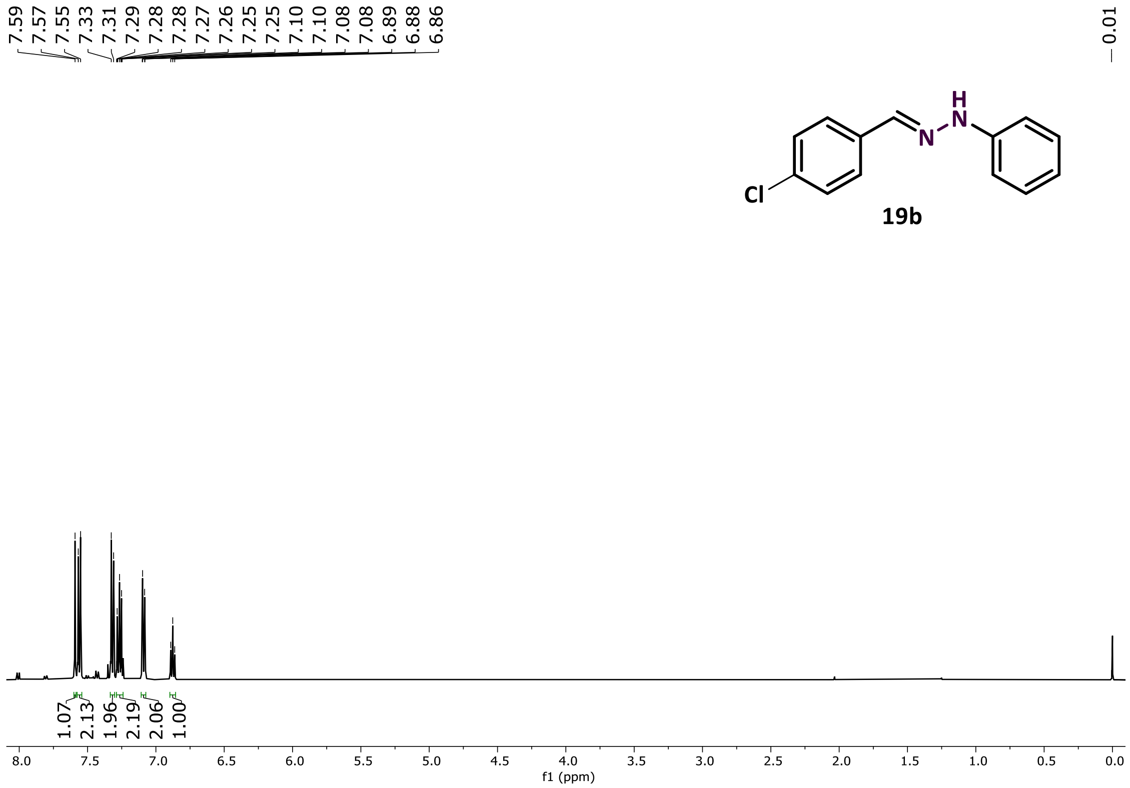

Figure S3. ^{1}H NMR spectrum of compound **19b** in $CDCl_3$ at 500 MHz.

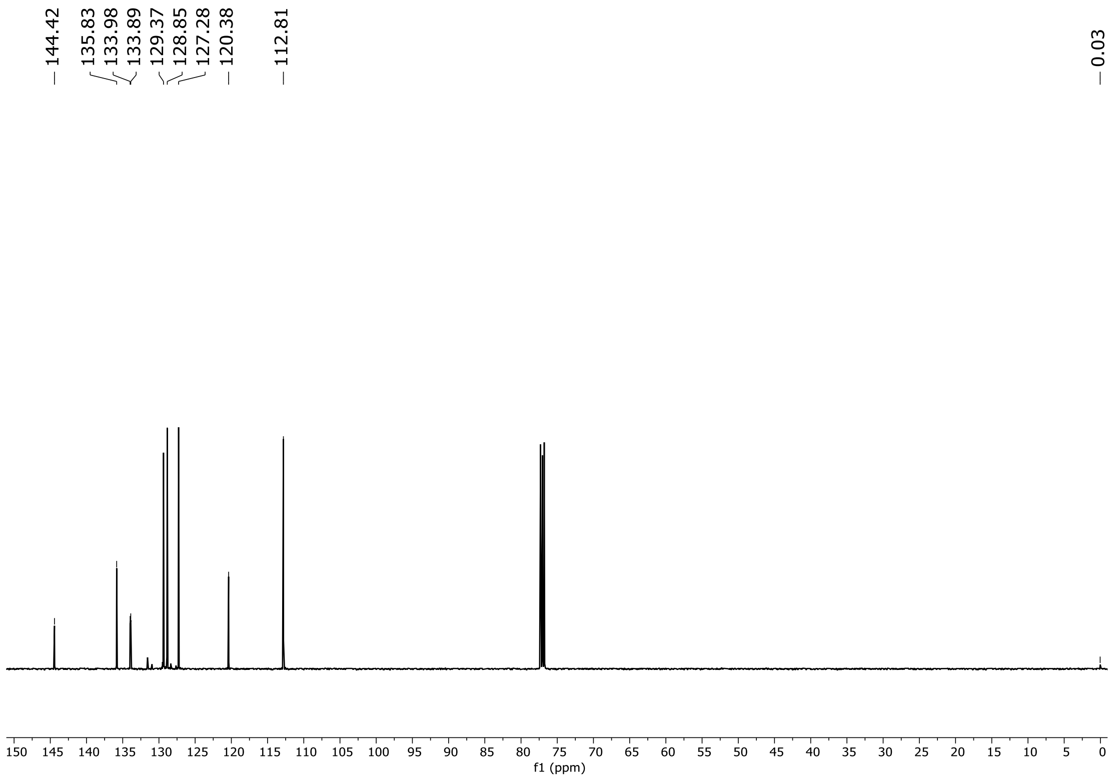

Figure S4. ^{13}C NMR spectrum of compound **19b** in $CDCl_3$ at 125 MHz.

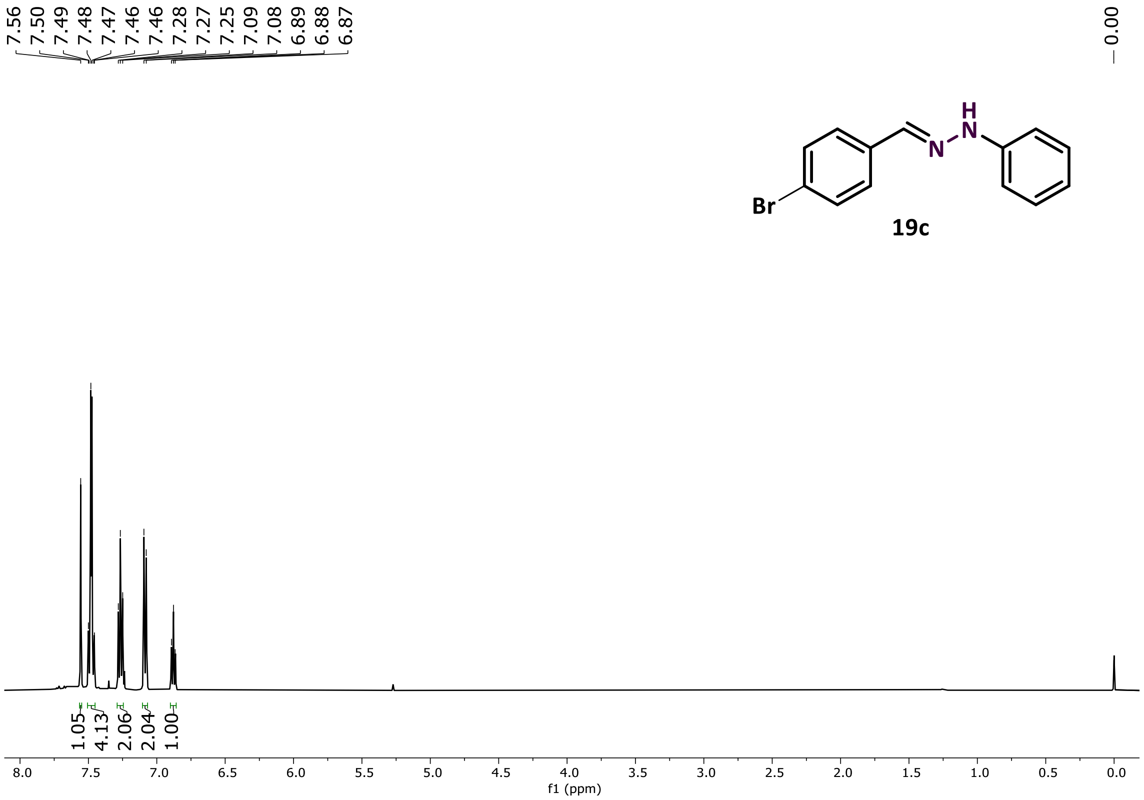

Figure S5. ^{1}H NMR spectrum of compound **19c** in $CDCl_3$ at 500 MHz.

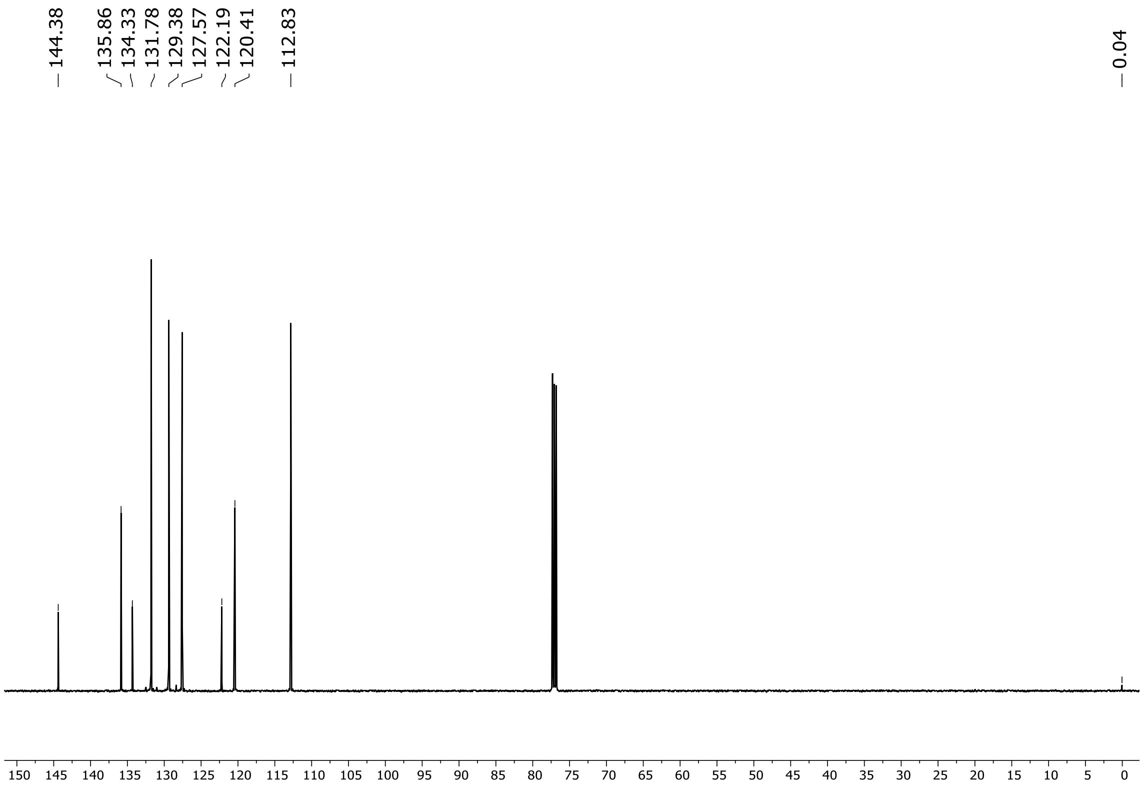

Figure S6. ^{13}C NMR spectrum of compound **19c** in $CDCl_3$ at 125 MHz.

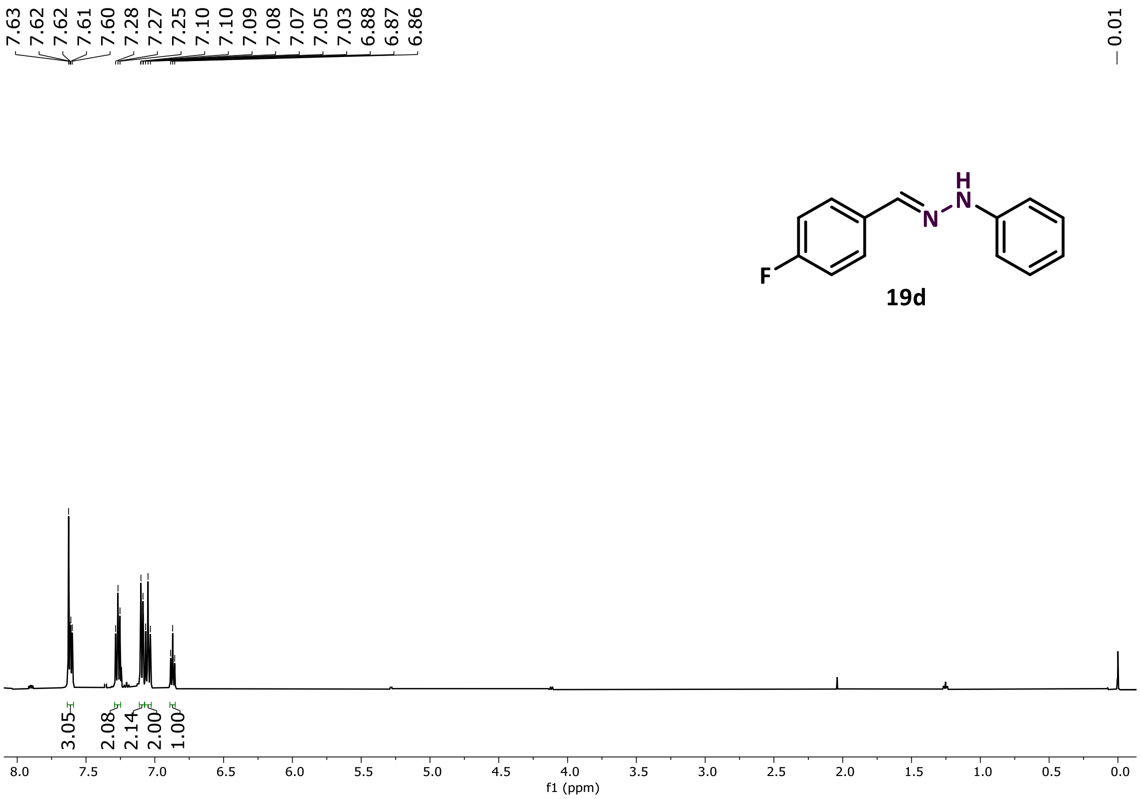

Figure S7. ^{1}H NMR spectrum of compound **19d** in $CDCl_3$ at 500 MHz.

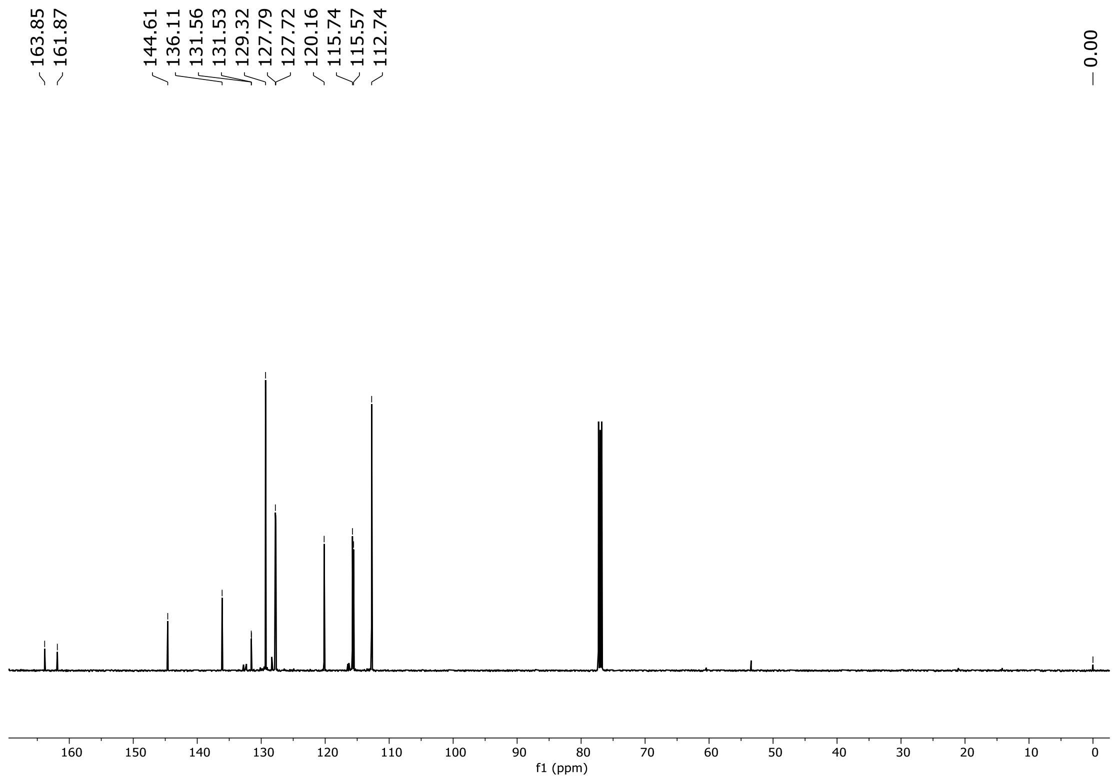

Figure S8. ^{13}C NMR spectrum of compound **19d** in $CDCl_3$ at 125 MHz.

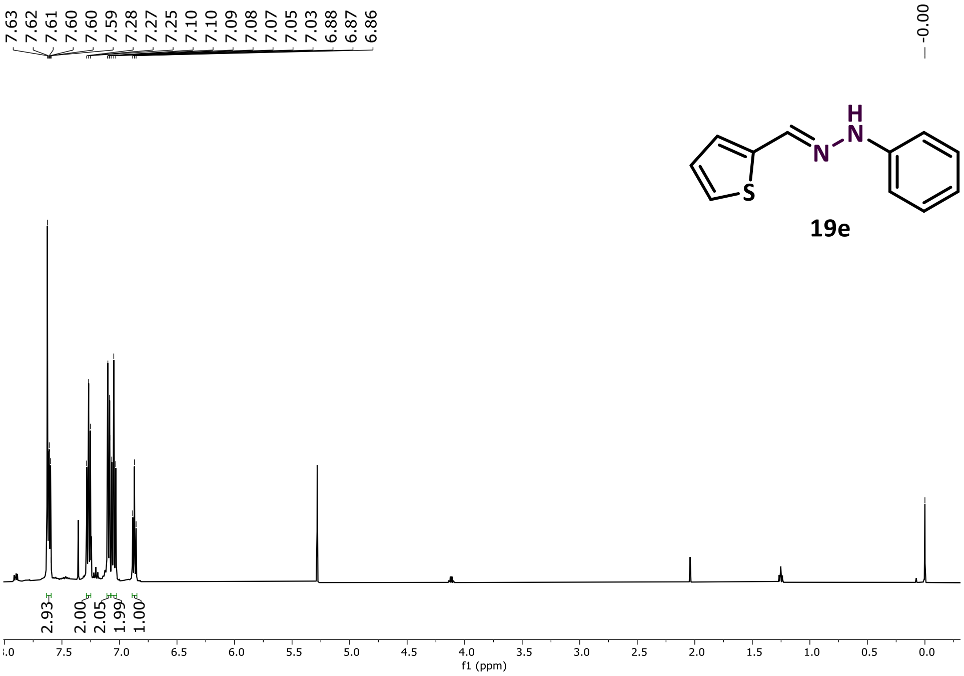

Figure S9. ^{1}H NMR spectrum of compound **19e** in $CDCl_3$ at 500 MHz.

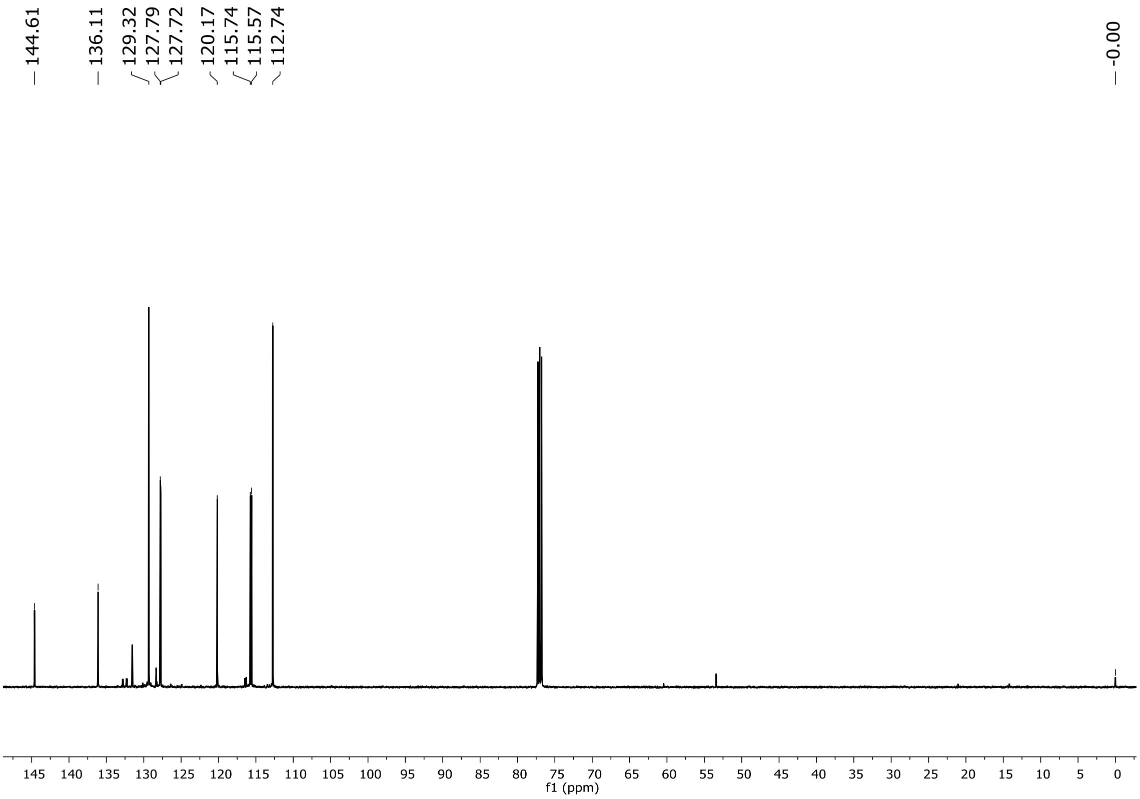

Figure S10. ^{13}C NMR spectrum of compound **19e** in $CDCl_3$ at 125 MHz.

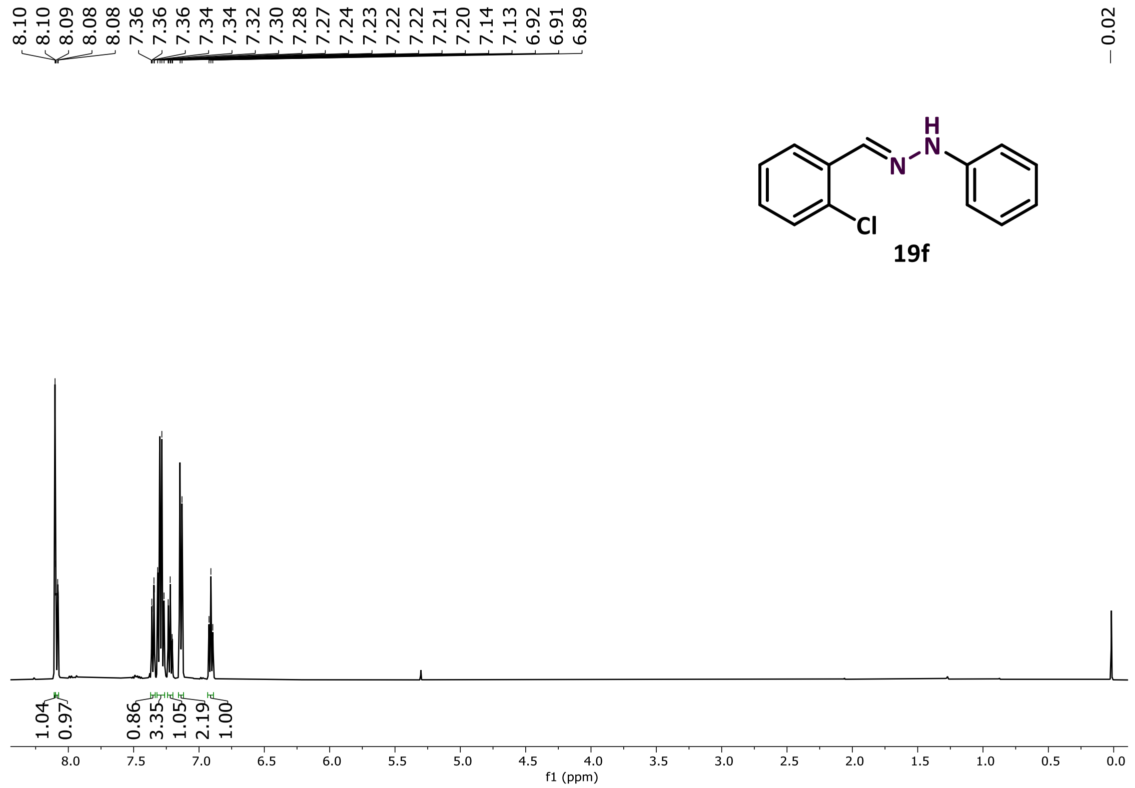

Figure S11. ^{1}H NMR spectrum of compound **19f** in $CDCl_3$ at 500 MHz.

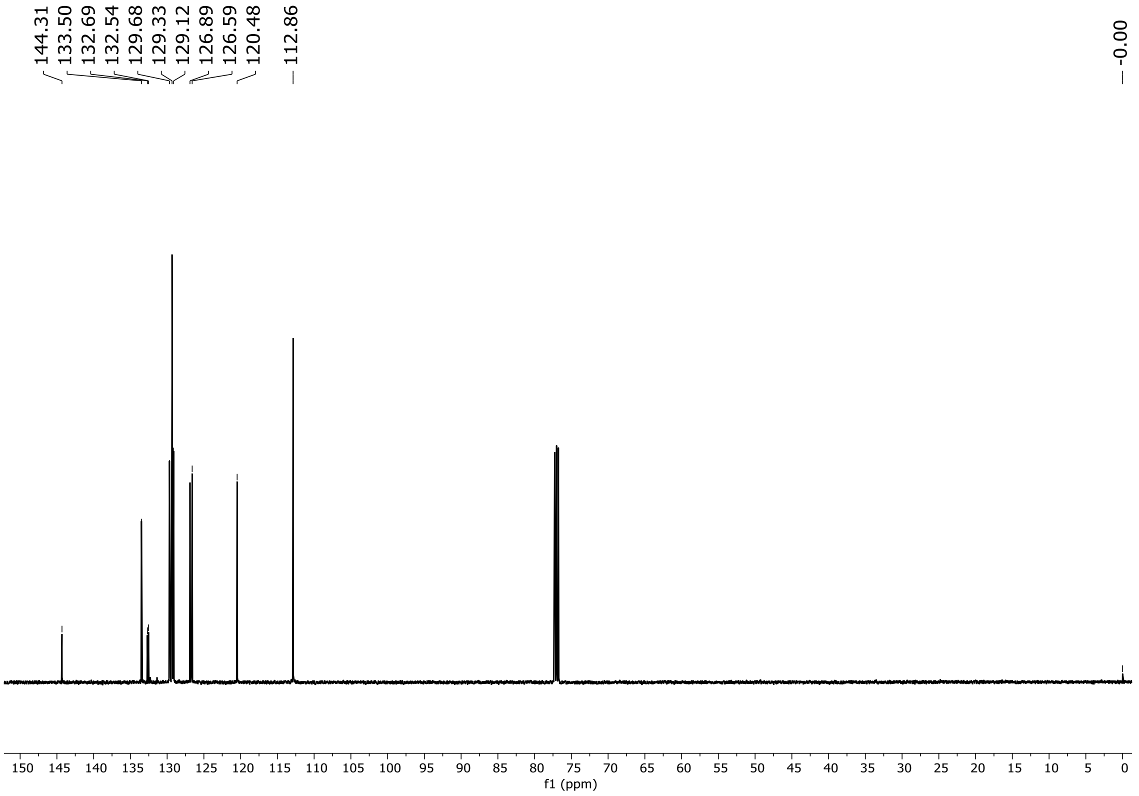

Figure S12. ^{13}C NMR spectrum of compound **19f** in $CDCl_3$ at 125 MHz.

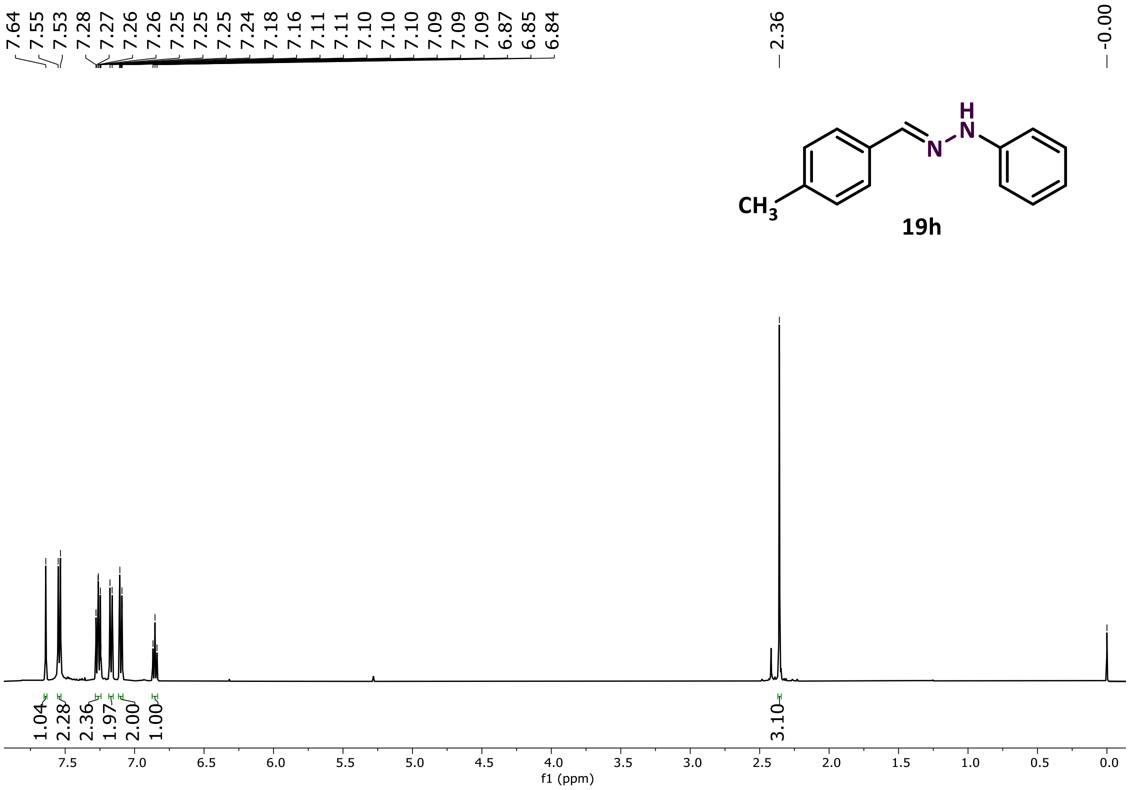

Figure S13. ^{1}H NMR spectrum of compound **19h** in $CDCl_3$ at 500 MHz.

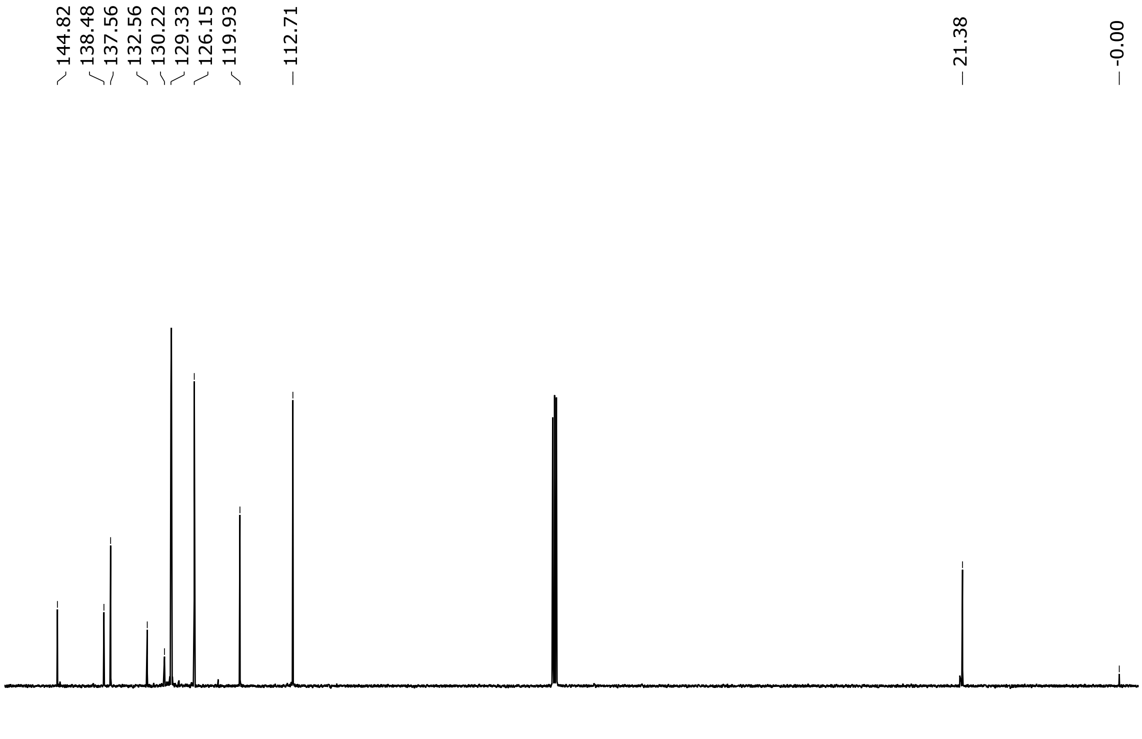

Figure S14. ^{13}C NMR spectrum of compound **19h** in $CDCl_3$ at 125 MHz.

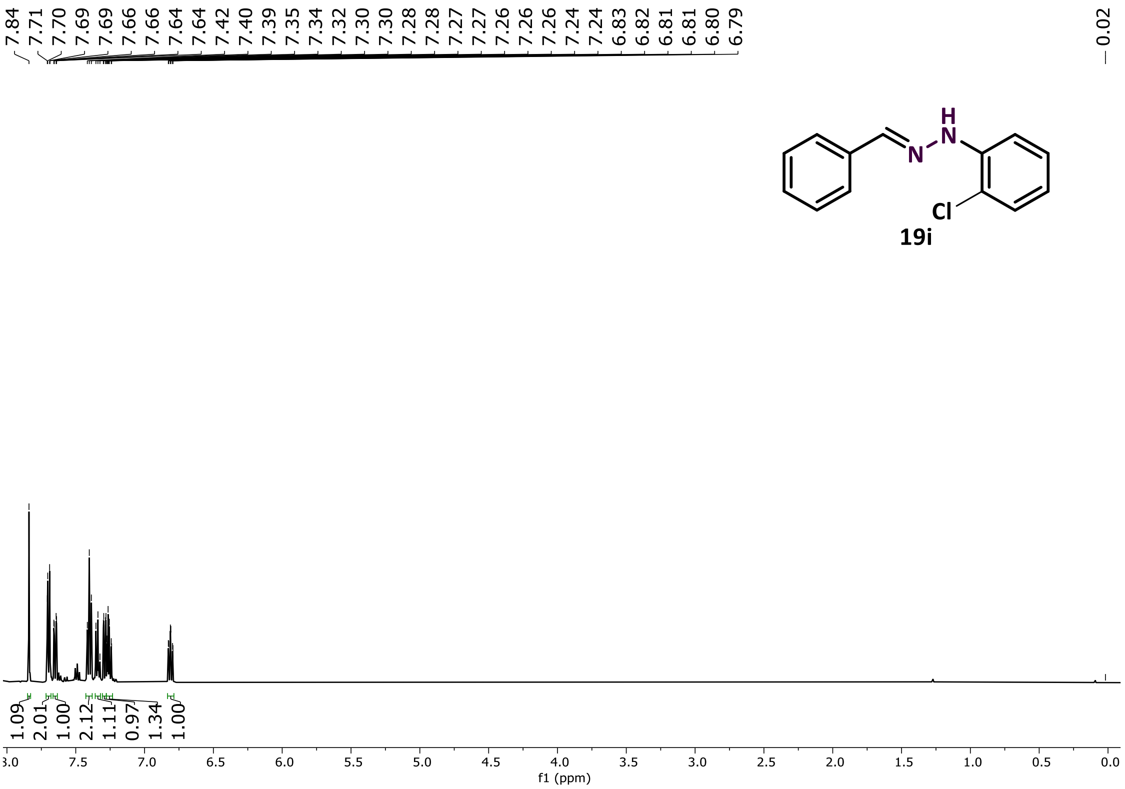

Figure S15. ^{1}H NMR spectrum of compound **19i** in $CDCl_3$ at 500 MHz.

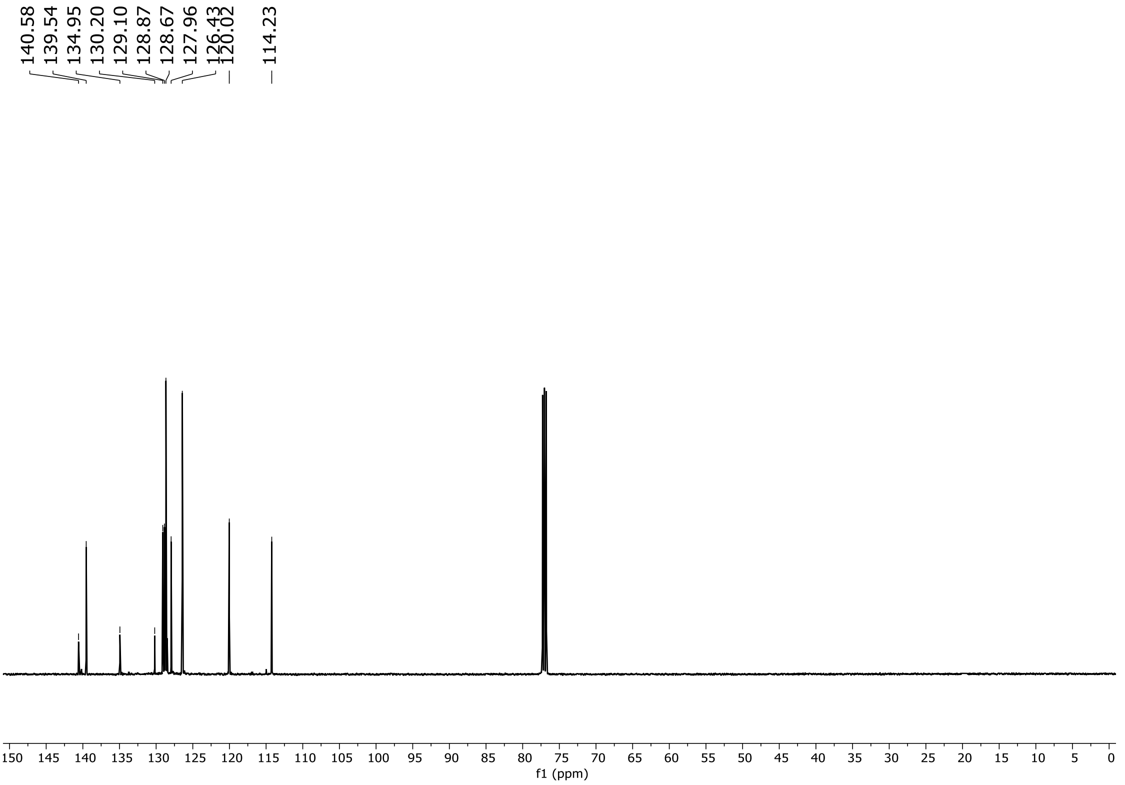

Figure S16. ^{13}C NMR spectrum of compound **19i** in $CDCl_3$ at 125 MHz.

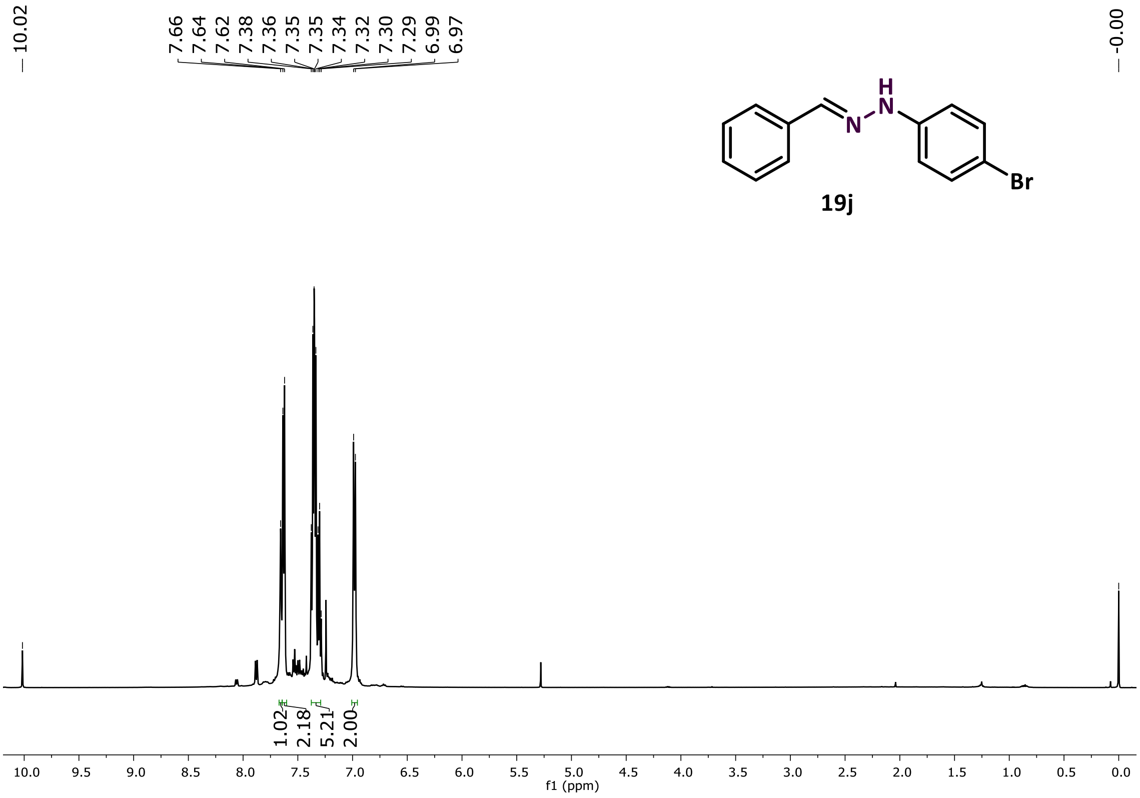

Figure S17. ^{1}H NMR spectrum of compound **19j** in $CDCl_3$ at 500 MHz.

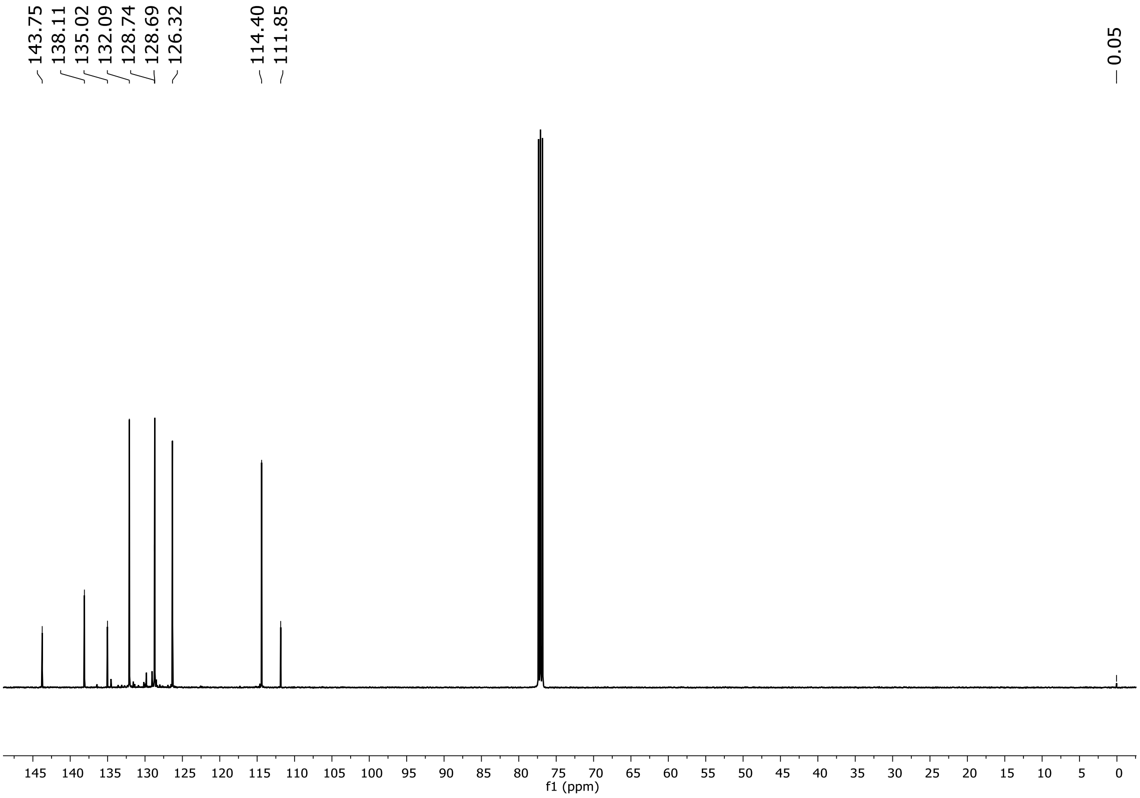

Figure S18. ^{13}C NMR spectrum of compound **19j** in $CDCl_3$ at 125 MHz.

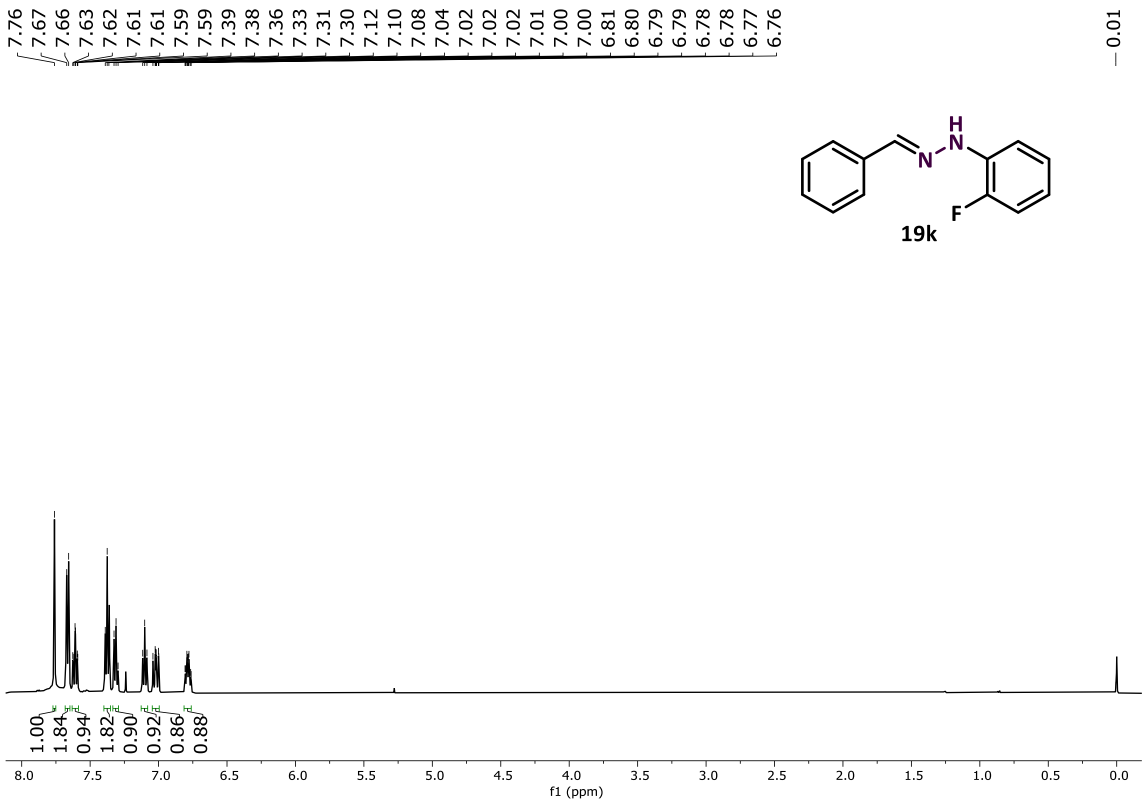

Figure S19. ^{1}H NMR spectrum of compound **19k** in $CDCl_3$ at 500 MHz.

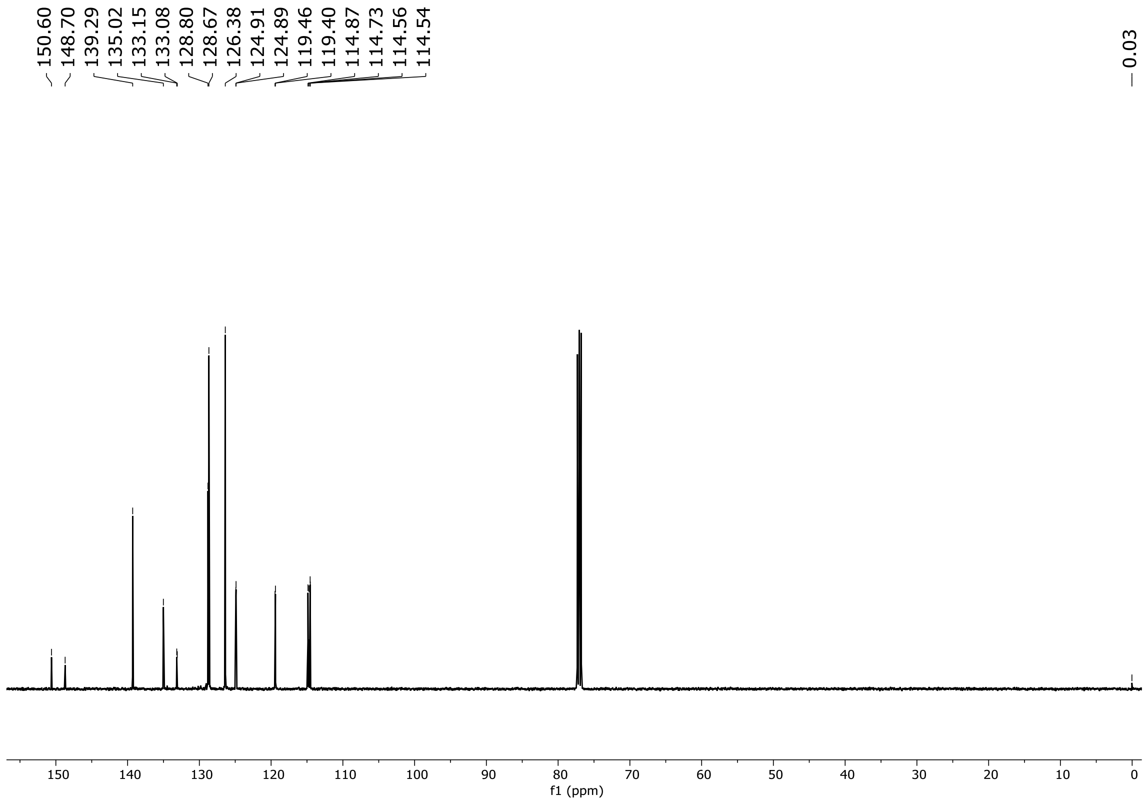

Figure S20. ^{13}C NMR spectrum of compound **19k** in $CDCl_3$ at 125 MHz.

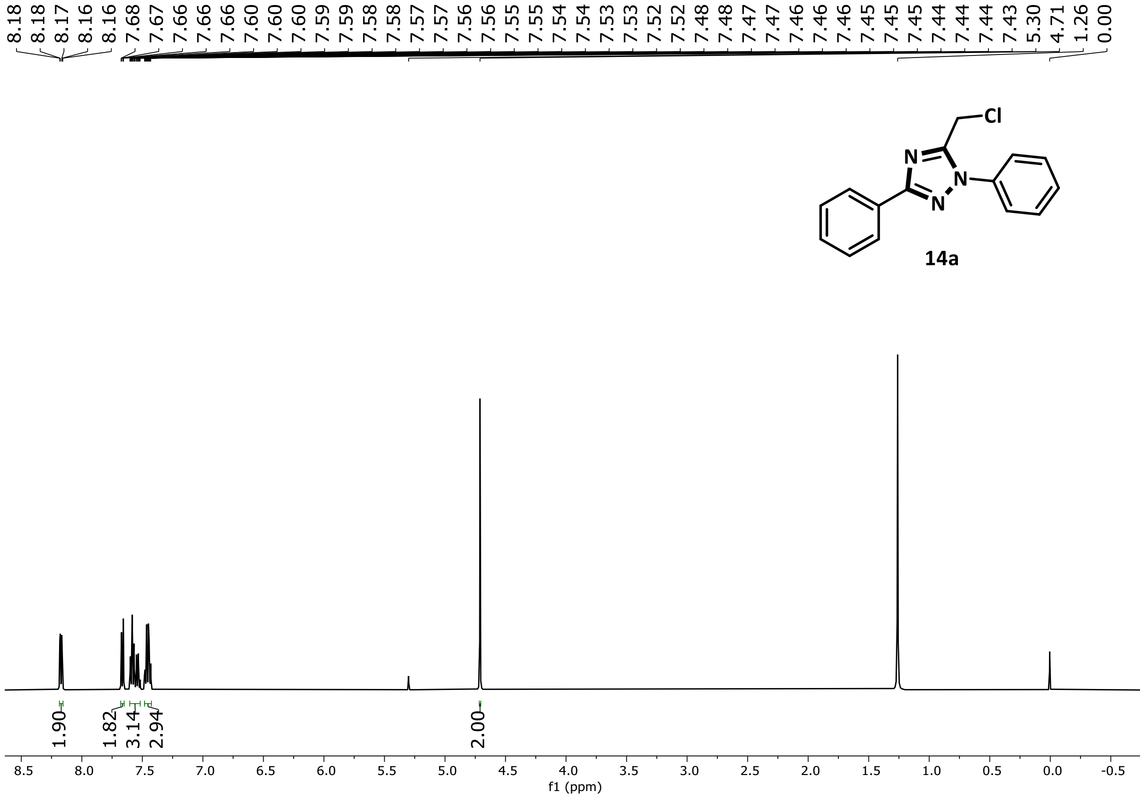

Figure S21. ^{1}H NMR spectrum of compound **14a** in $CDCl_3$ at 500 MHz.

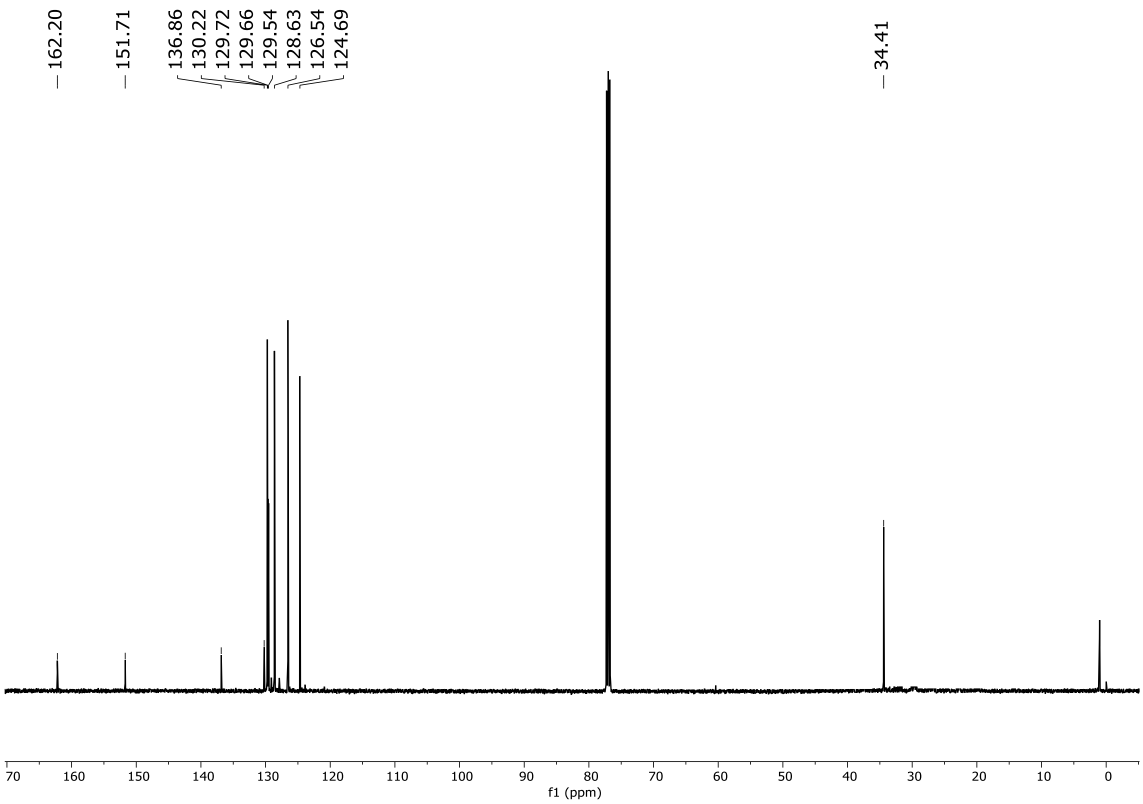

Figure S22. ^{13}C NMR spectrum of compound **14a** in $CDCl_3$ at 125 MHz.

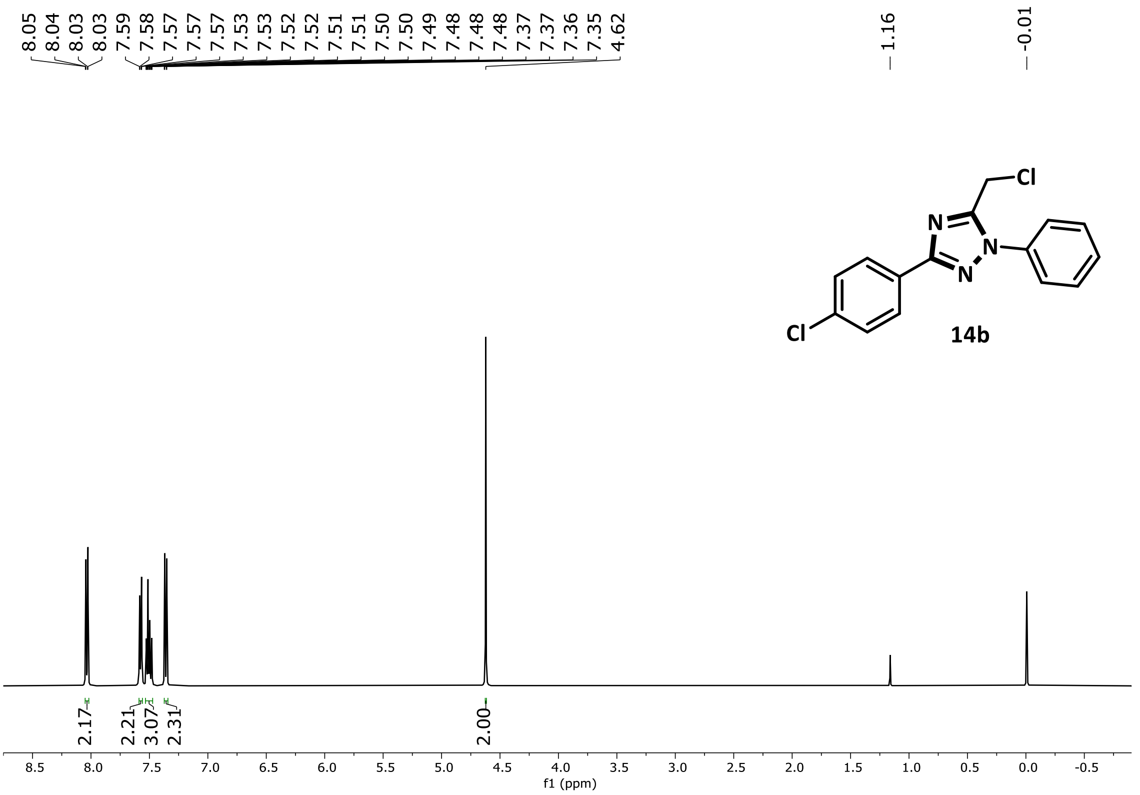

Figure S23. ^{1}H NMR spectrum of compound **14b** in $CDCl_3$ at 500 MHz.

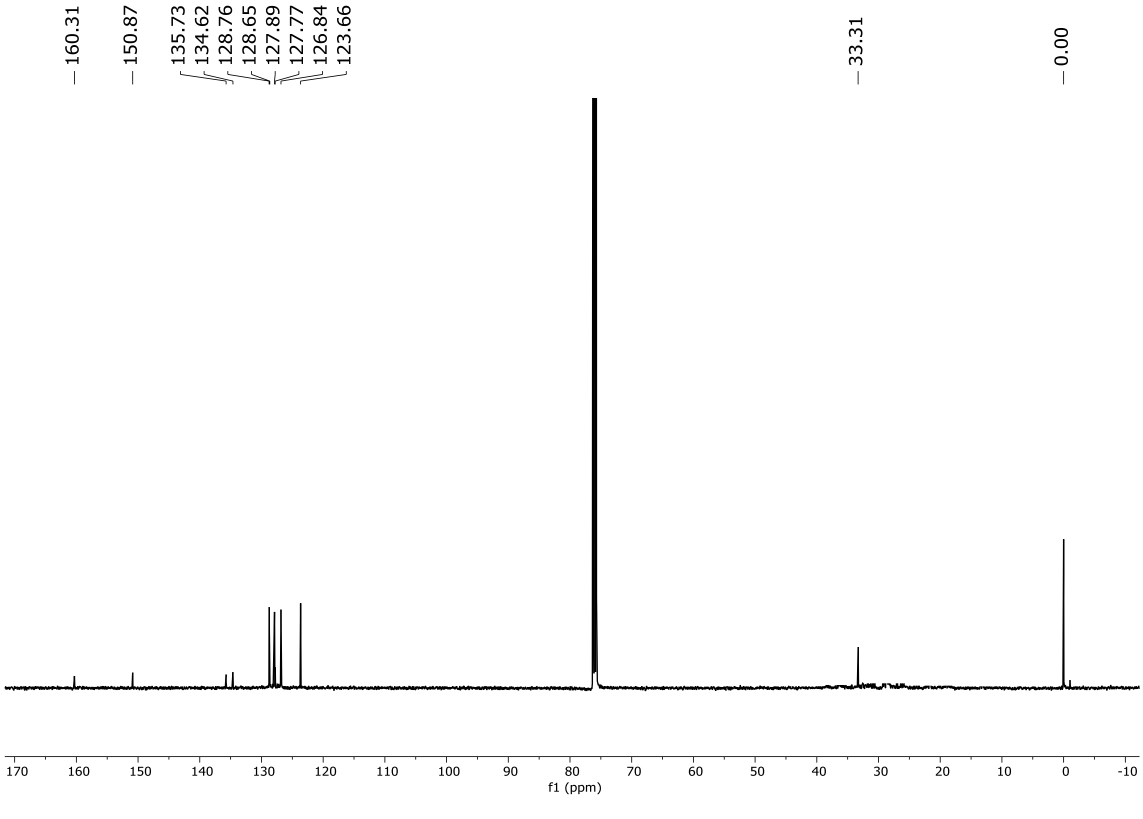

Figure S24. ^{13}C NMR spectrum of compound **14b** in $CDCl_3$ at 125 MHz.

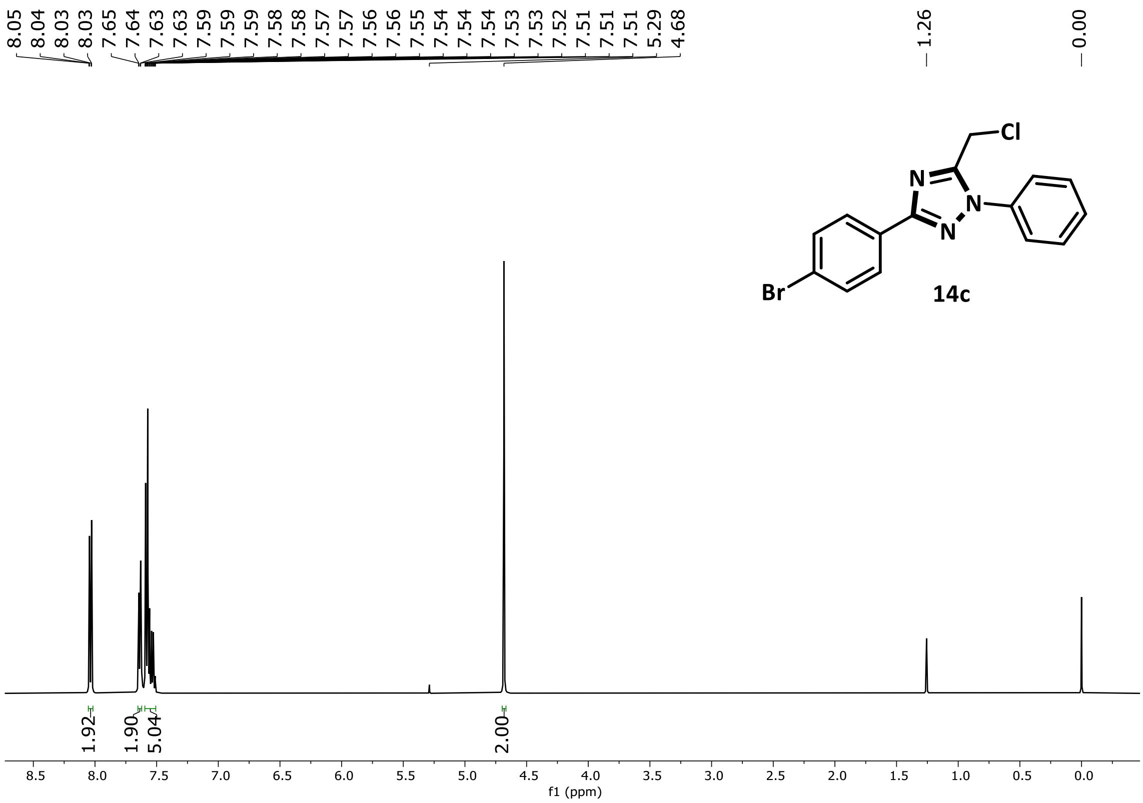

Figure S25. ^{1}H NMR spectrum of compound **14c** in $CDCl_3$ at 500 MHz.

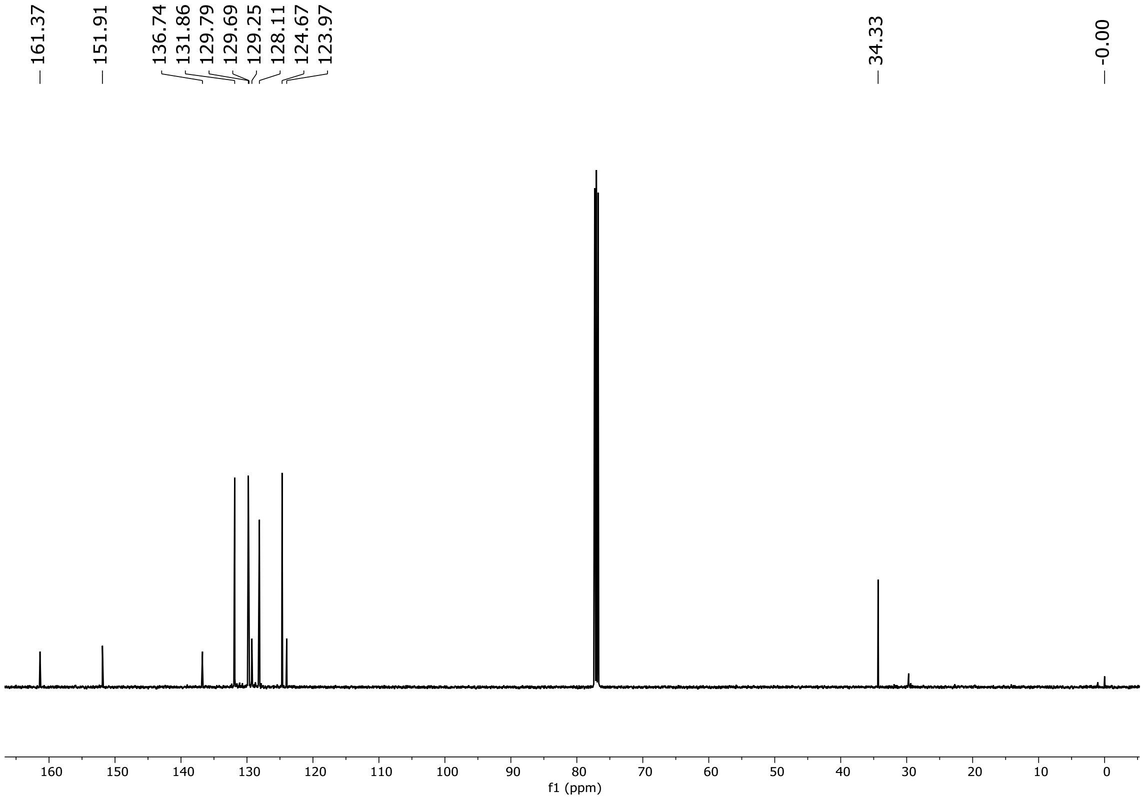

Figure S26. ^{13}C NMR spectrum of compound **14c** in $CDCl_3$ at 125 MHz.

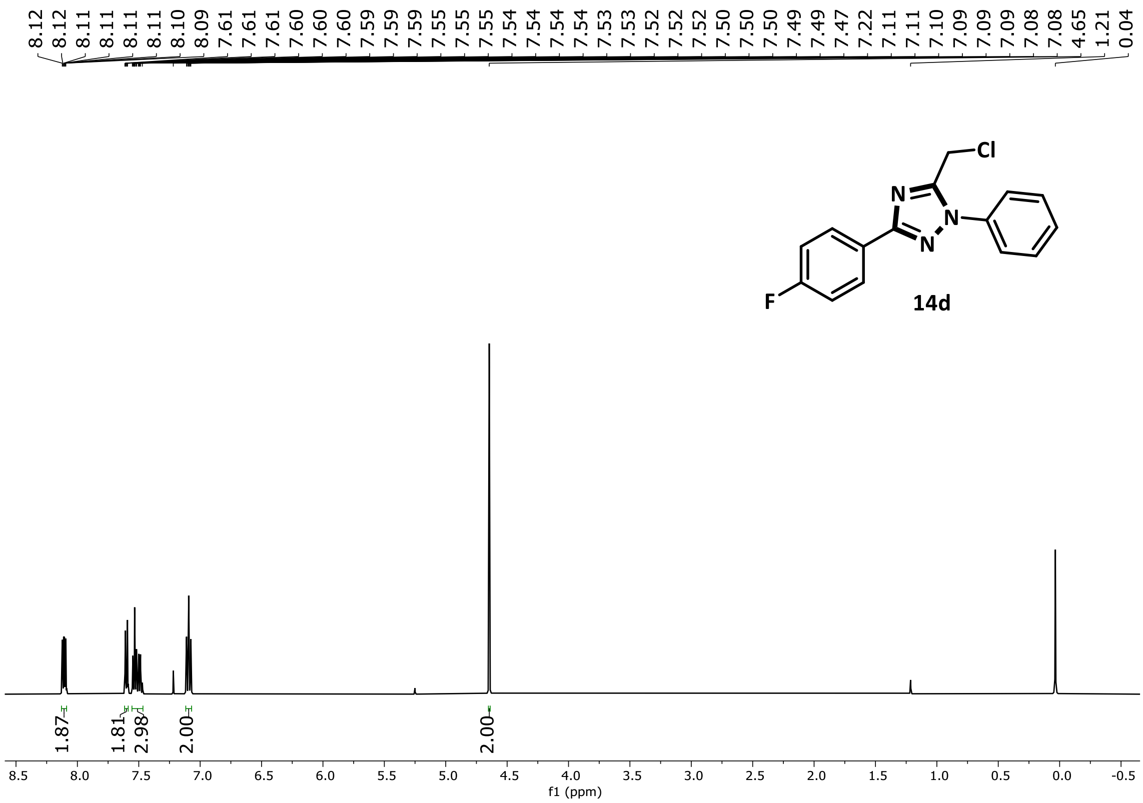

Figure S27. ^{1}H NMR spectrum of compound **14d** in $CDCl_3$ at 500 MHz.

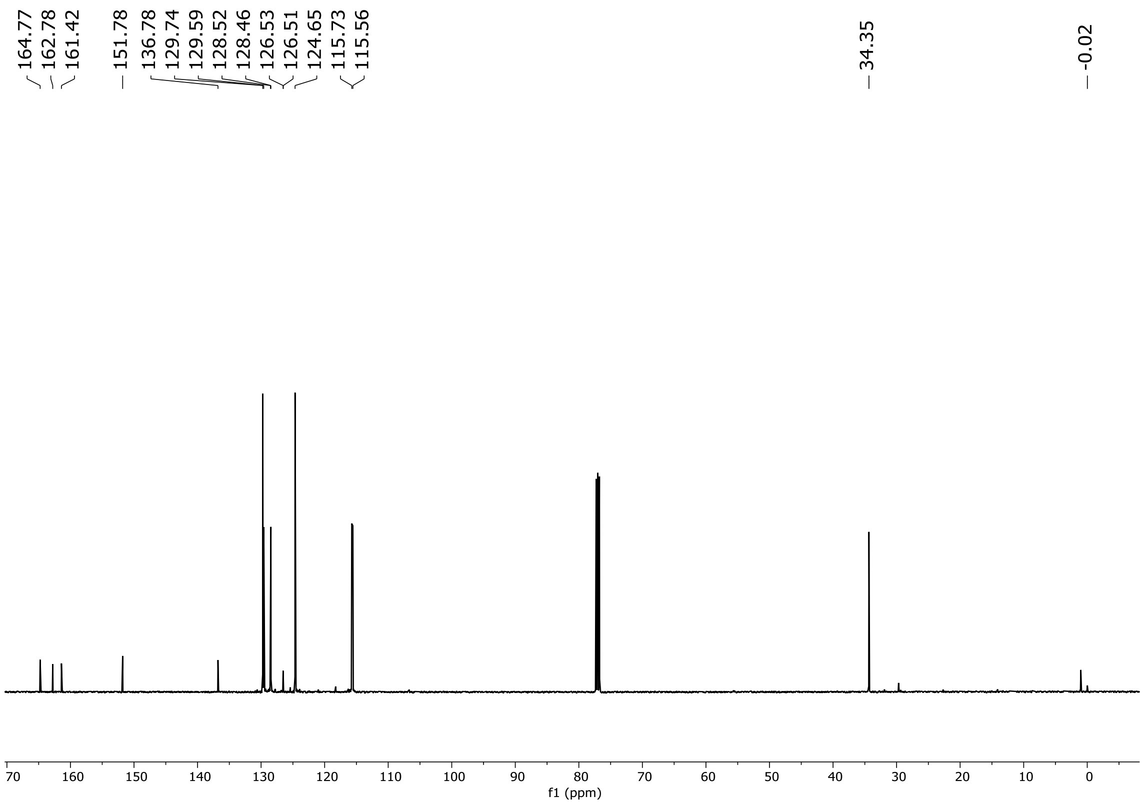

Figure S28. ^{13}C NMR spectrum of compound **14d** in $CDCl_3$ at 125 MHz.

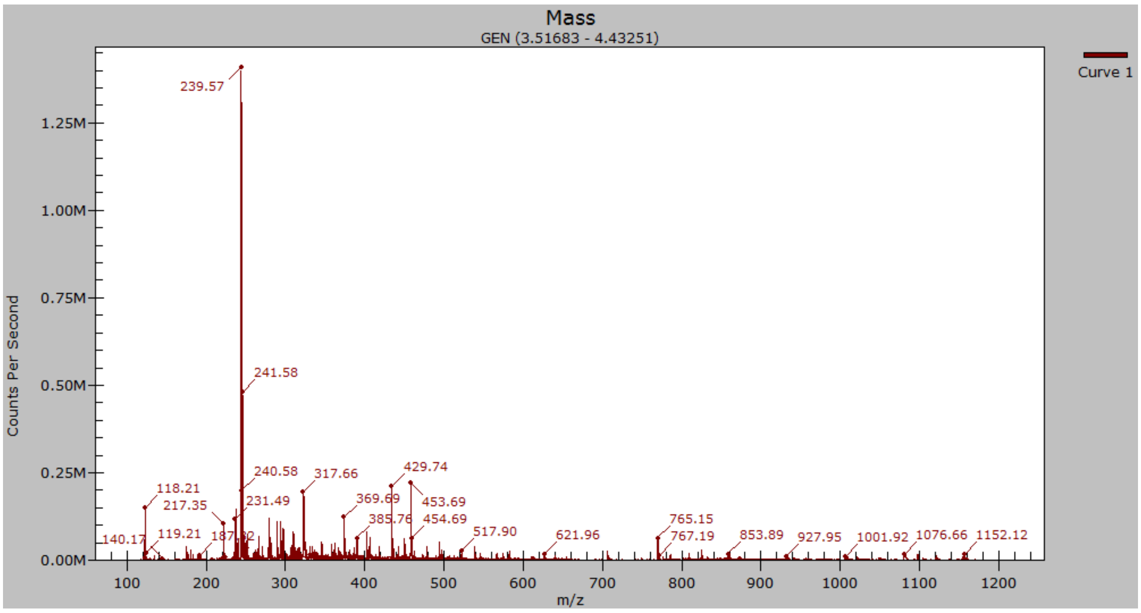

Figure S29. ESI-MS (LR) spectrum of compound **14d**.

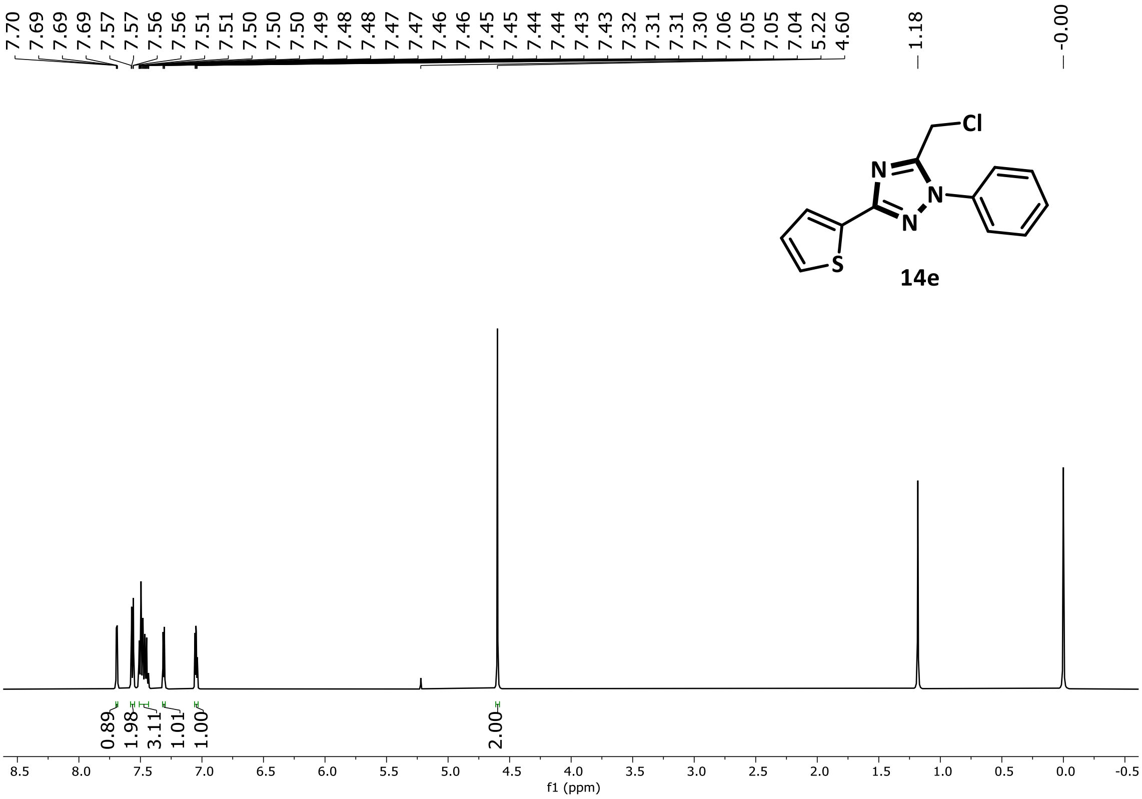

Figure S30. ^{1}H NMR spectrum of compound **14e** in $CDCl_3$ at 500 MHz.

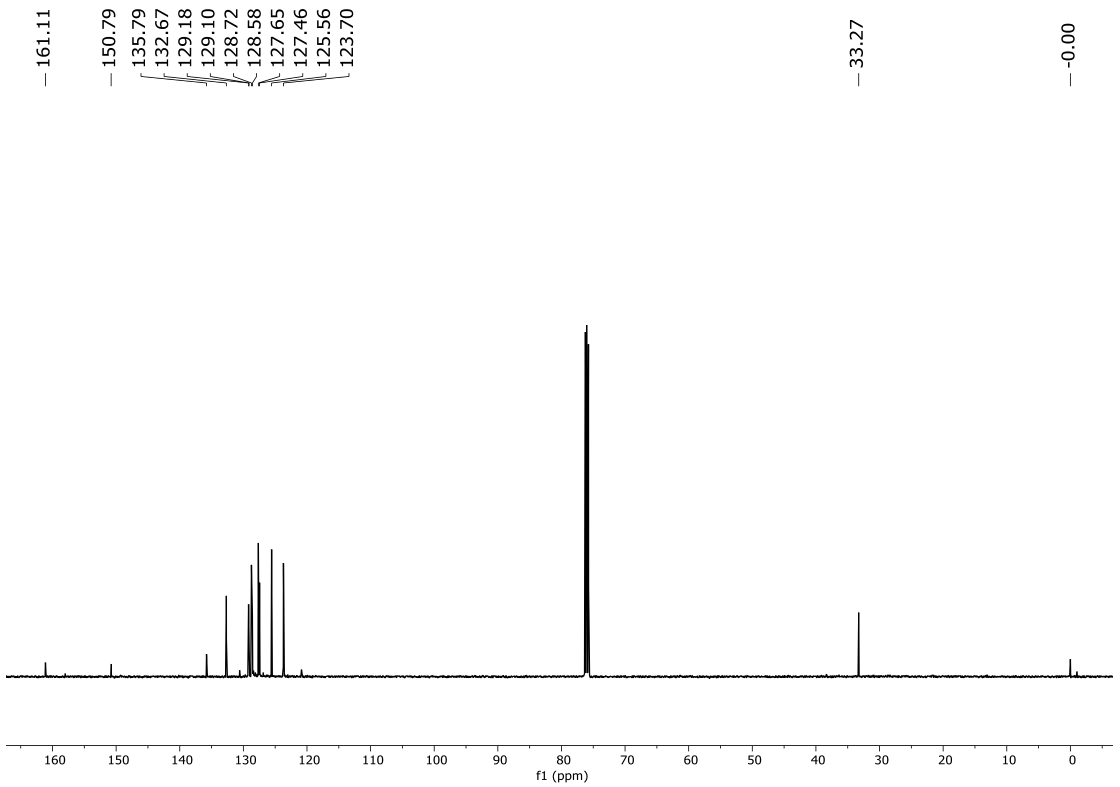

Figure S31. ^{13}C NMR spectrum of compound **14e** in $CDCl_3$ at 125 MHz.

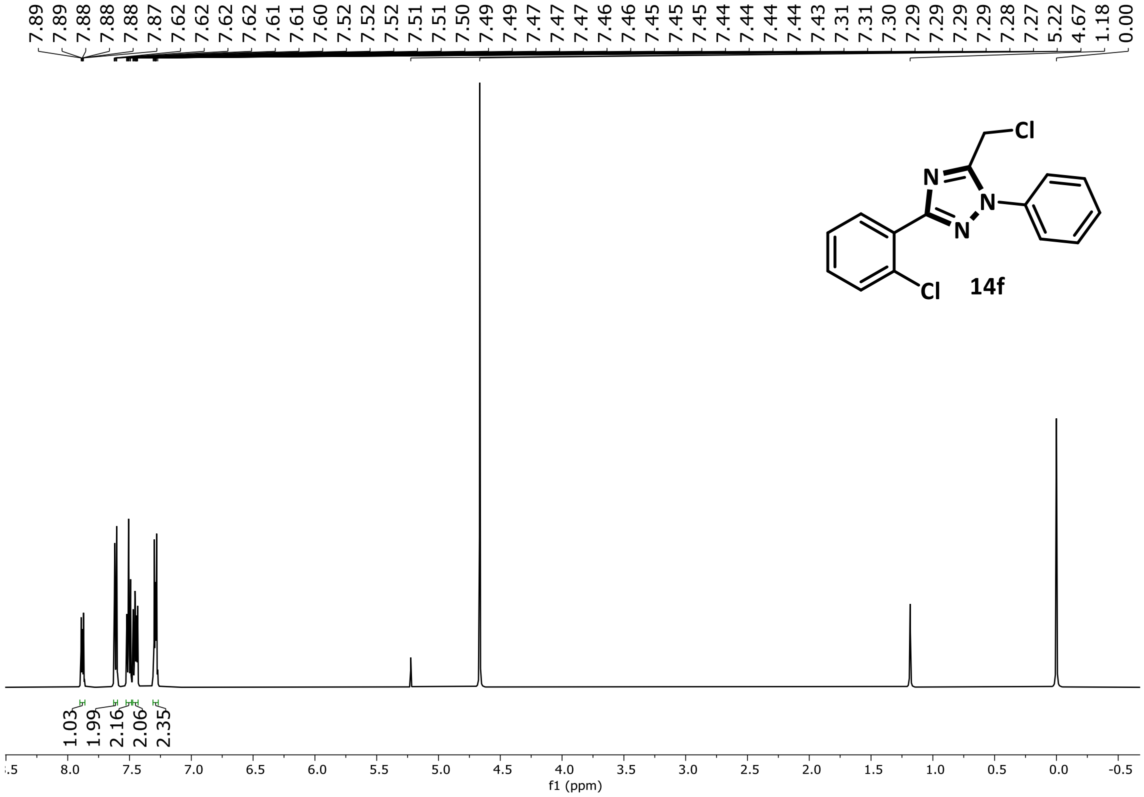

Figure S32. ^{1}H NMR spectrum of compound **14f** in $CDCl_3$ at 500 MHz.

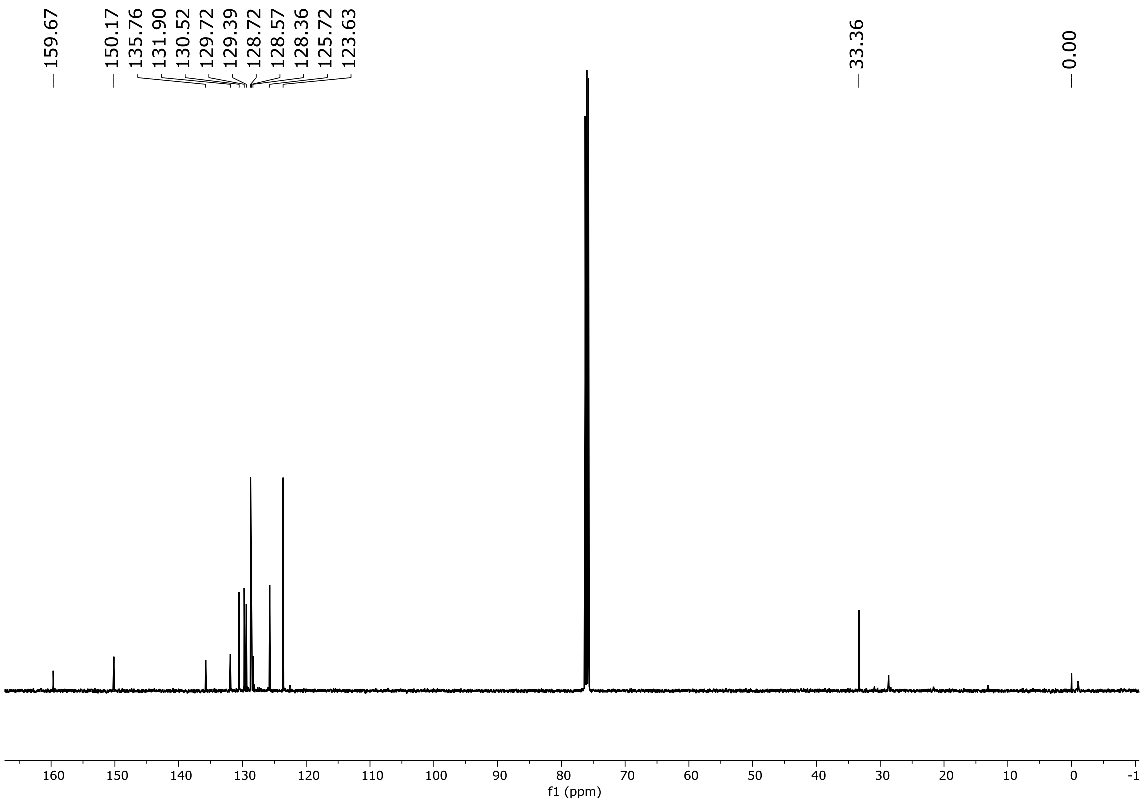

Figure S33. ^{13}C NMR spectrum of compound **14f** in $CDCl_3$ at 125 MHz.

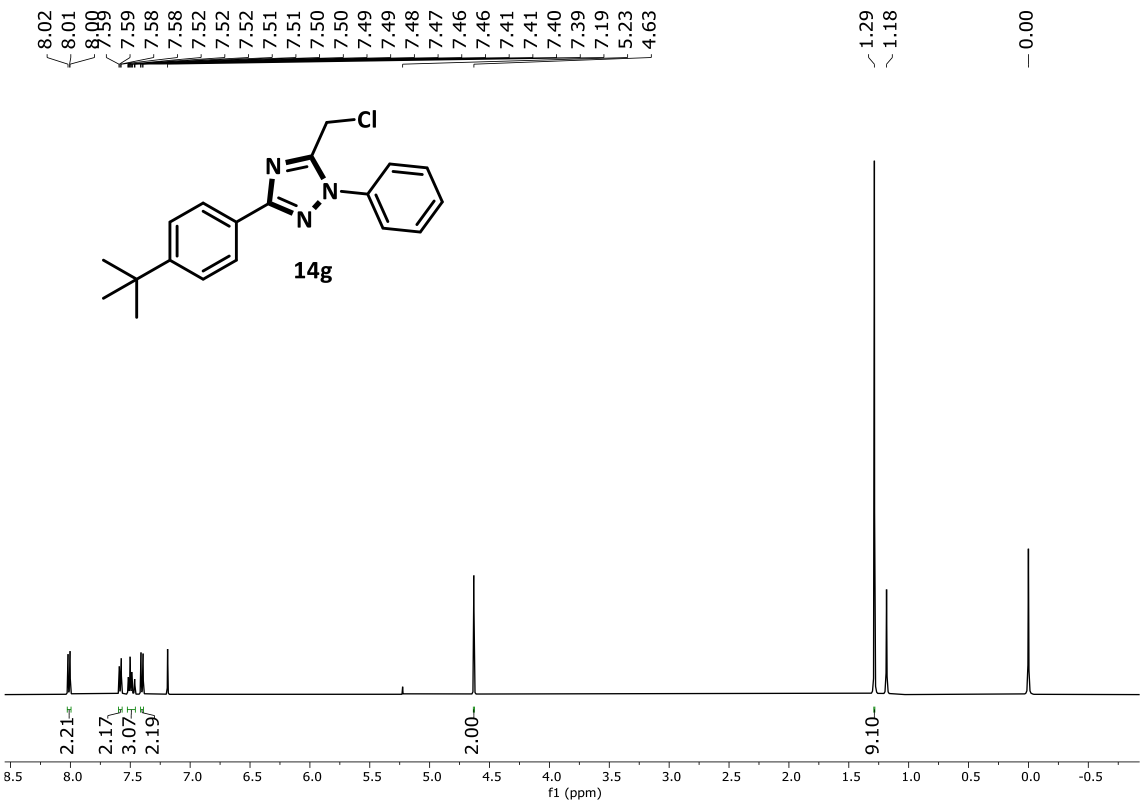

Figure S34. ^{1}H NMR spectrum of compound **14g** in $CDCl_3$ at 500 MHz.

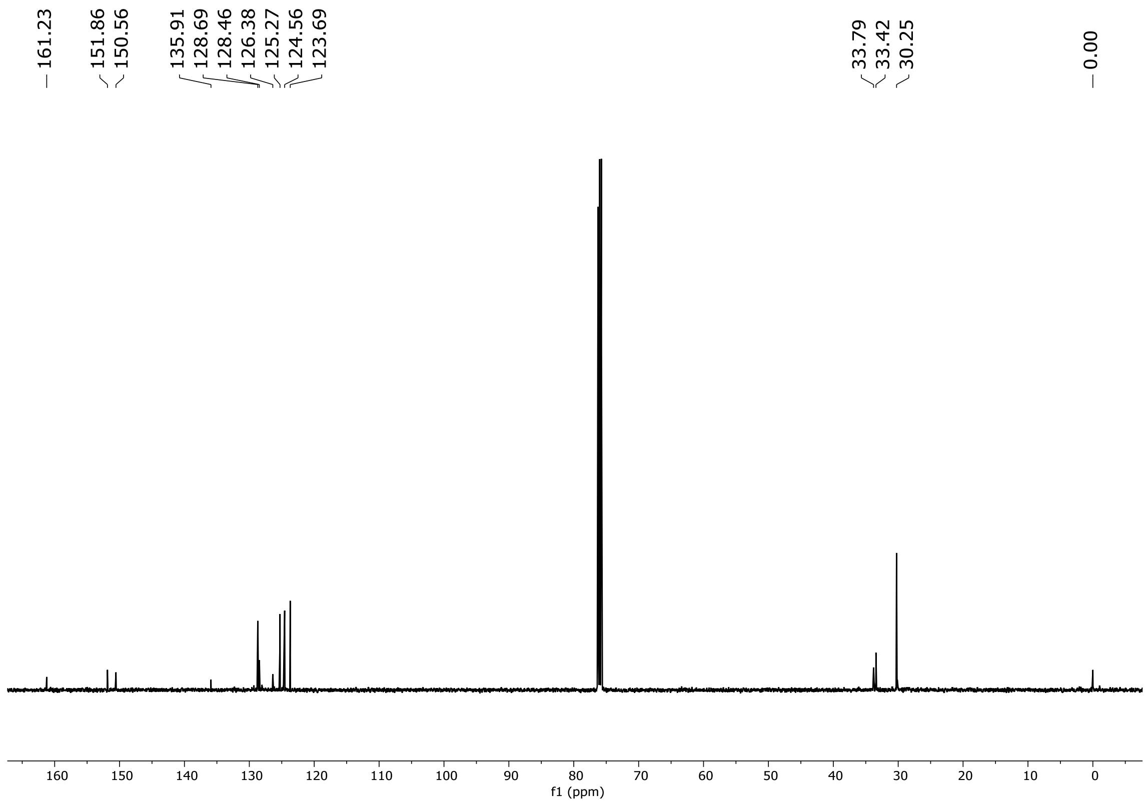

Figure S35. ^{13}C NMR spectrum of compound **14g** in $CDCl_3$ at 125 MHz.

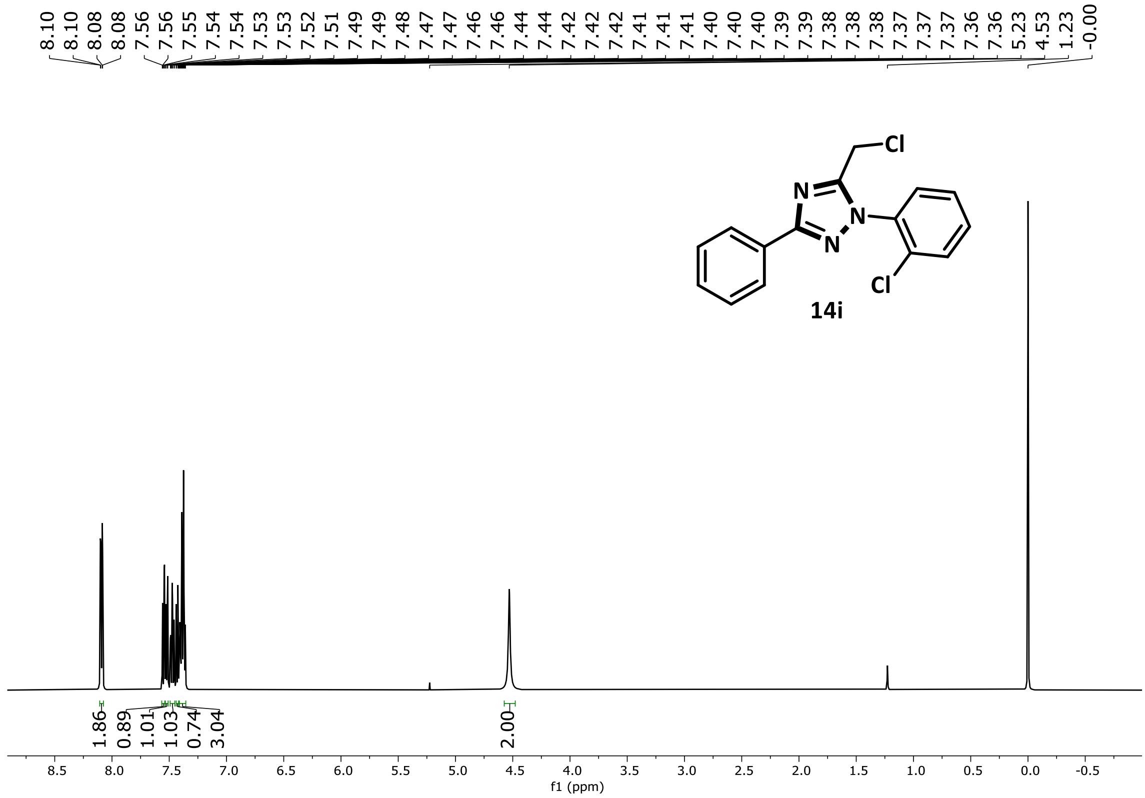

Figure S36. ^{1}H NMR spectrum of compound **14i** in $CDCl_3$ at 500 MHz.

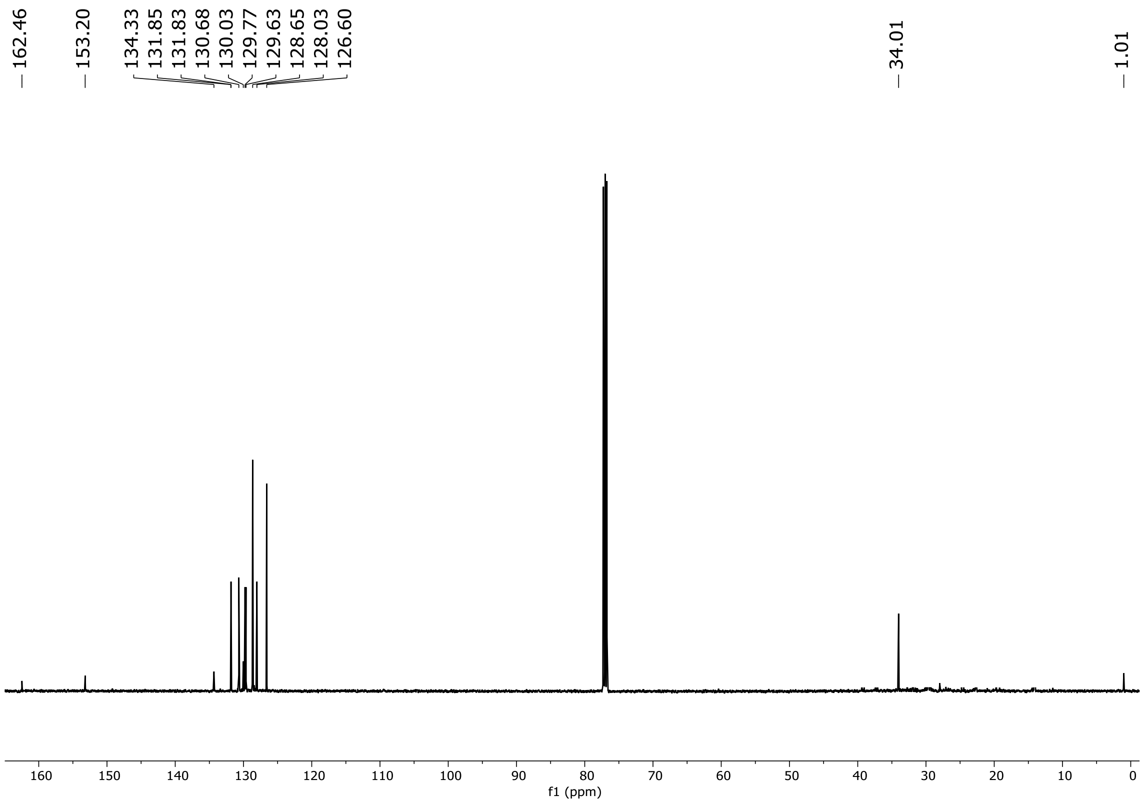

Figure S37. ^{13}C NMR spectrum of compound **14i** in $CDCl_3$ at 125 MHz.

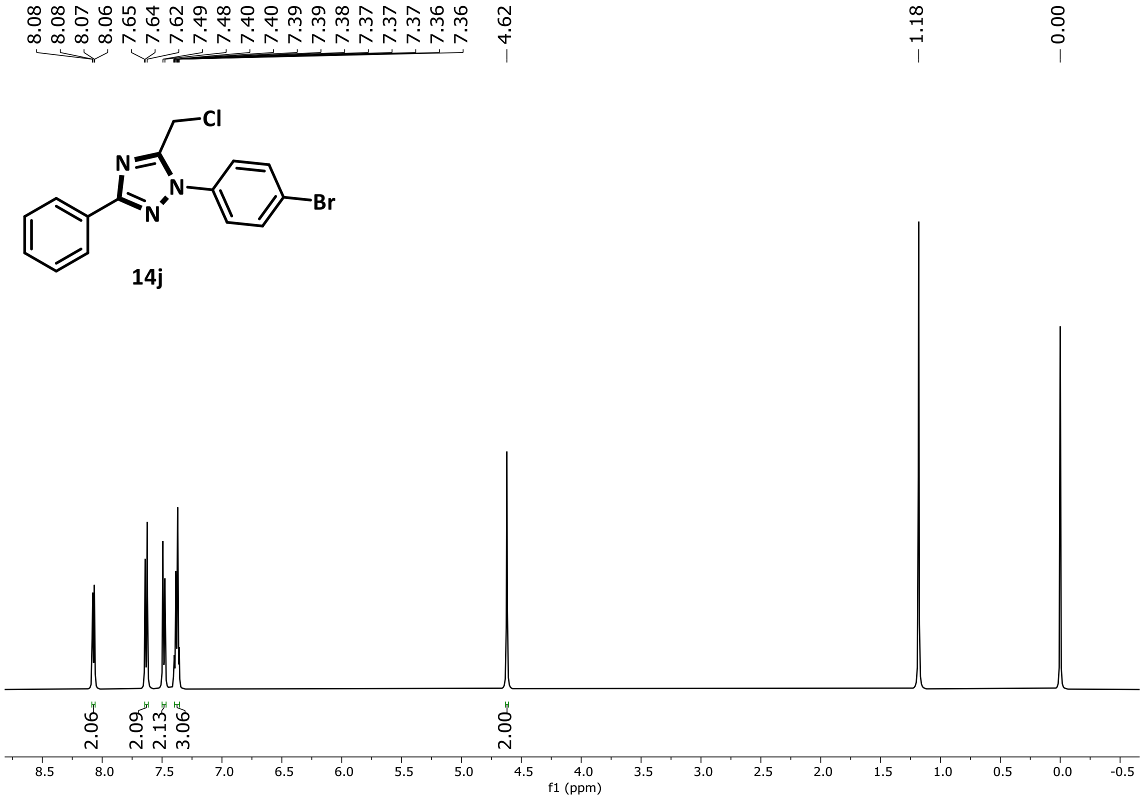

Figure S38. ^{1}H NMR spectrum of compound **14j** in $CDCl_3$ at 500 MHz.

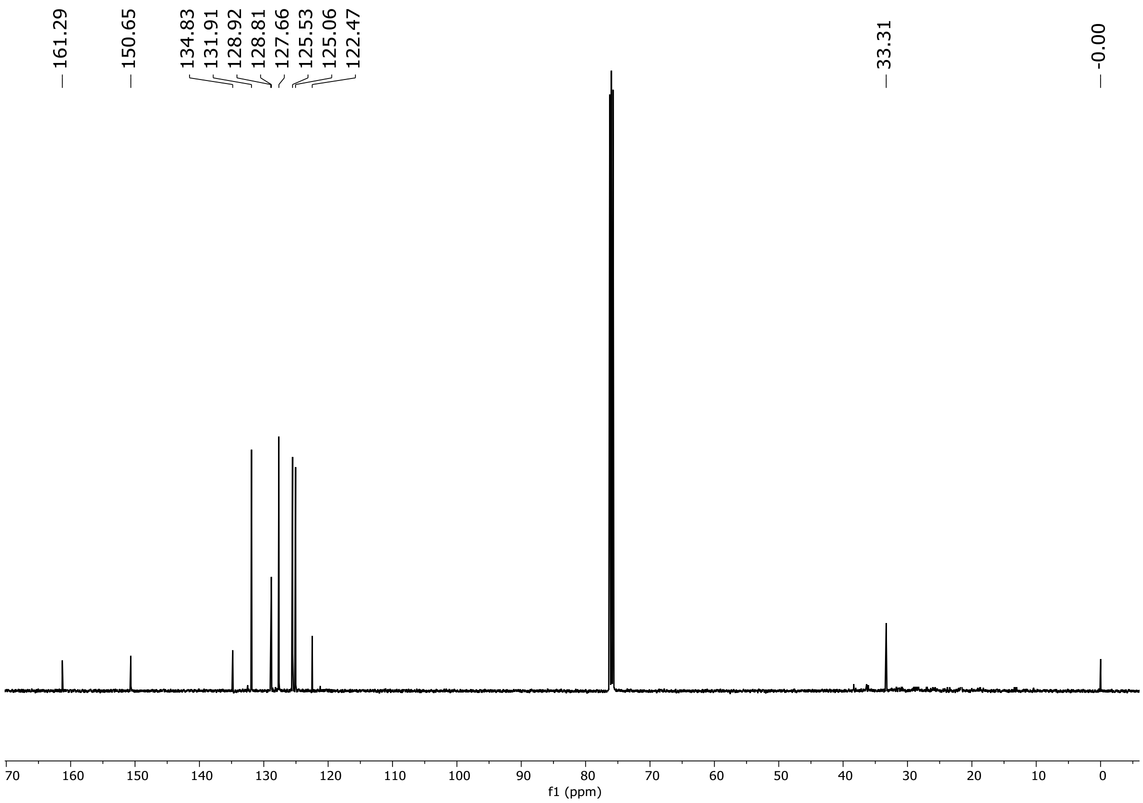

Figure S39. ^{13}C NMR spectrum of compound **14j** in $CDCl_3$ at 125 MHz.

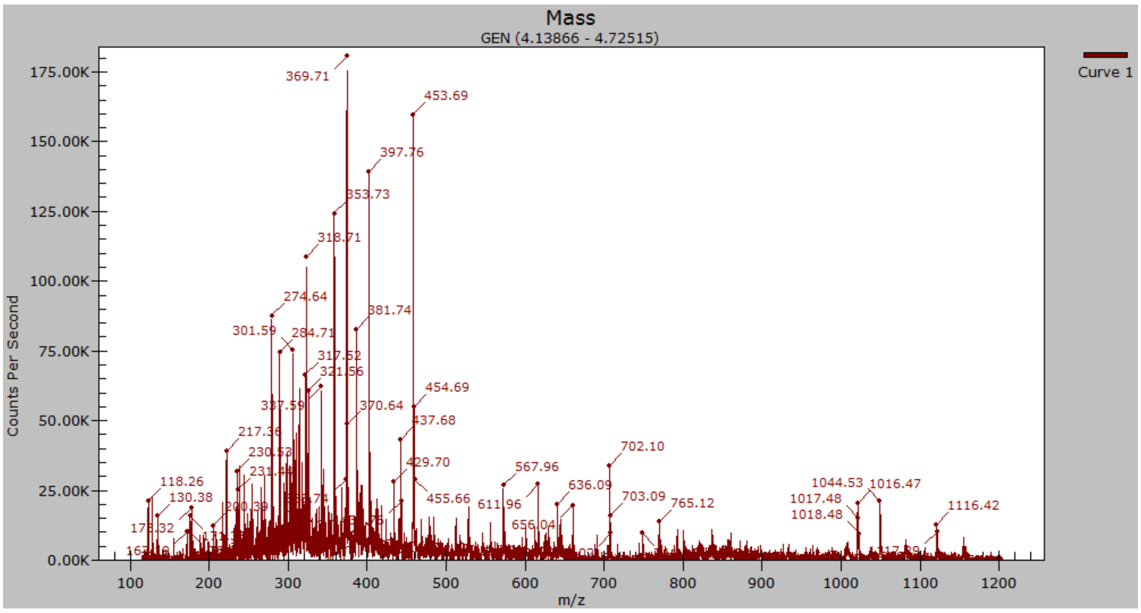

Figure S40. ESI-MS (LR) spectrum of compound **14j**.

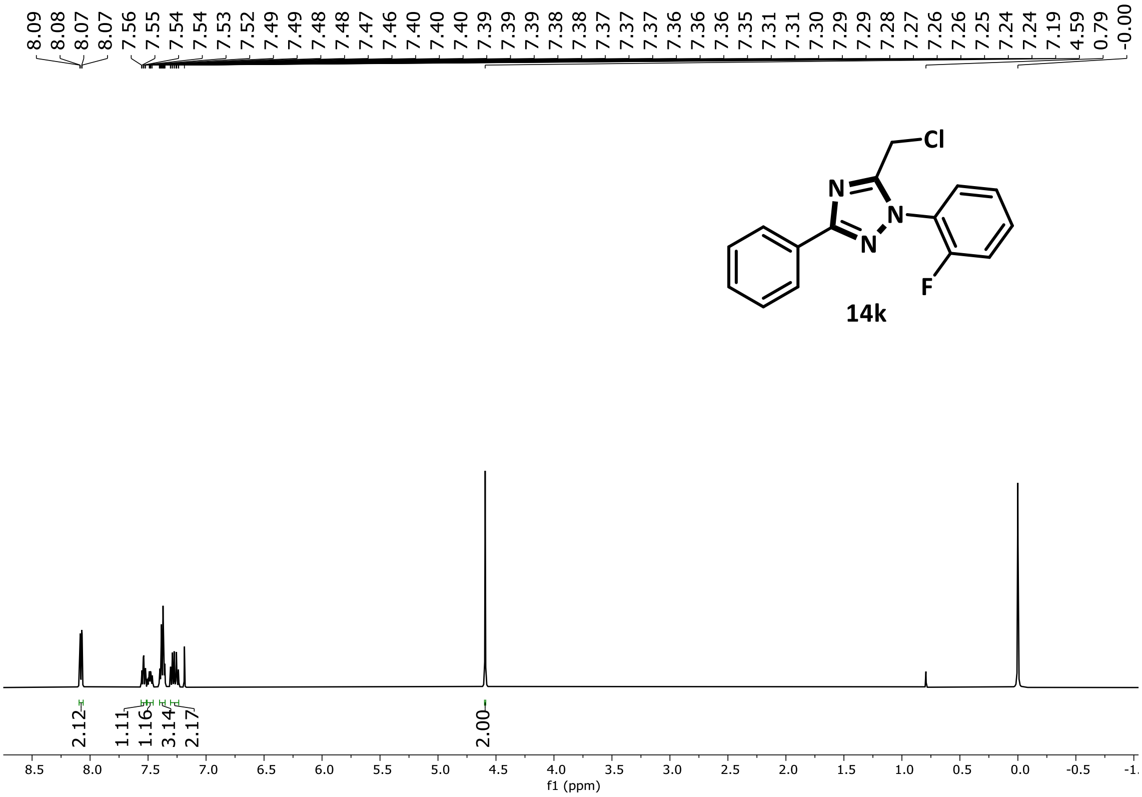

14k

Figure S41. ^{1}H NMR spectrum of compound **14k** in $CDCl_3$ at 500 MHz.

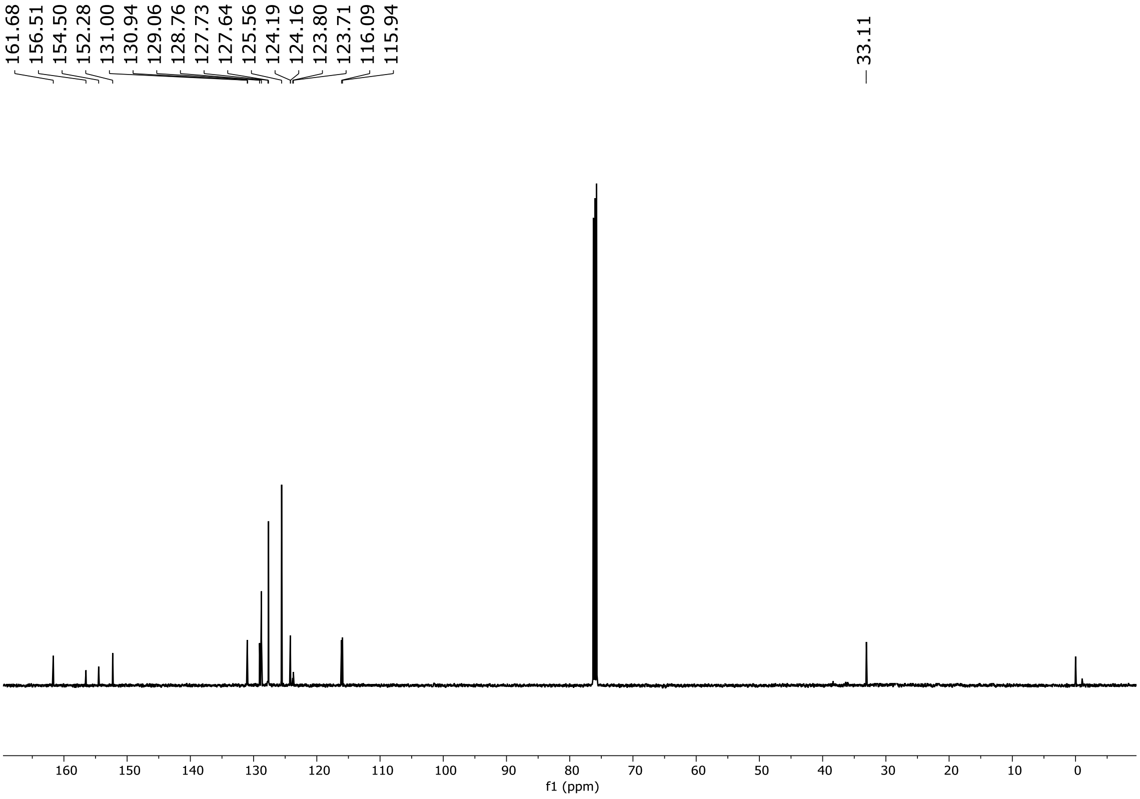

Figure S42. ^{13}C NMR spectrum of compound **14k** in $CDCl_3$ at 125 MHz.

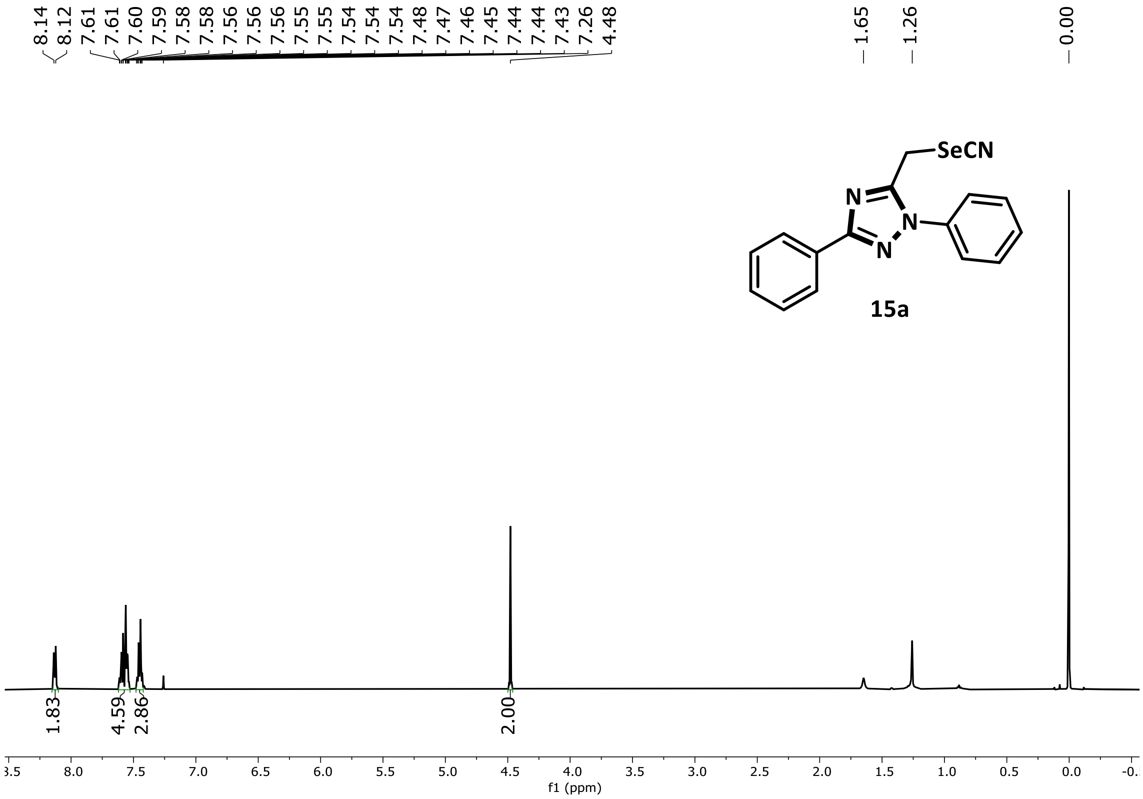

Figure S43. ^{1}H NMR spectrum of compound **15a** in $CDCl_3$ at 500 MHz.

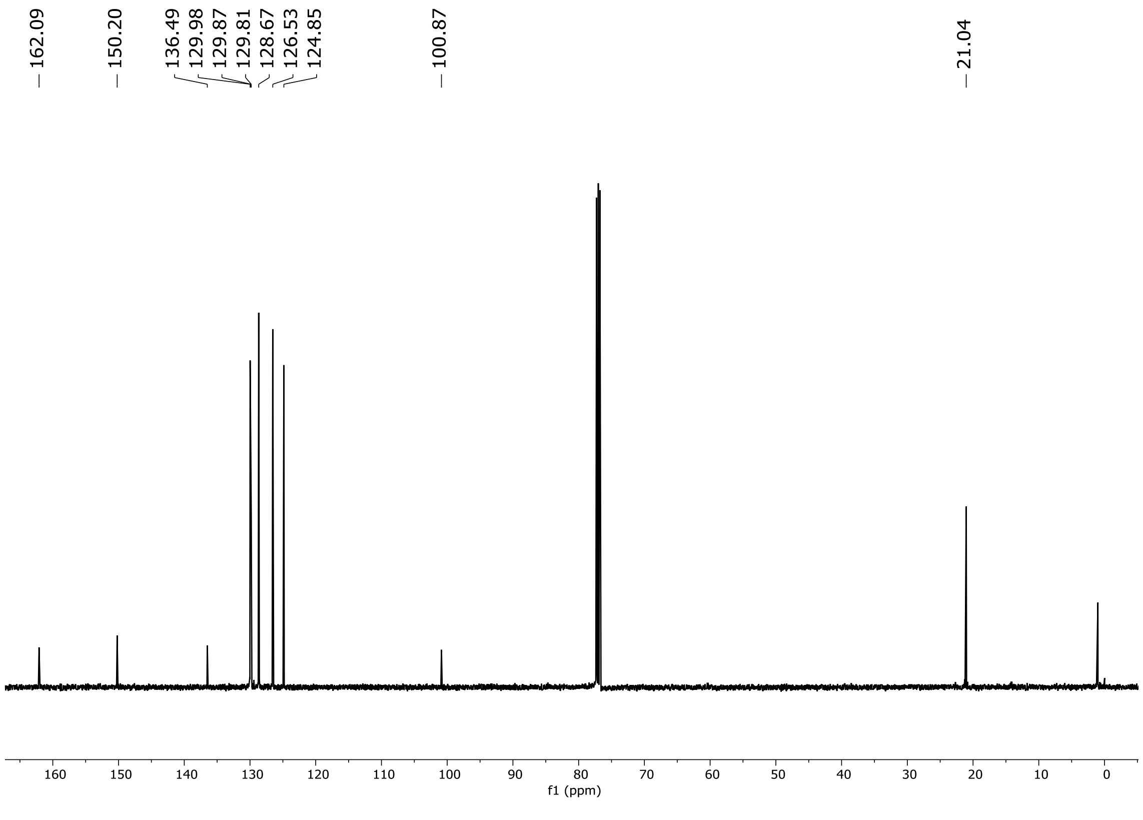

Figure S44. ^{13}C NMR spectrum of compound **15a** in $CDCl_3$ at 125 MHz.

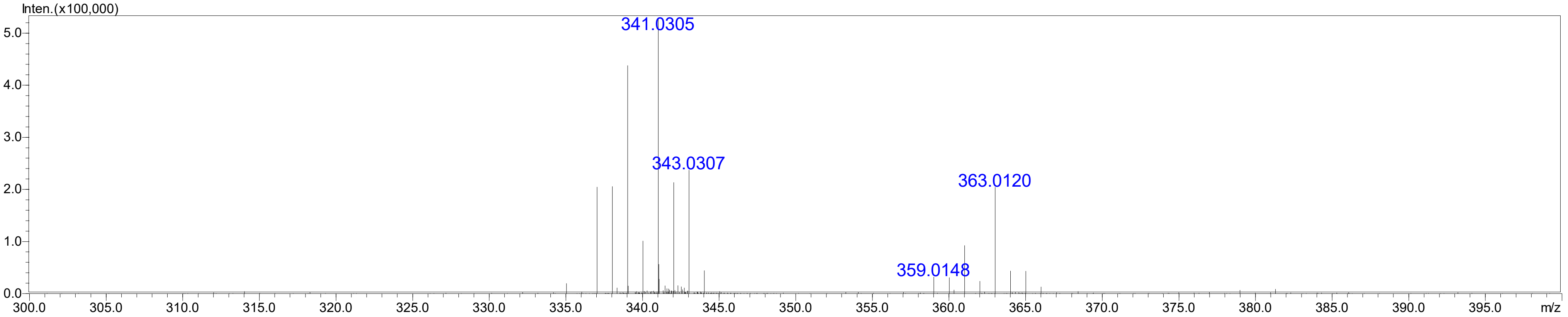

Figure S45. ESI MS spectrum of **15a.**

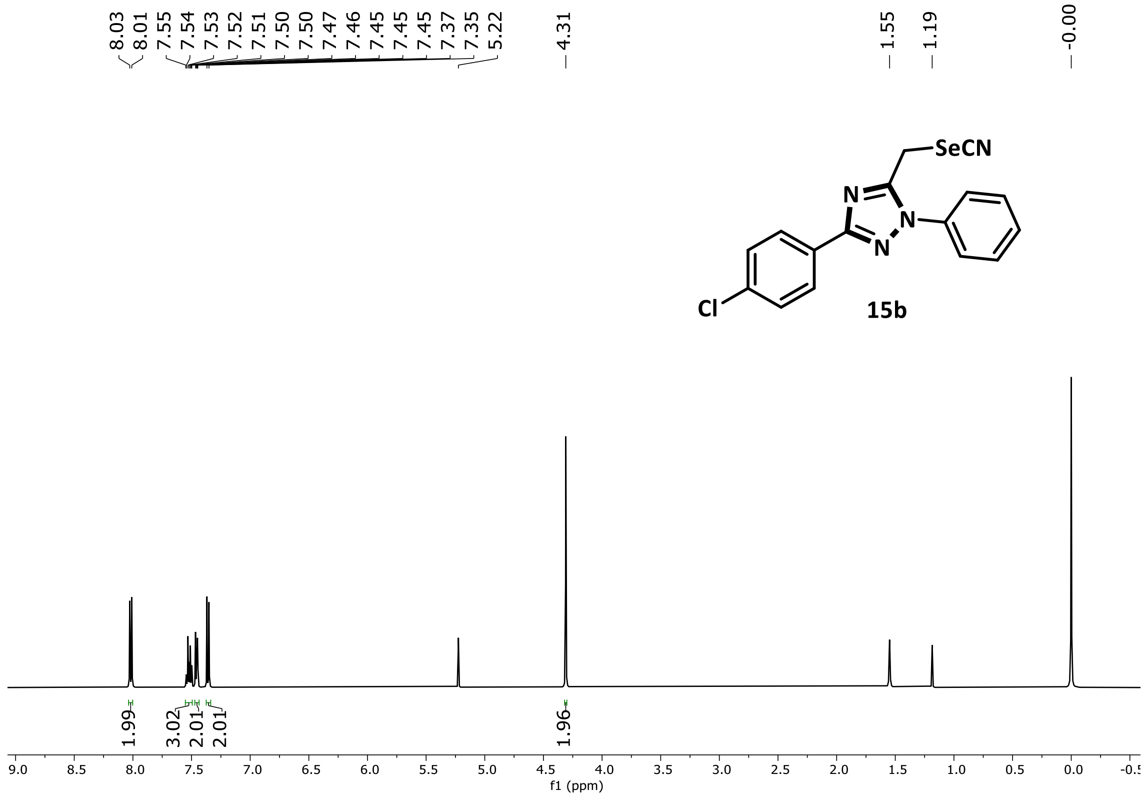

Figure S46. ^{1}H NMR spectrum of compound **15b** in $CDCl_3$ at 500 MHz.

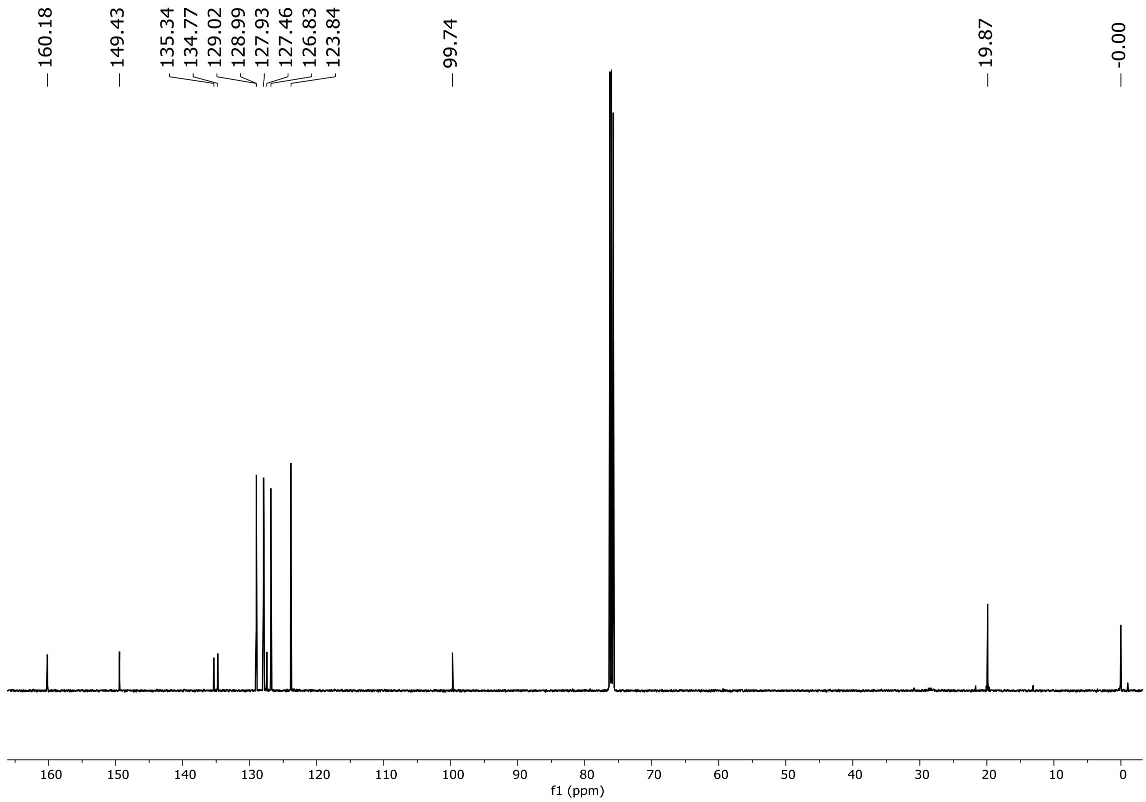

Figure S47. ^{13}C NMR spectrum of compound **15b** in $CDCl_3$ at 125 MHz.

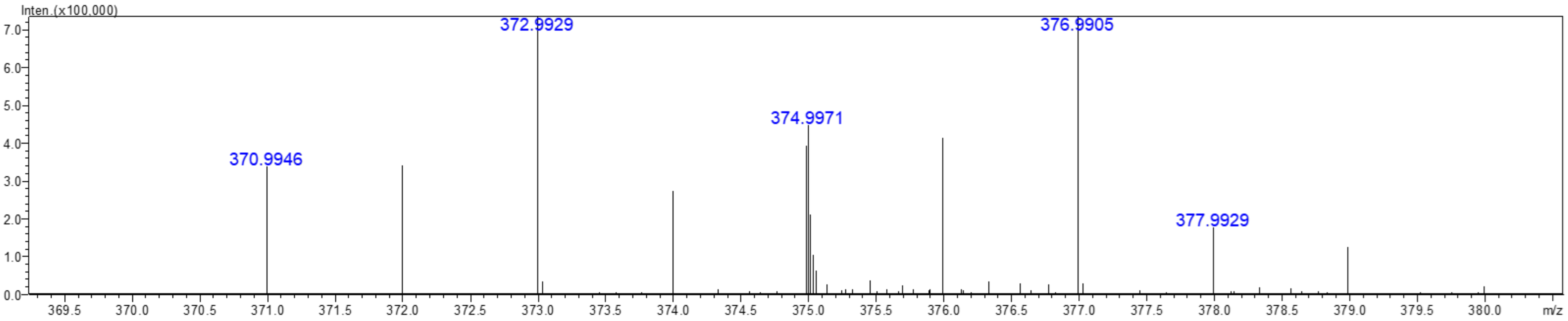

Figure S48. ESI MS spectrum of **15b**.

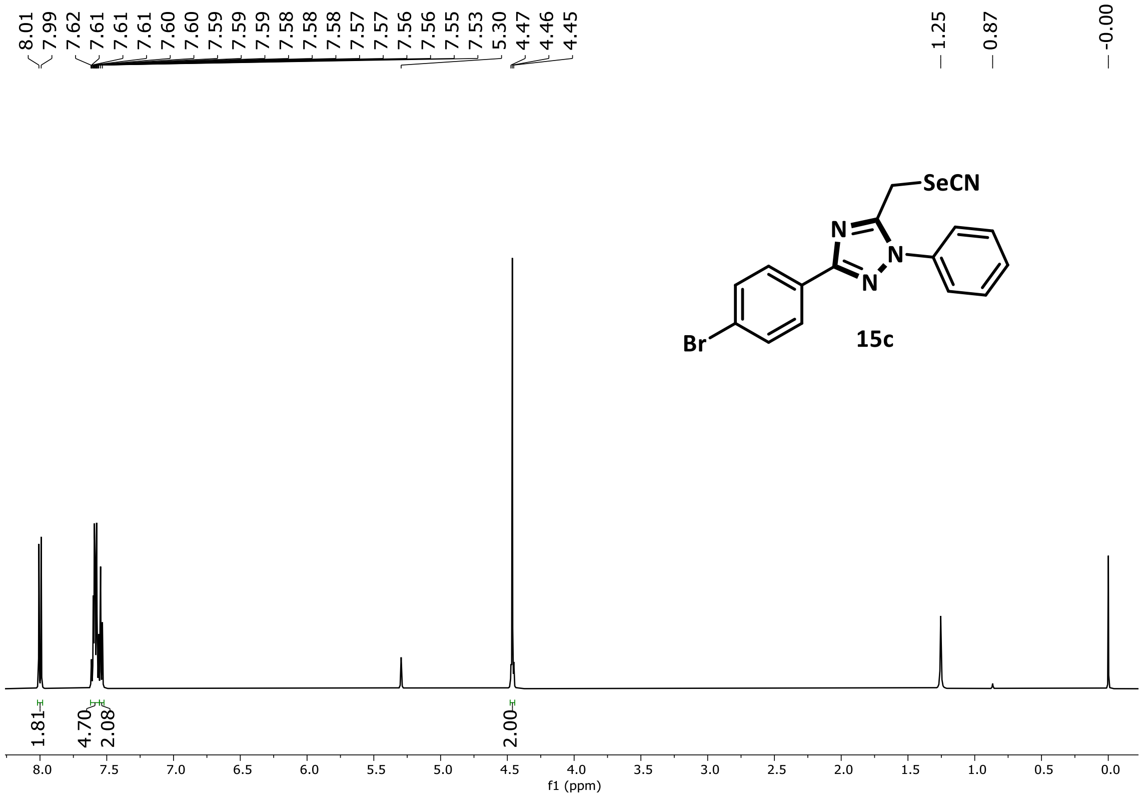

Figure S49. ^{1}H NMR spectrum of compound **15c** in $CDCl_3$ at 500 MHz.

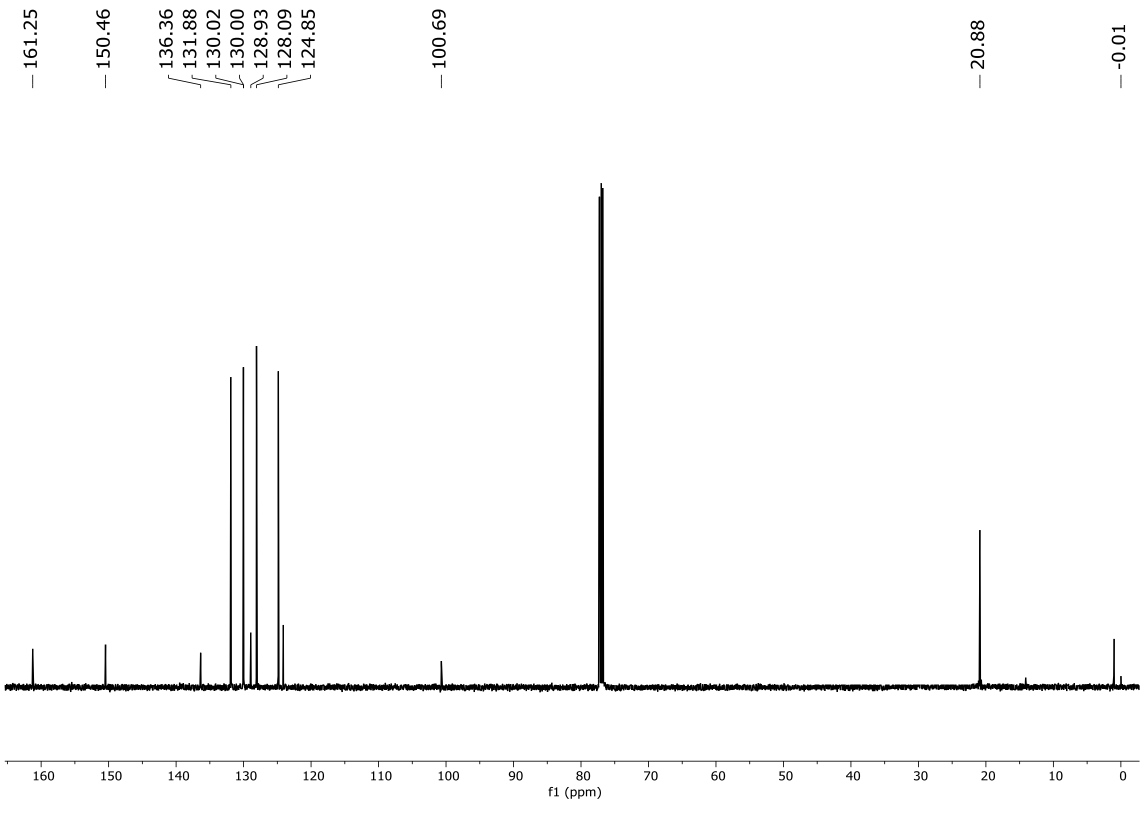

Figure S50. ^{13}C NMR spectrum of compound **15c** in $CDCl_3$ at 125 MHz.

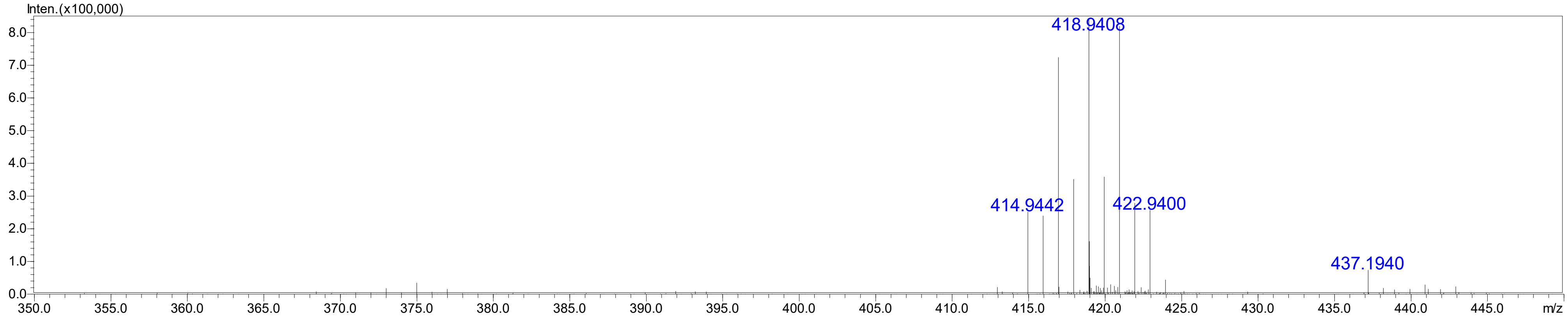

Figure S51. ESI MS spectrum of **15c**.

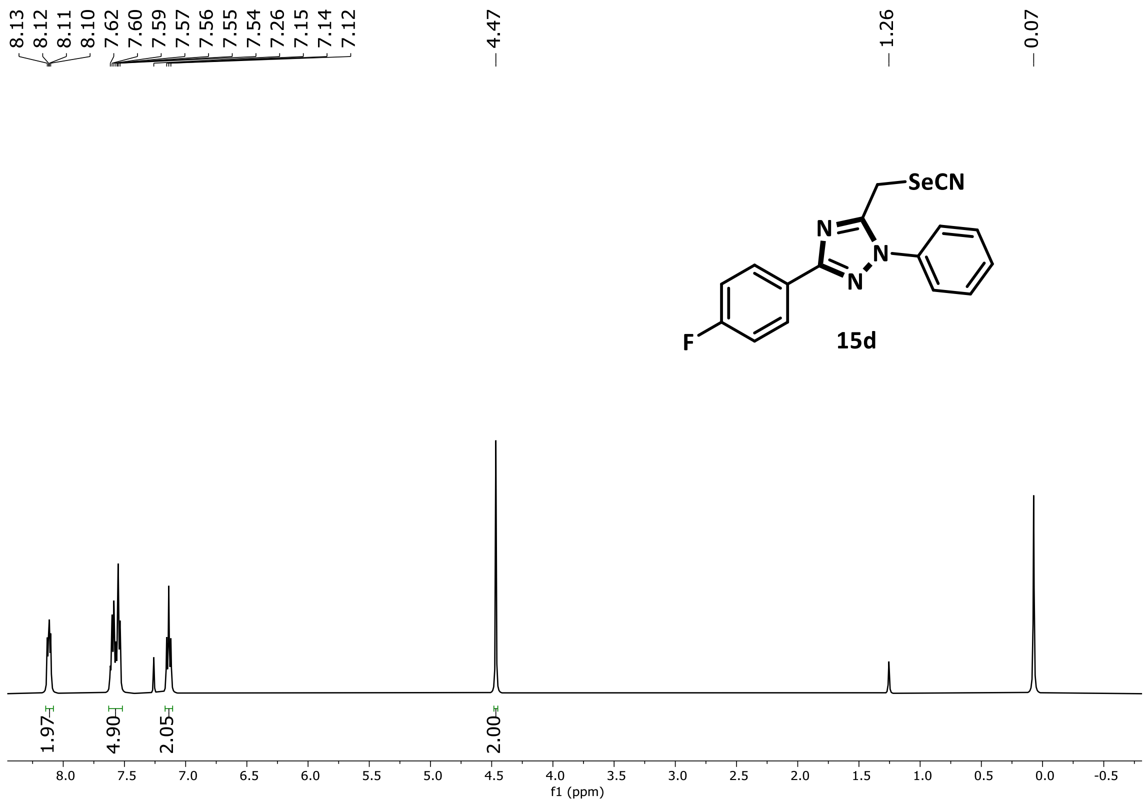

Figure S52. ^{1}H NMR spectrum of compound **15d** in $CDCl_3$ at 500 MHz.

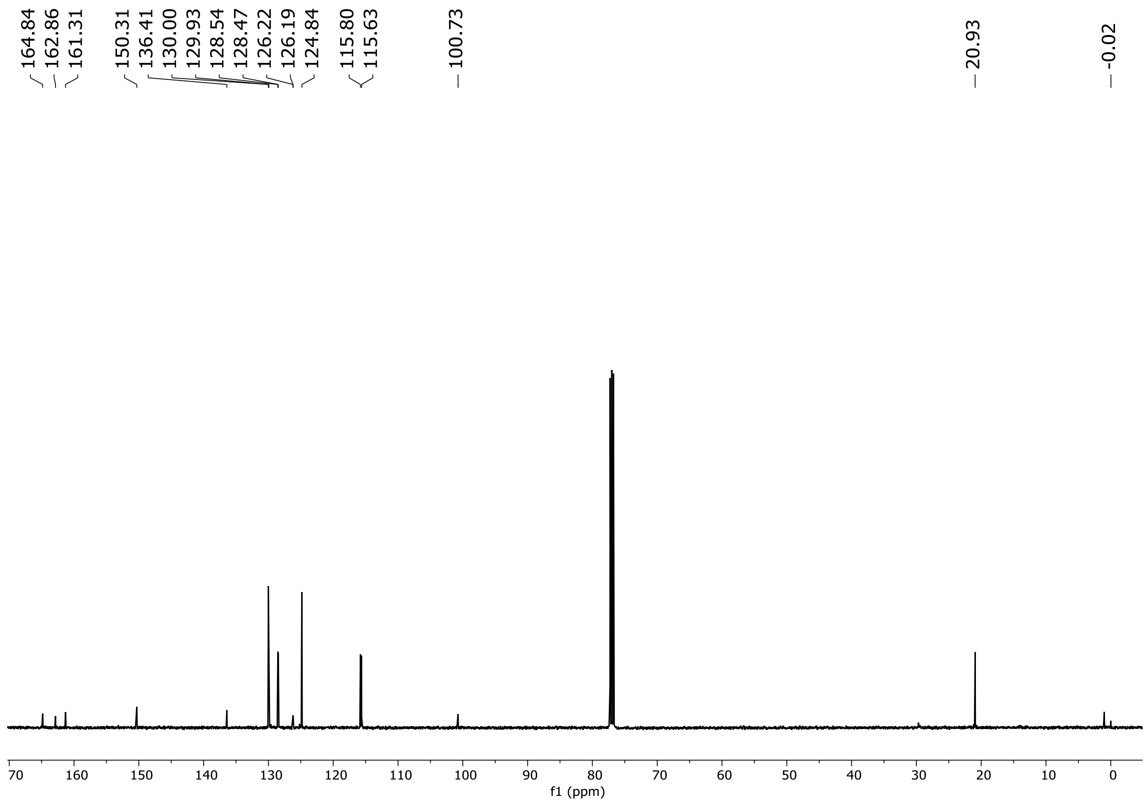

Figure S53. ^{13}C NMR spectrum of compound **15d** in $CDCl_3$ at 125 MHz.

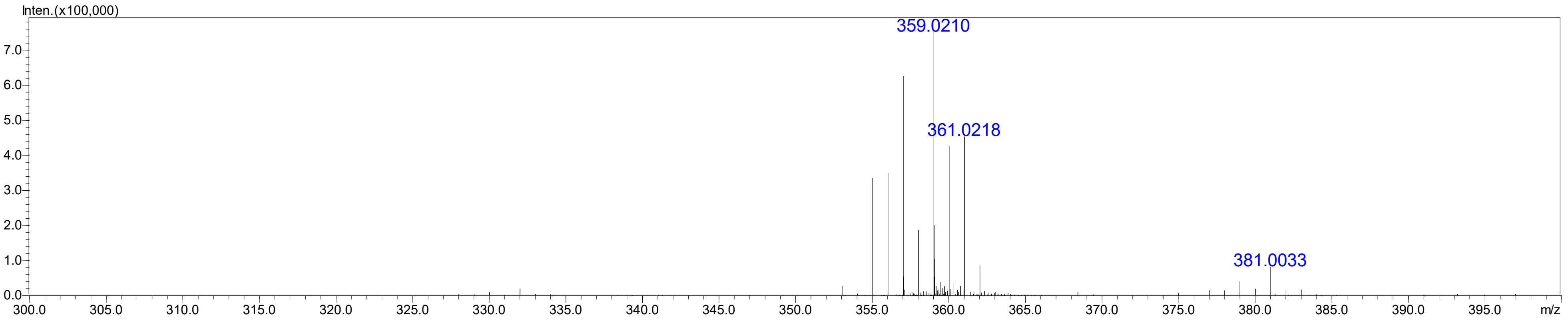

Figure S54. ESI MS spectrum of **15d**.

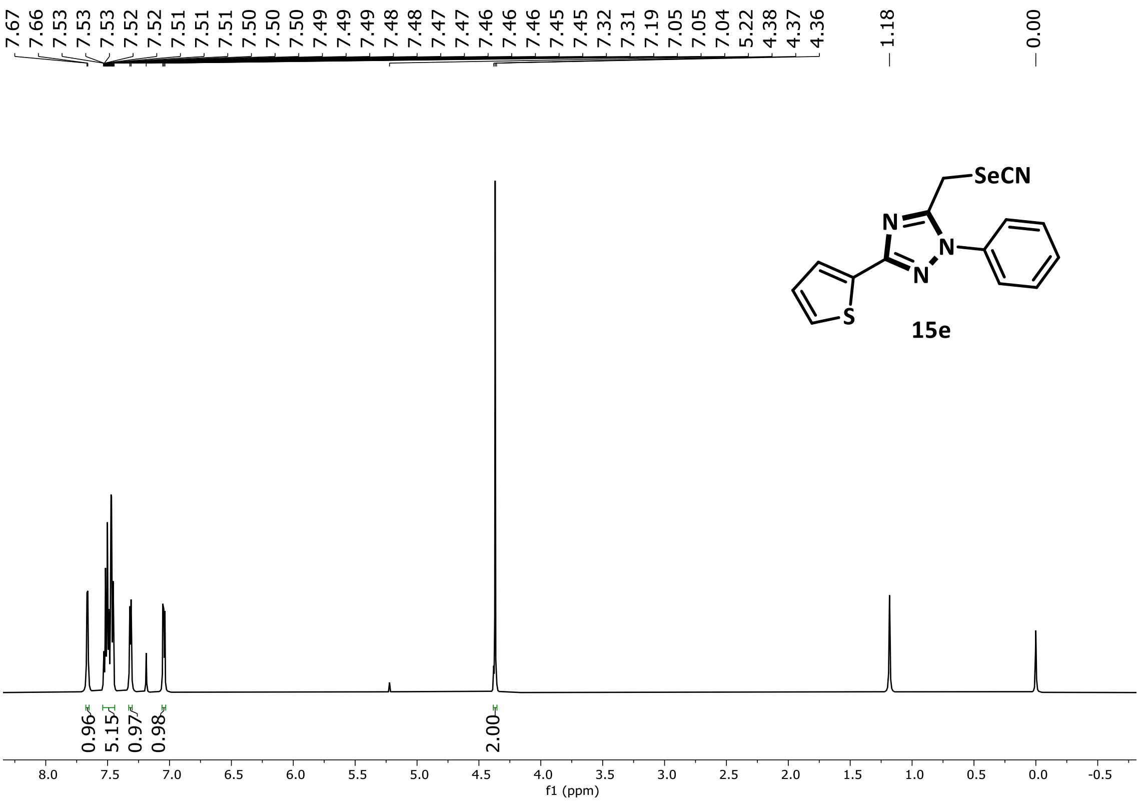

Figure S55. ^{1}H NMR spectrum of compound **15e** in $CDCl_3$ at 500 MHz.

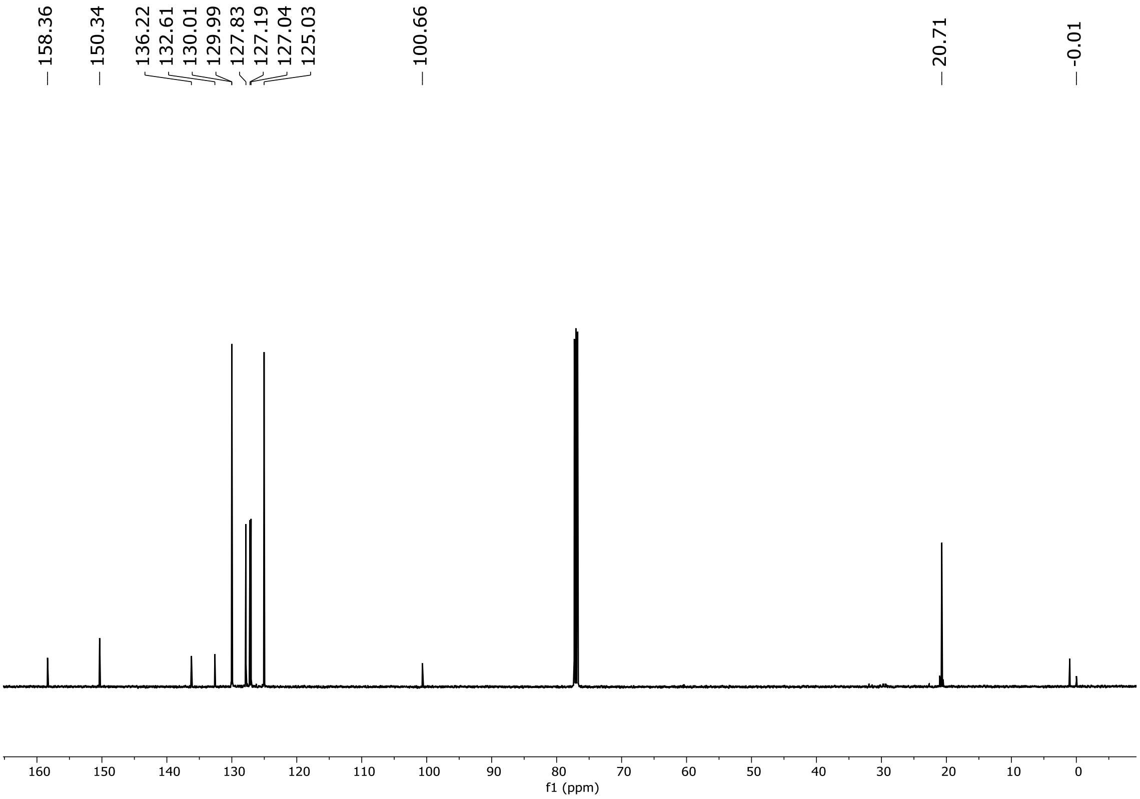

Figure S56. ^{13}C NMR spectrum of compound **15e** in $CDCl_3$ at 125 MHz.

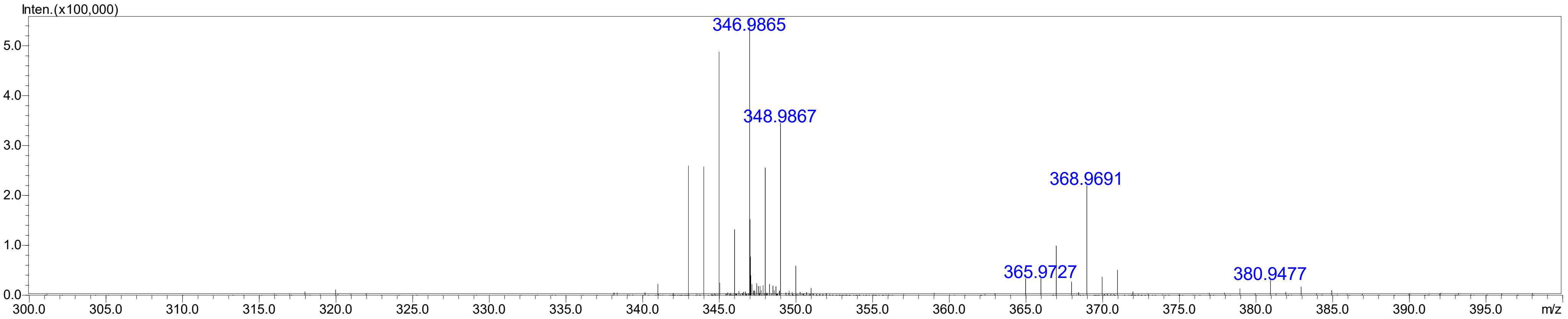

Figure S57. ESI MS spectrum of **15e**.

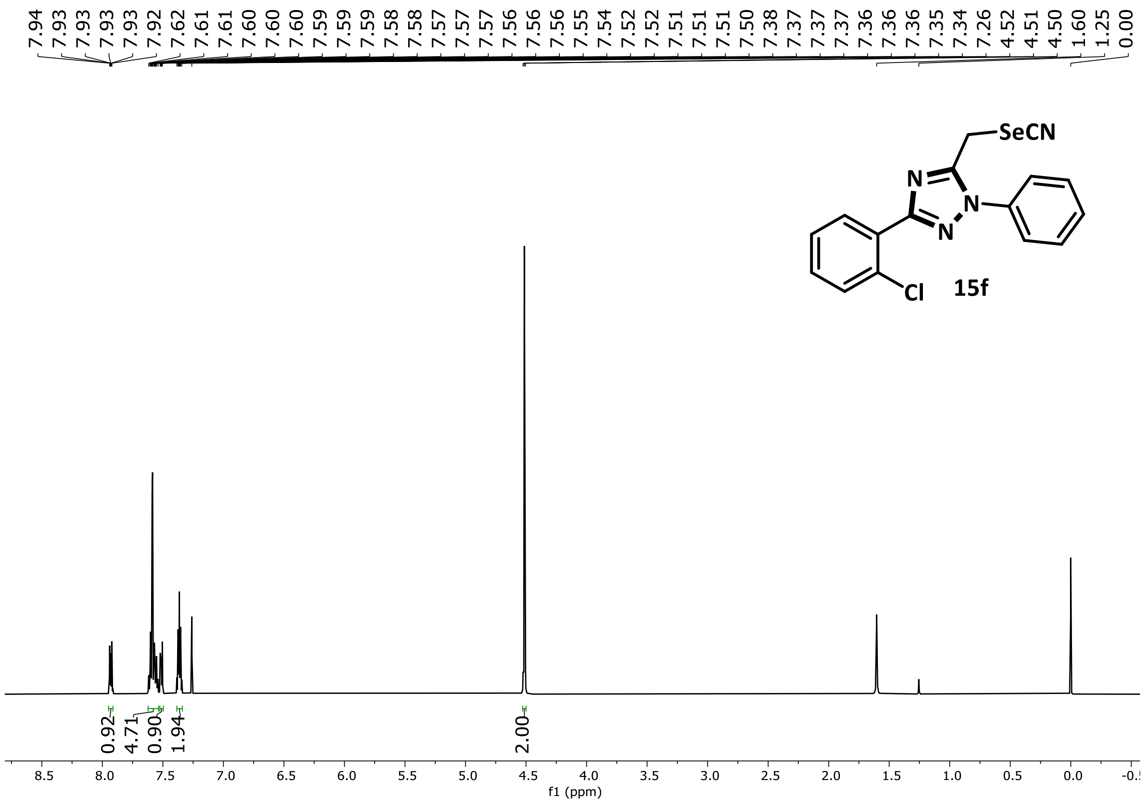

Figure S58. ^{1}H NMR spectrum of compound **15f** in $CDCl_3$ at 500 MHz.

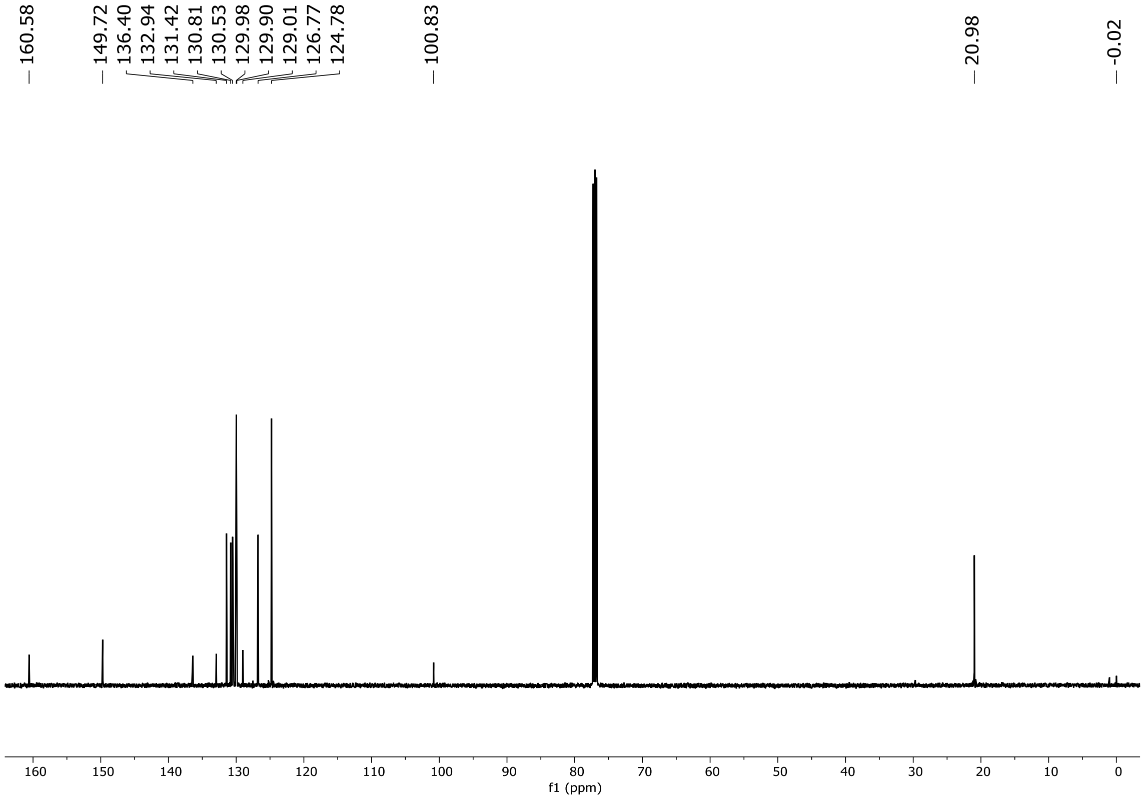

Figure S59. ^{13}C NMR spectrum of compound **15f** in $CDCl_3$ at 125 MHz.

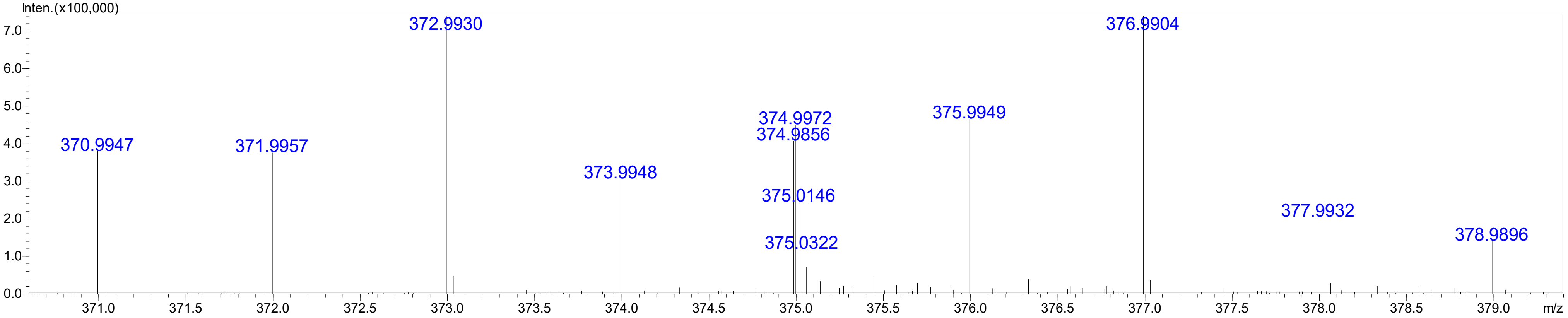

Figure S60. ESI MS spectrum of **15f**.

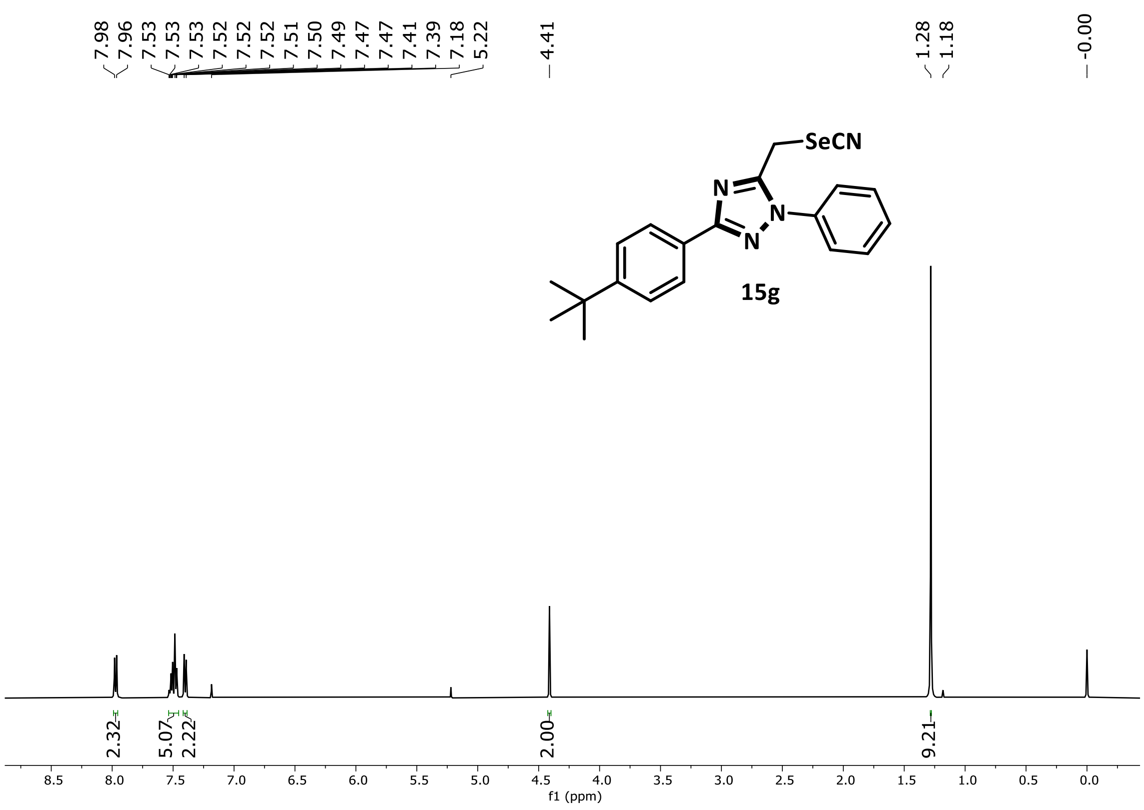

Figure S61. ^{1}H NMR spectrum of compound **15g** in $CDCl_3$ at 500 MHz.

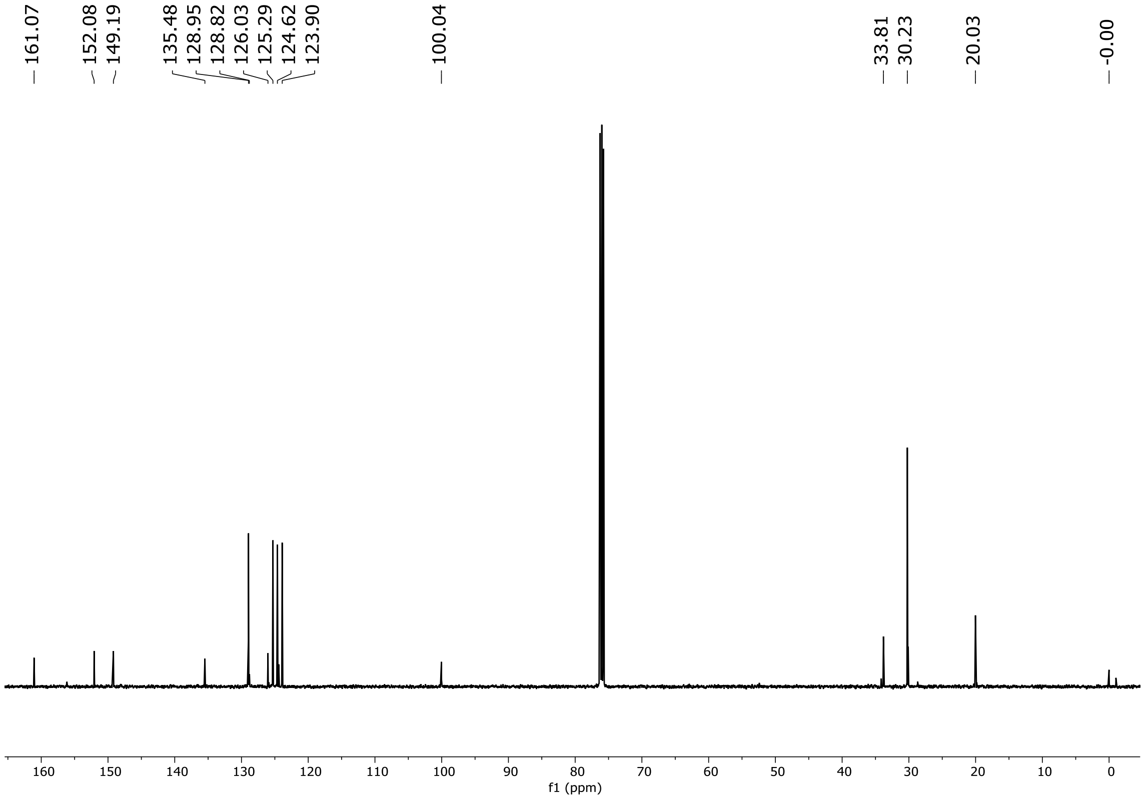

Figure S62. ^{13}C NMR spectrum of compound **15g** in $CDCl_3$ at 125 MHz.

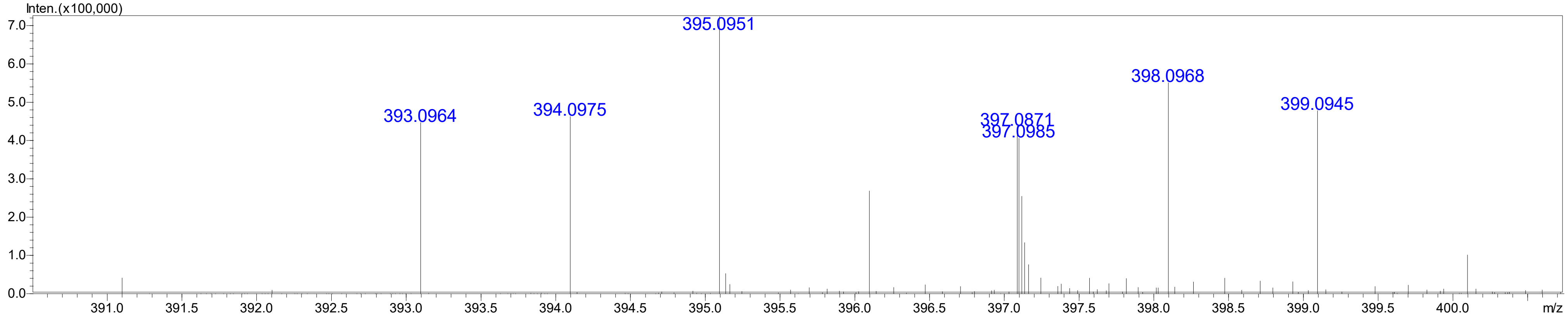

Figure S63. ESI MS spectrum of **15g**.

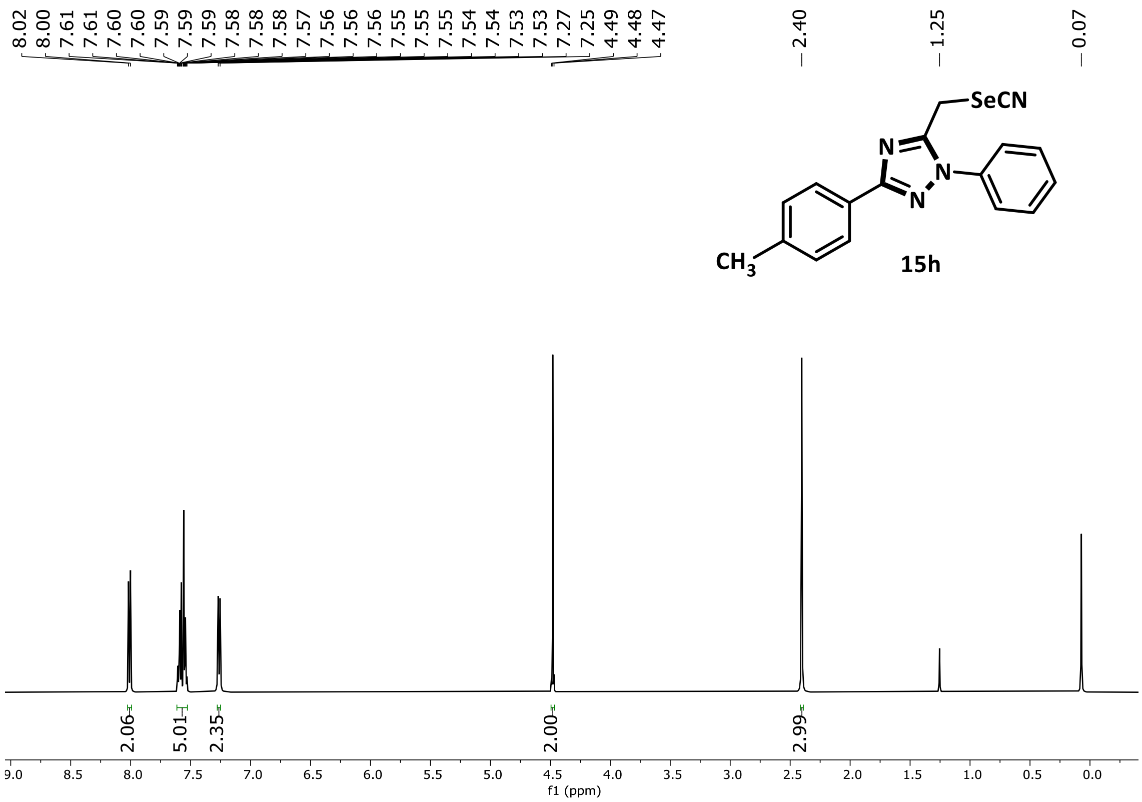

Figure S64. ^{1}H NMR spectrum of compound **15h** in $CDCl_3$ at 500 MHz.

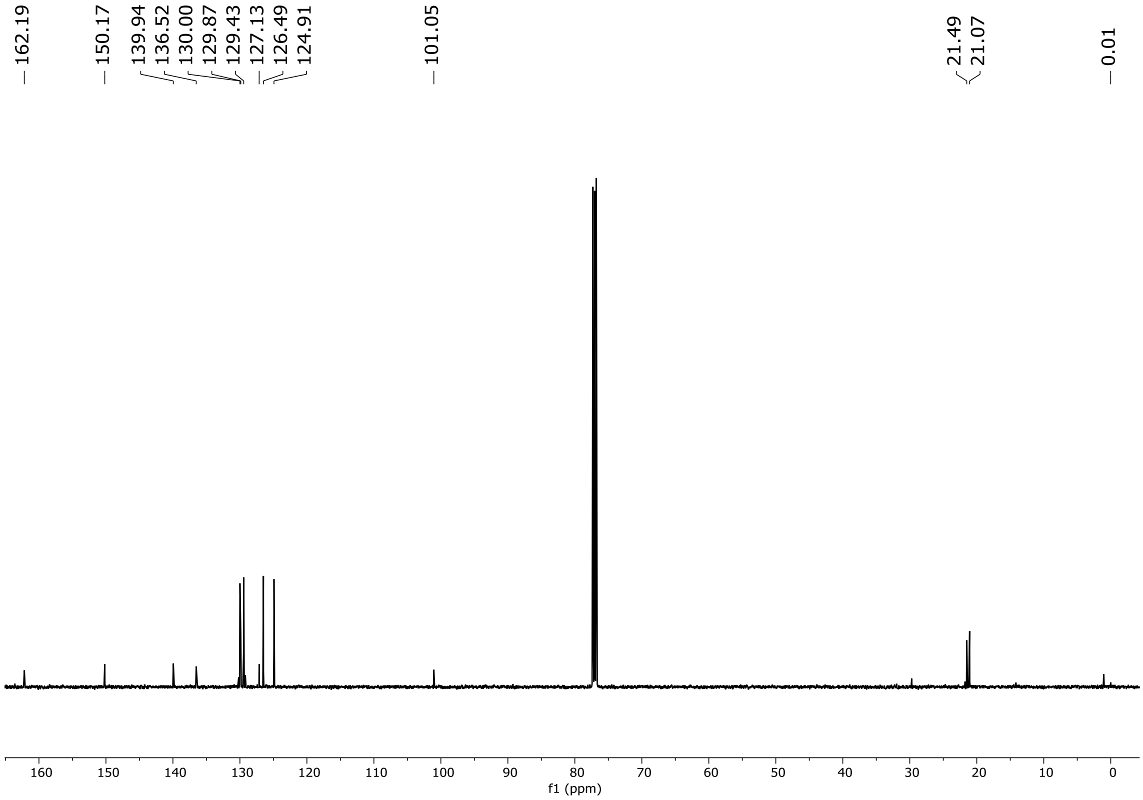

Figure S65. ^{13}C NMR spectrum of compound **15h** in $CDCl_3$ at 125 MHz.

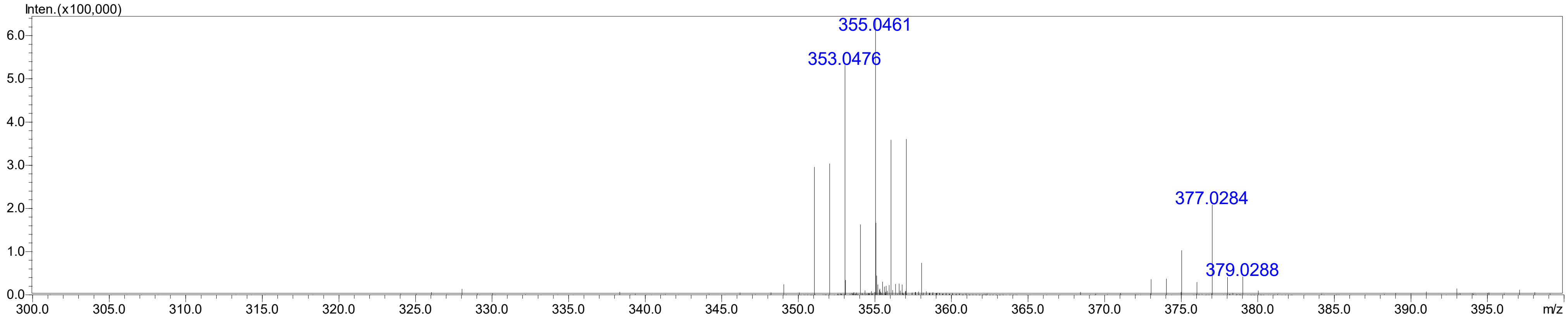

Figure S66. ESI MS spectrum of **15h**.

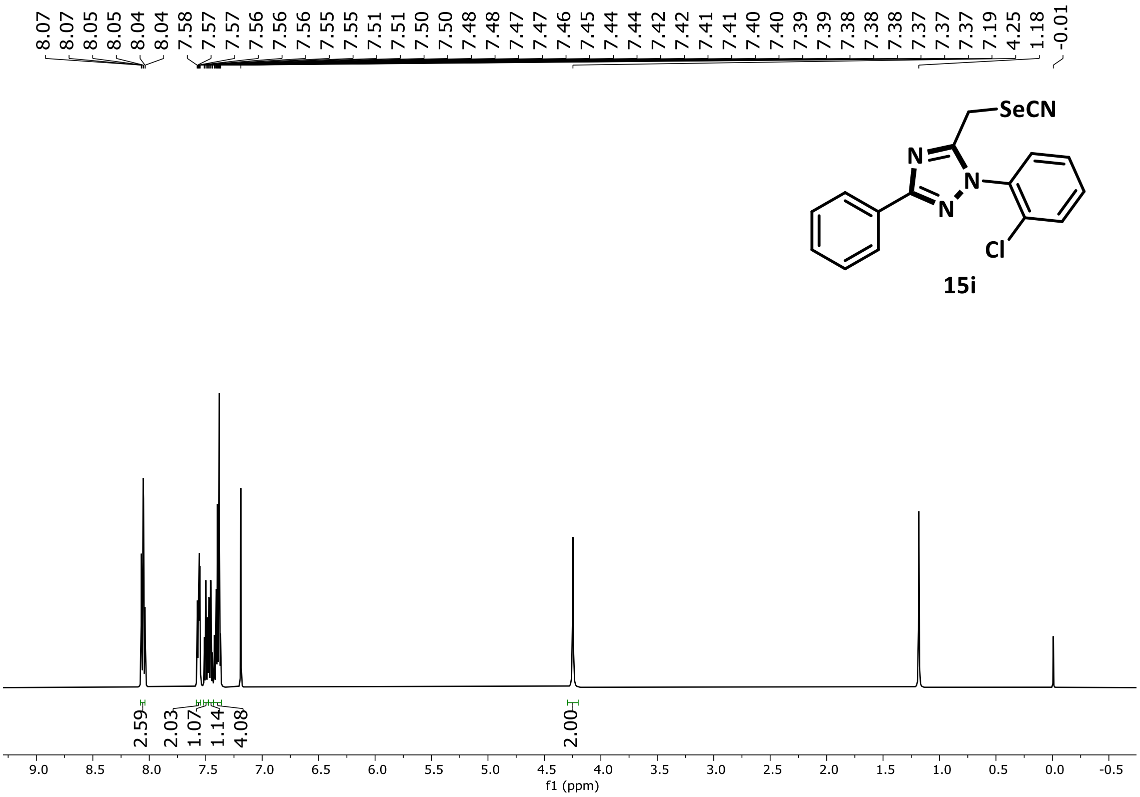

Figure S67. ^{1}H NMR spectrum of compound **15i** in $CDCl_3$ at 500 MHz.

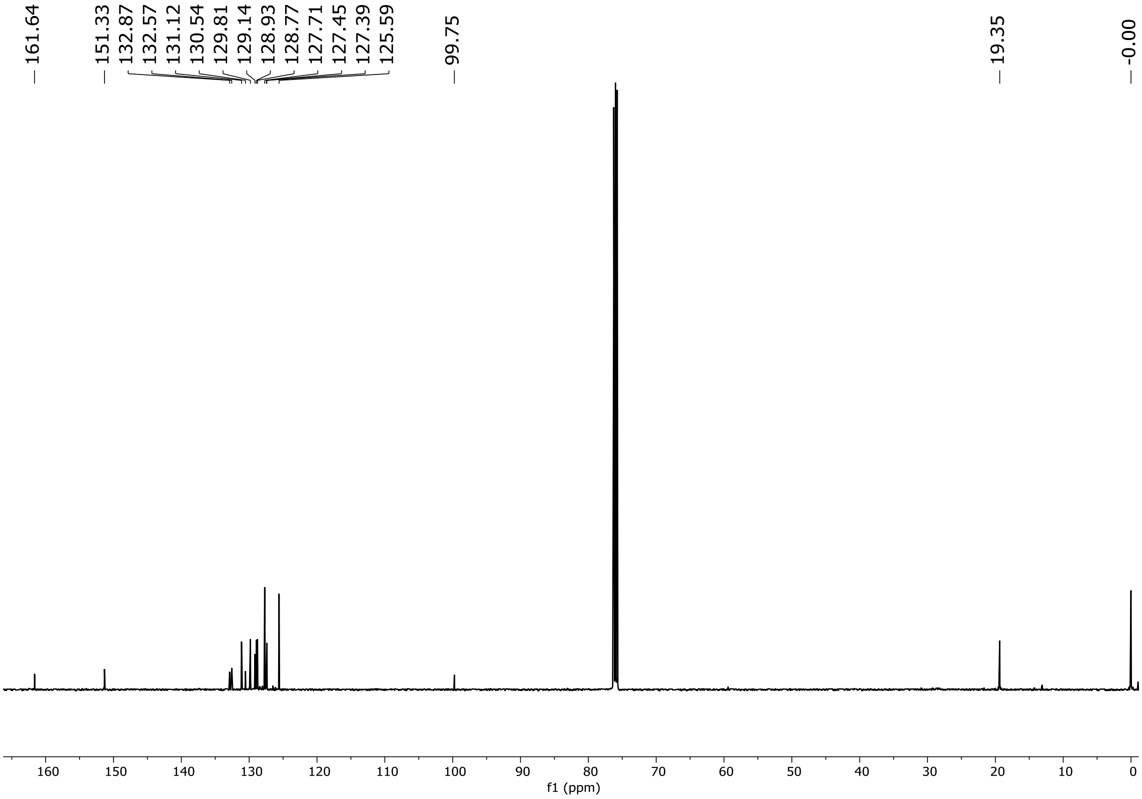

Figure S68. ^{13}C NMR spectrum of compound **15i** in $CDCl_3$ at 125 MHz.

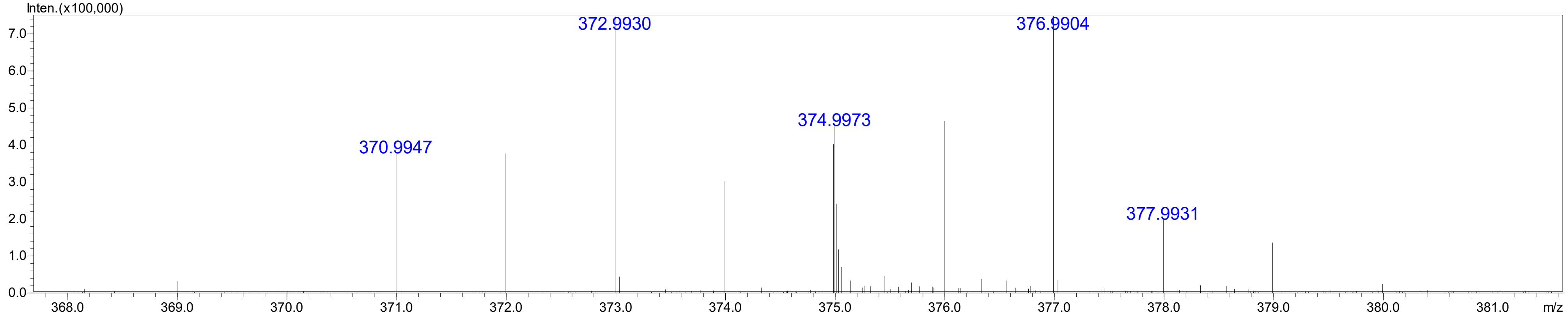

Figure S69. ESI MS spectrum of **15i**.

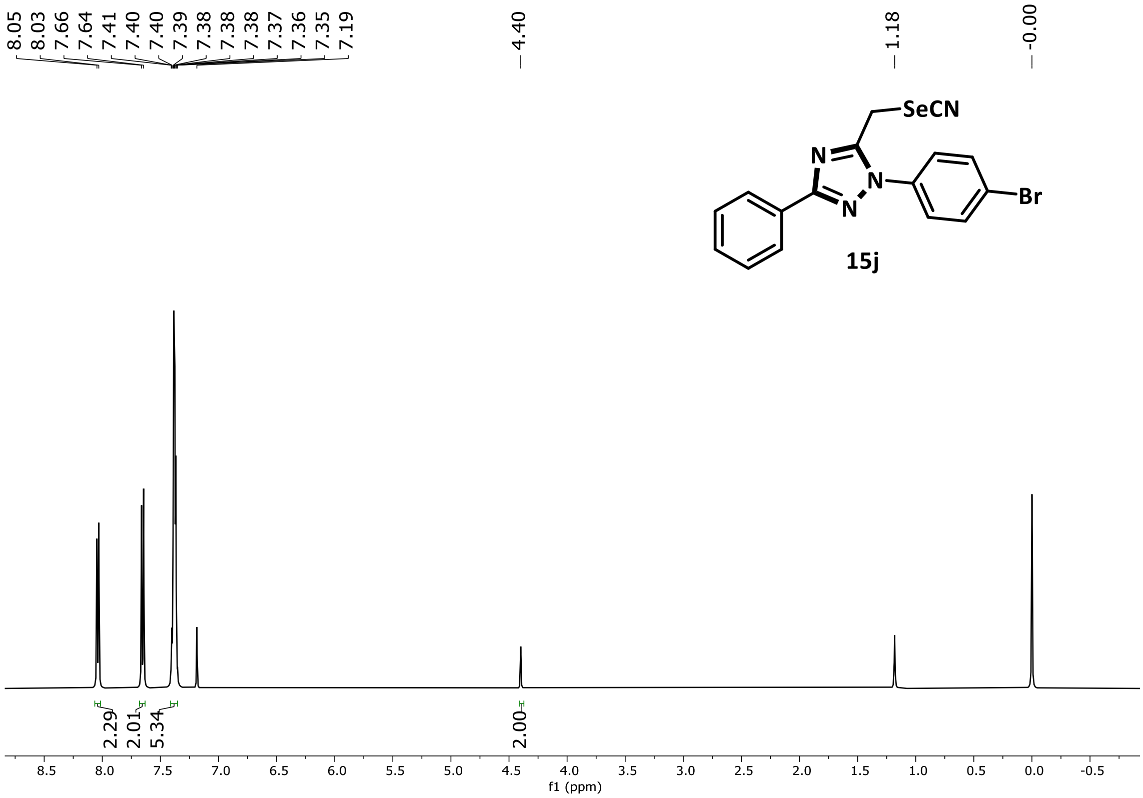

Figure S70. ^{1}H NMR spectrum of compound **15j** in $CDCl_3$ at 500 MHz.

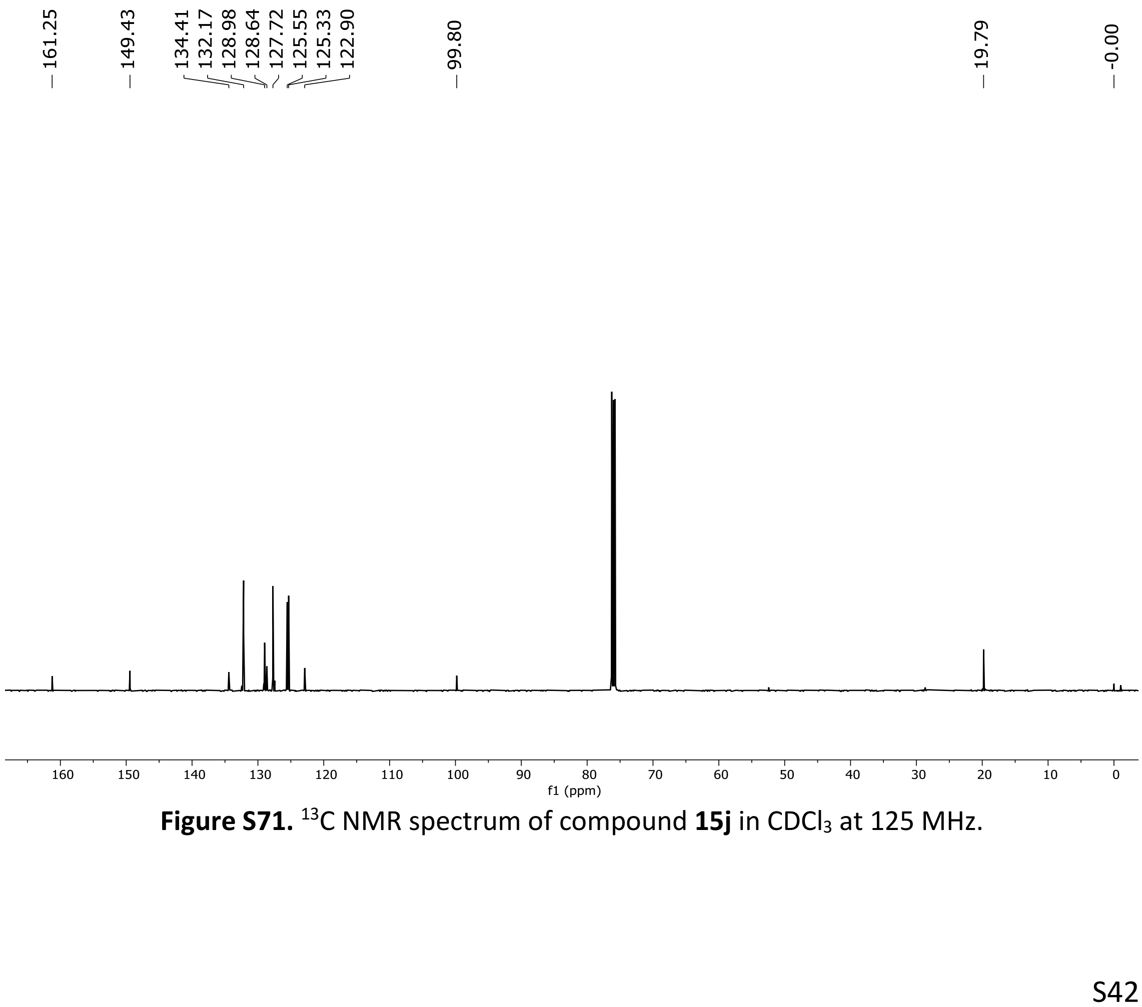

Figure S71. ^{13}C NMR spectrum of compound **15j** in $CDCl_3$ at 125 MHz.

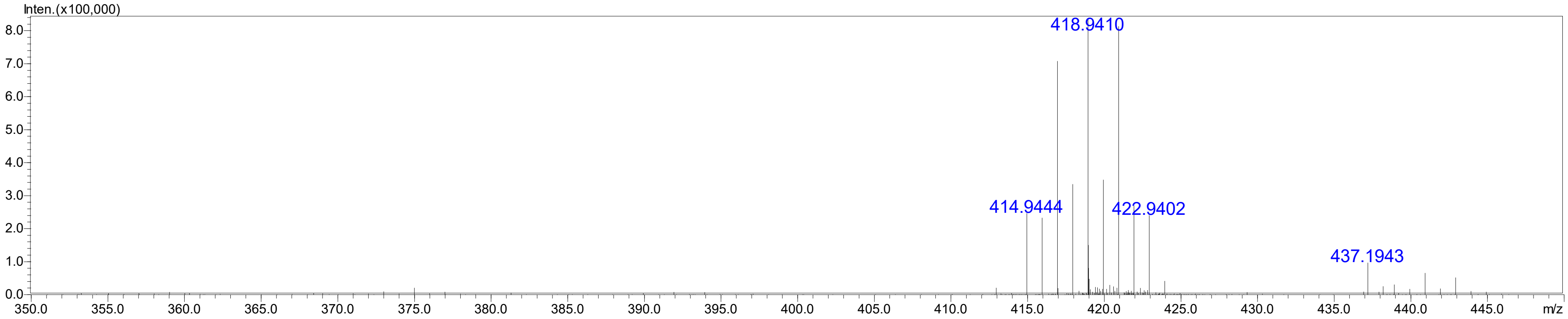

Figure S72. ESI MS spectrum of **15j**.

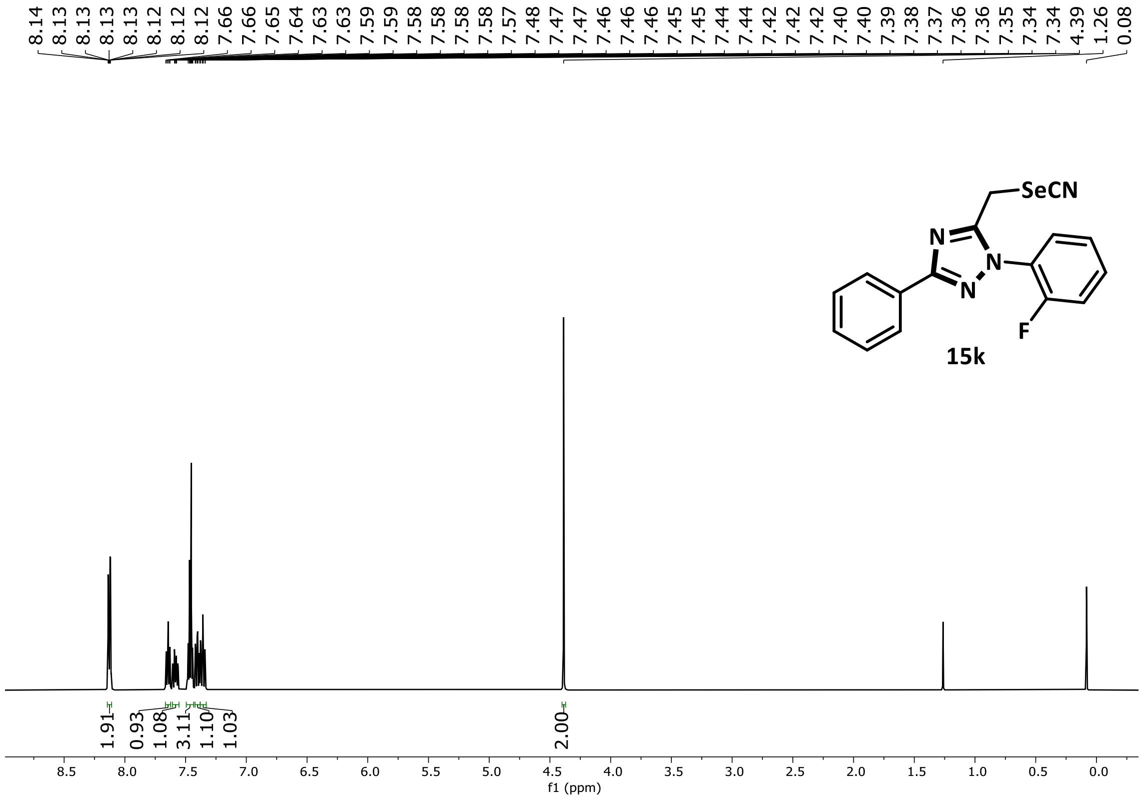

Figure S73. ^{1}H NMR spectrum of compound **15k** in $CDCl_3$ at 500 MHz.

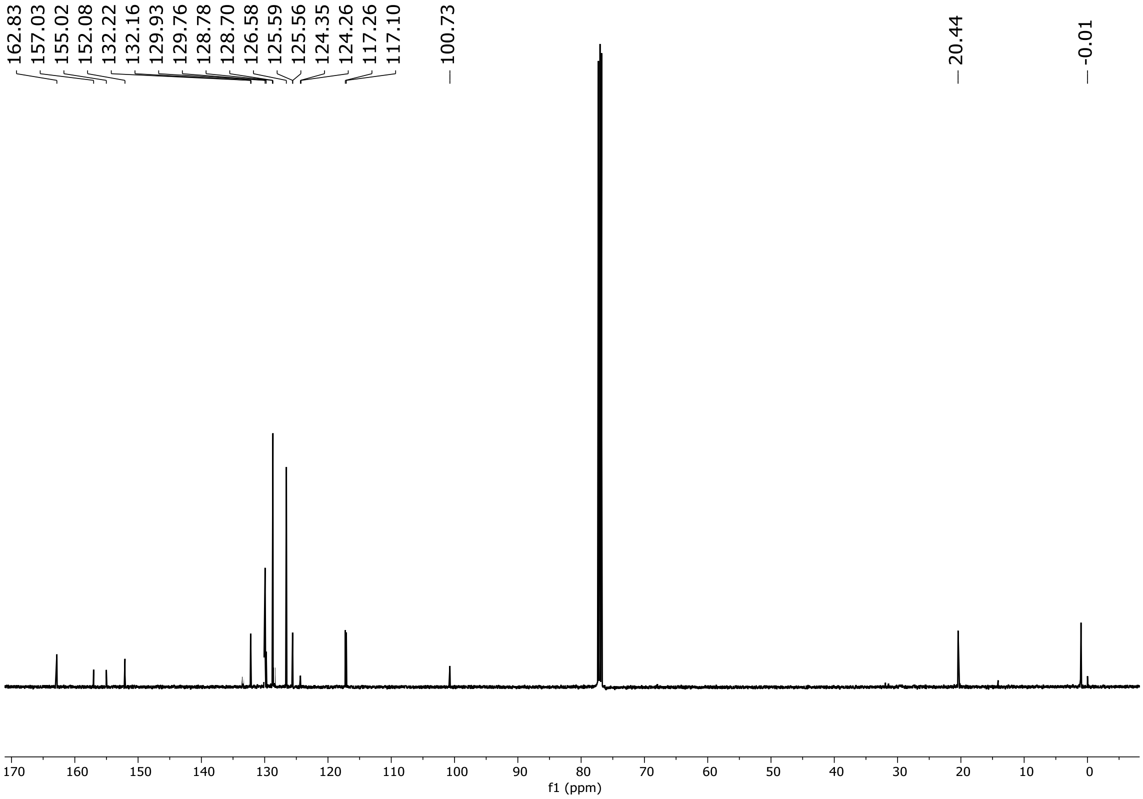

Figure S74. ^{13}C NMR spectrum of compound **15k** in $CDCl_3$ at 125 MHz.

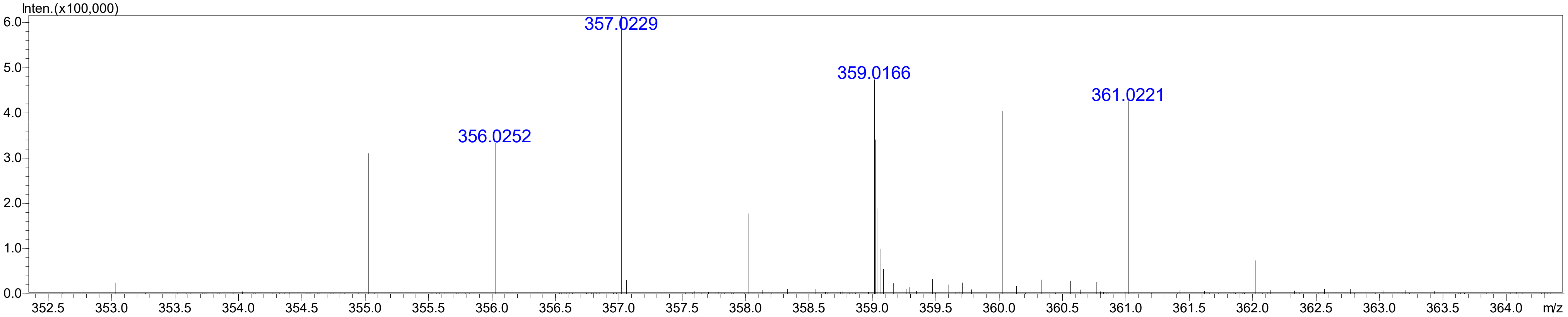

Figure S75. ESI MS spectrum of **15k**.

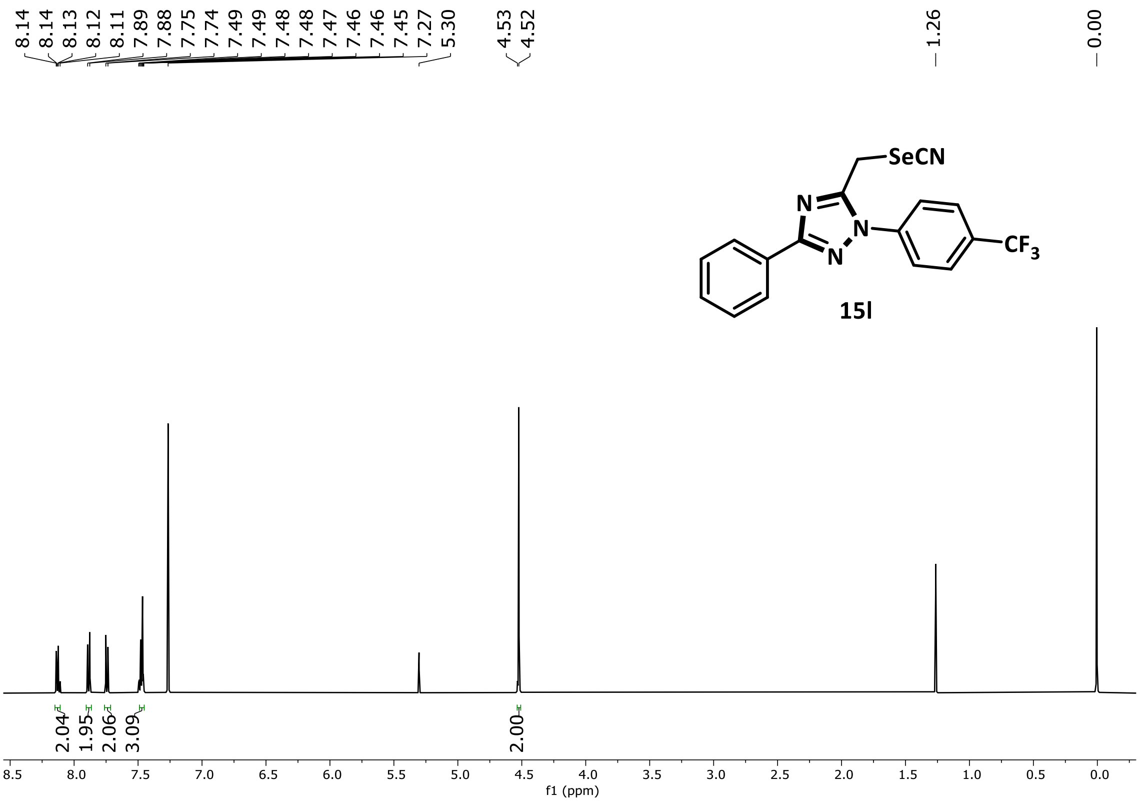

Figure S76. ^{1}H NMR spectrum of compound **15l** in $CDCl_3$ at 500 MHz.

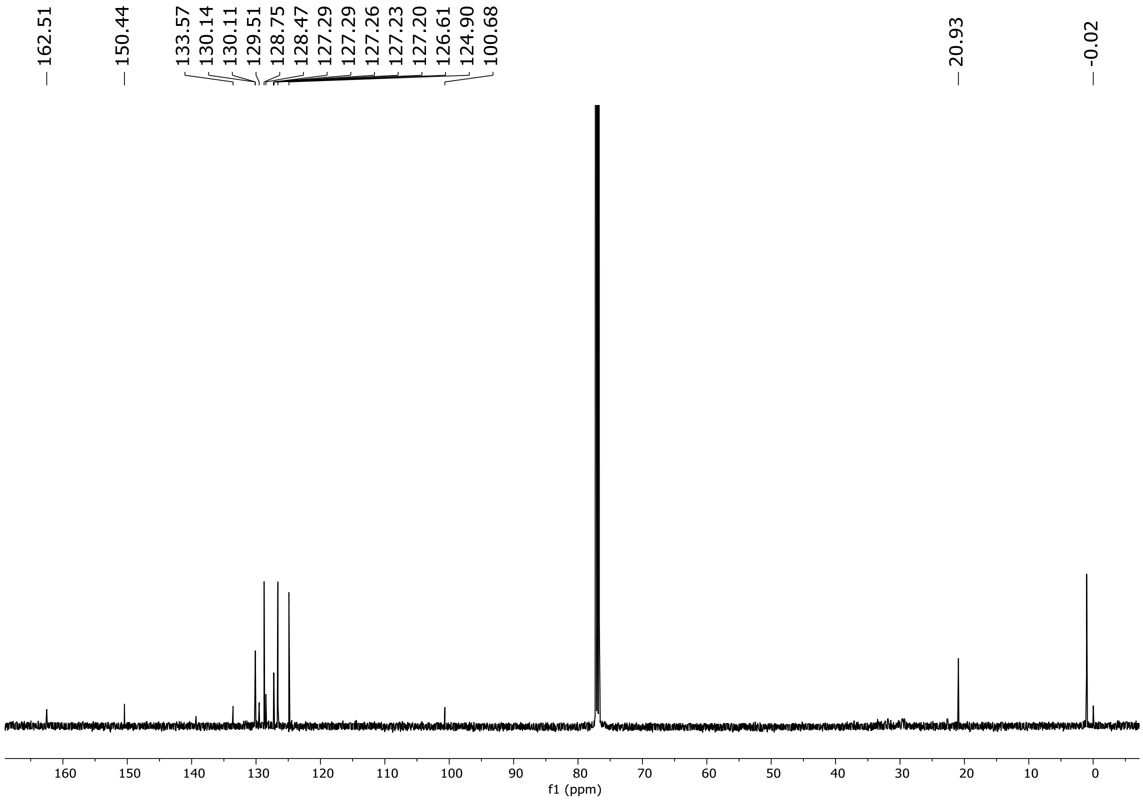

Figure S77. ^{13}C NMR spectrum of compound **15l** in $CDCl_3$ at 125 MHz.

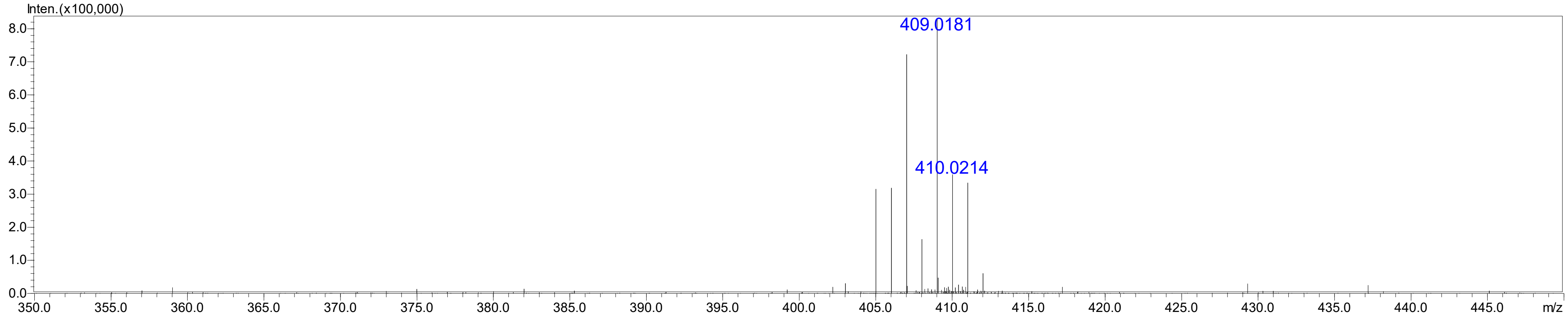

Figure S78. ESI MS spectrum of **15l**.

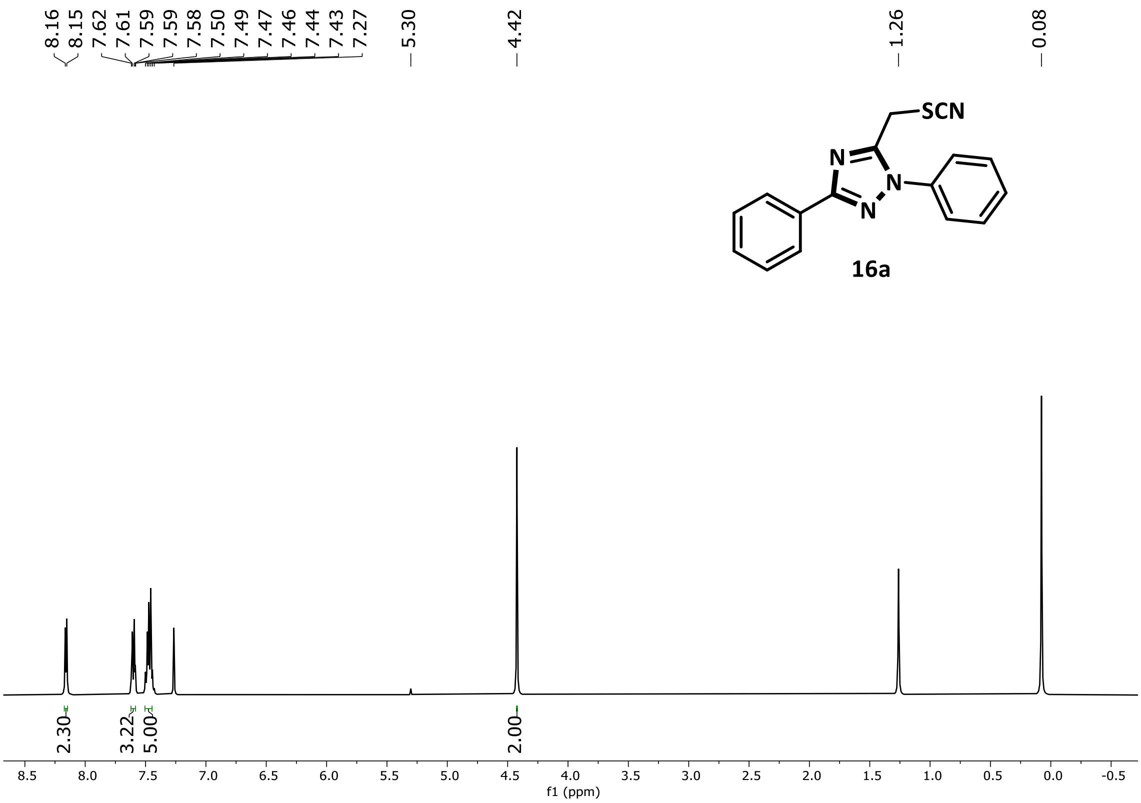

Figure S79. ^{1}H NMR spectrum of compound **16a** in $CDCl_3$ at 500 MHz.

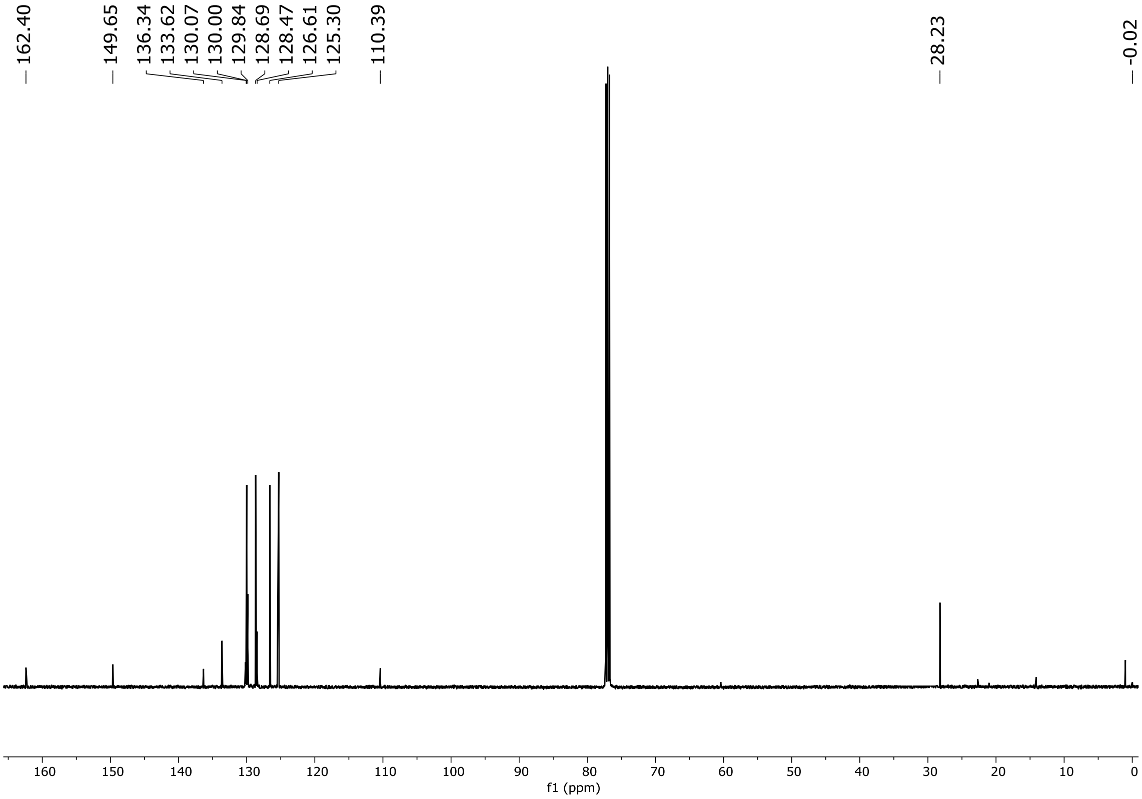

Figure S80. ^{13}C NMR spectrum of compound **16a** in $CDCl_3$ at 125 MHz.

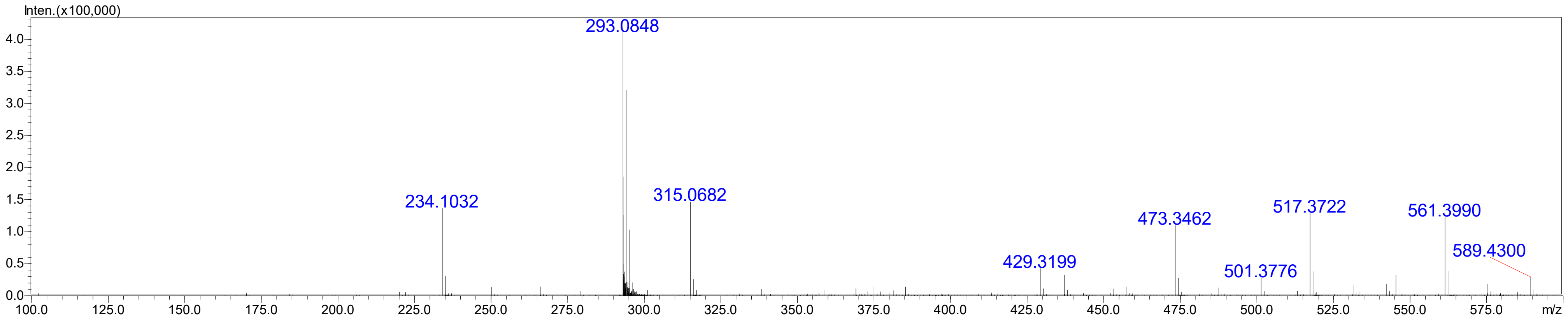

Figure S81. ESI MS spectrum of **16a**.

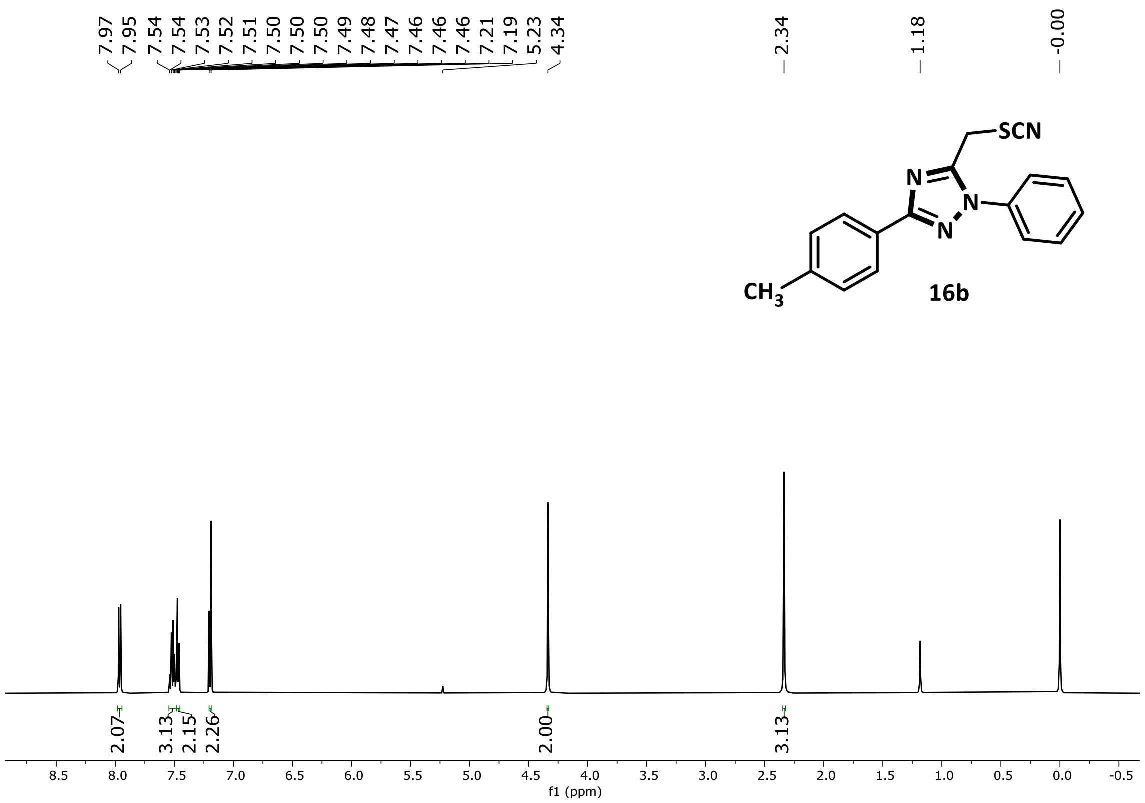

Figure S82. ^{1}H NMR spectrum of compound **16b** in $CDCl_3$ at 500 MHz.

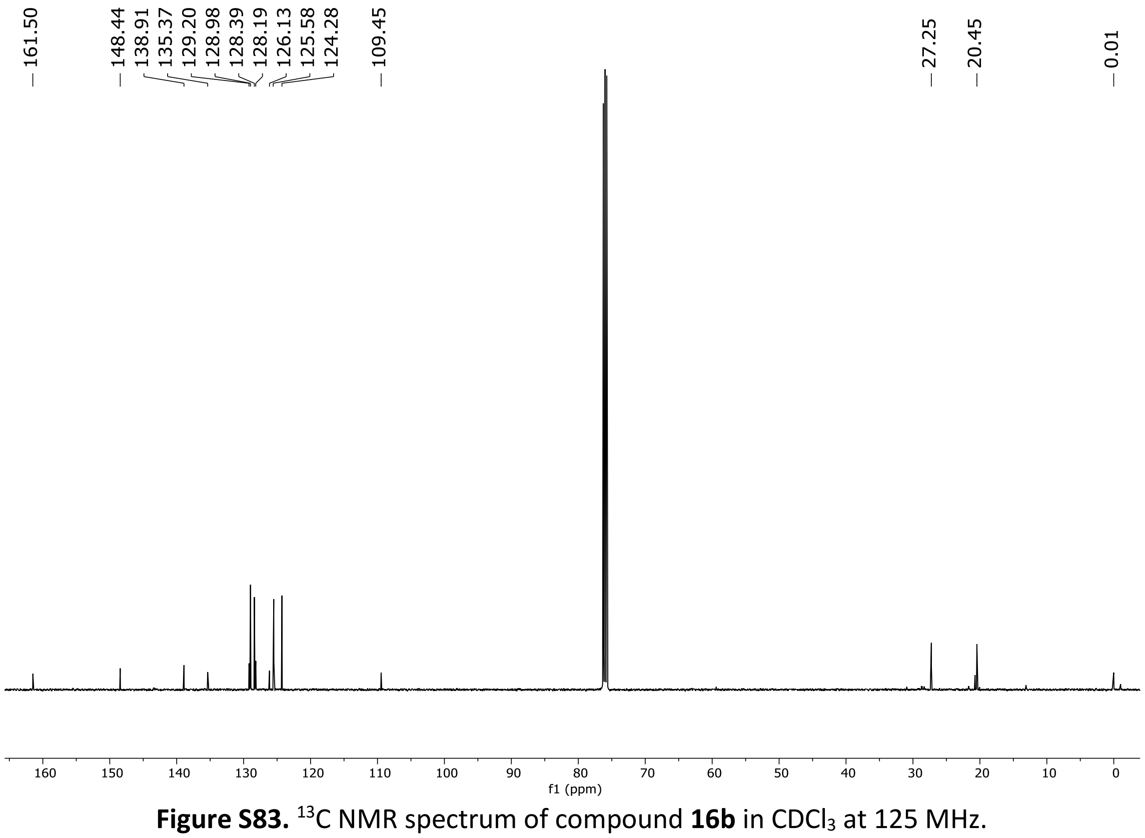

Figure S83. ^{13}C NMR spectrum of compound **16b** in $CDCl_3$ at 125 MHz.

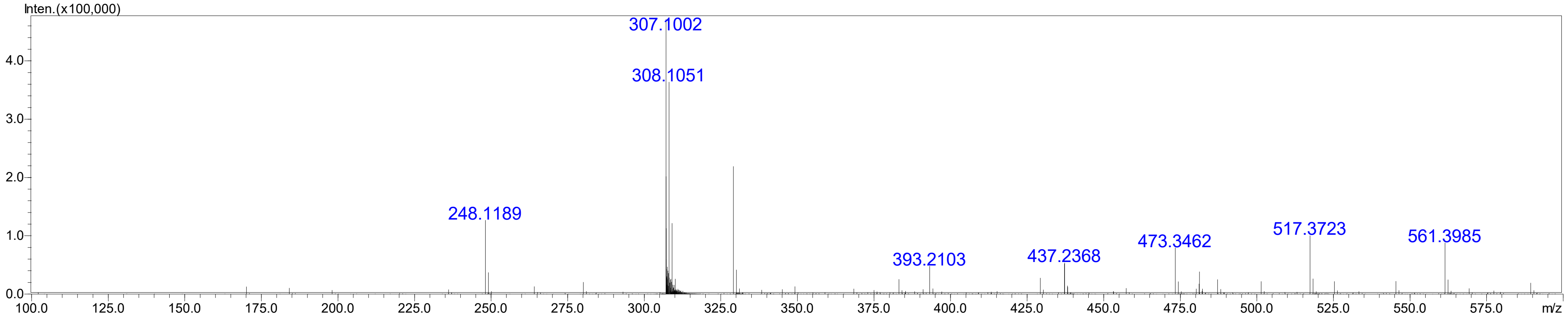

Figure S84. ESI MS spectrum of **16b**.

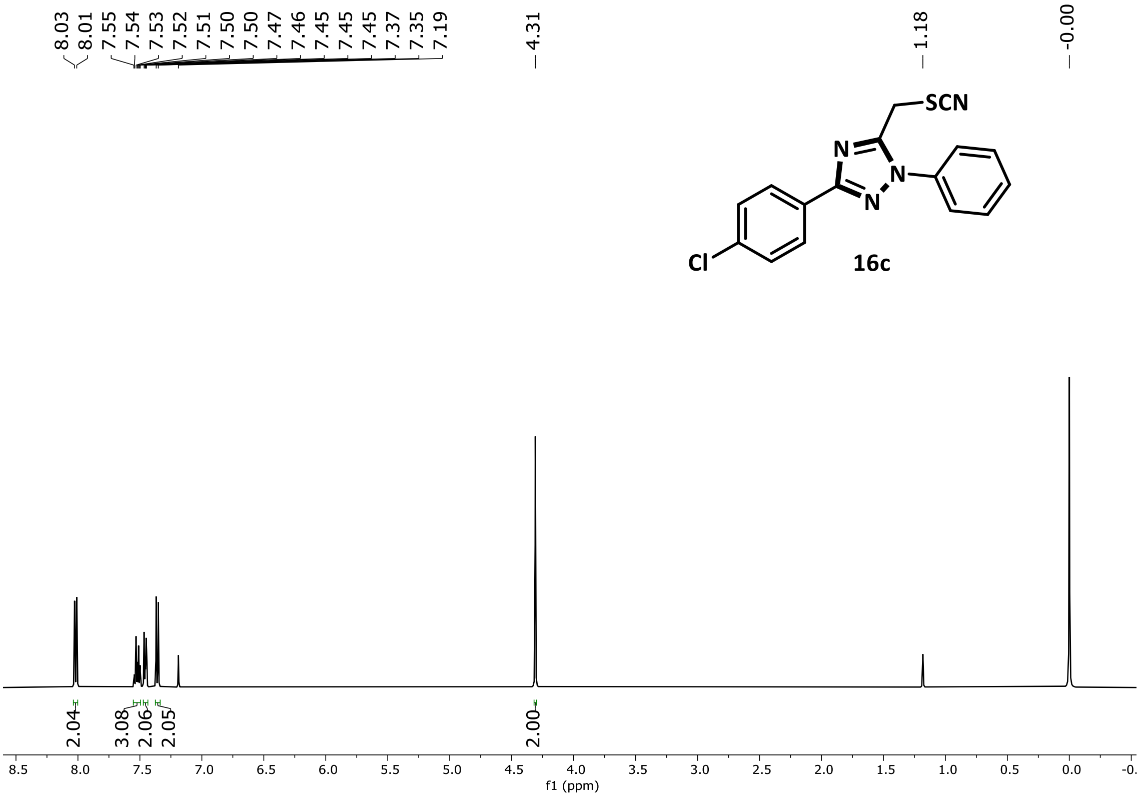

Figure S85. ^{1}H NMR spectrum of compound **16c** in $CDCl_3$ at 500 MHz.

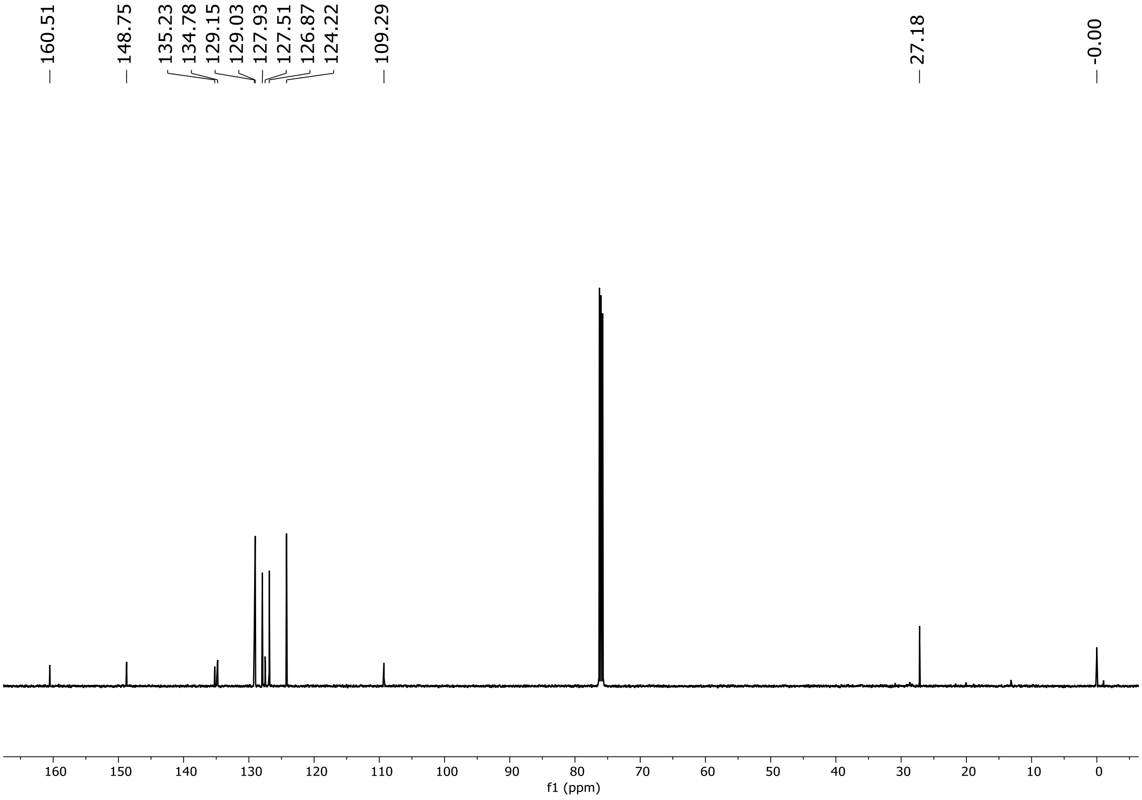

Figure S86. ^{13}C NMR spectrum of compound **16c** in $CDCl_3$ at 125 MHz.

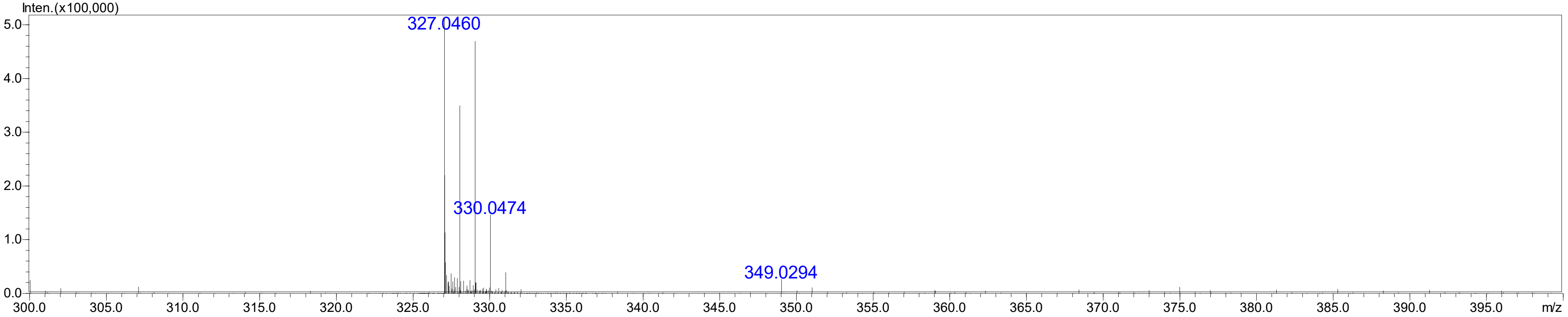

Figure S87. ESI MS spectrum of **16c**.

S2. *In silico* pharmacokinetic analysis

Additional in silico pharmacokinetic and drug-likeness data for the selected chalcogen–triazole derivatives were generated using SwissADME and ADMETlab 3.0. The full set of predicted physicochemical descriptors, absorption, distribution, metabolism, excretion, and toxicity-related parameters is provided in **Table S2**. These data support the qualitative comparisons discussed in the main text and further illustrate the consistency between experimental GPx-like activity and predicted developability profiles.

Table S2. Summary of in silico ADMET and drug-likeness properties of the selected chalcogen–triazole derivatives, calculated using SwissADME and ADMETlab 3.0.

Compound	Chalcogen	Molecular weight	TPSA (Å²)	Consensus LogP	GI absorption	BBB permeation	P-gp substrate	Predicted CYP inhibition*	PAINS alerts	Major ADMET flags
**15a**	**Se**	Moderate	~54.5	Moderate	High	Yes	Yes	CYP1A2, CYP2C19, CYP3A4	None	None
**15h**	**Se**	Moderate	~54.5	Moderate	High	Yes	Yes	CYP2C19, CYP2C9, CYP3A4	None	None
**15i**	**Se**	Moderate–high	~54.5	Moderate–high	High	Yes	Yes	CYP1A2, CYP2C19, CYP3A4	None	None
**15j**	**Se**	Moderate	~54.5	Moderate	High	Yes	Yes	CYP1A2, CYP2C19, CYP3A4	None	None
**15l**	**Se**	High	~54.5	High	High	Yes	Yes	CYP2C19, CYP3A4	None	None
**16a**	**S**	Moderate	~79.8	Moderate–high	High	No	No	CYP1A2, CYP2C19, CYP3A4	None	None
**16c**	**S**	Moderate	~79.8	Moderate	High	No	No	CYP2C19, CYP3A4	None	None

* CYP inhibition indicates predicted interaction with one or more cytochrome P450 isoforms.
All predictions are qualitative and intended for comparative analysis only.
MW: molecular weight; TPSA: topological polar surface area; GI: gastrointestinal; BBB: blood–brain barrier; P-gp: P-glycoprotein.

S3. Computational details

Density functional theory (DFT) calculations were performed to investigate the electronic structure and redox-related properties of the selected chalcogen–triazole derivatives. All calculations were carried out using a standard quantum chemical protocol commonly applied to organochalcogen compounds.

Initial molecular geometries were built based on experimentally characterised structures and subsequently fully optimised without symmetry constraints using the DFT formalism. Geometry optimisations were followed by frequency calculations at the same level of theory to confirm that all optimised structures correspond to true local minima on the potential energy surface, as evidenced by the absence of imaginary frequencies.

Frontier molecular orbital energies, including the highest occupied molecular orbital (HOMO) and the lowest unoccupied molecular orbital (LUMO), were extracted from the converged wavefunctions. HOMO–LUMO energy gaps (ΔE_gap) were calculated directly as the energy difference between the corresponding orbitals. Molecular orbital isosurfaces were generated using a constant isovalue of 0.02 a.u. to allow consistent visual comparison of orbital localisation and delocalisation patterns across the compound series.

All quantum chemical calculations were performed using density functional theory with an exchange–correlation functional and basis set appropriate for systems containing second- and third-row elements, including sulfur and selenium. When applicable, effective core potentials were employed for heavy atoms to account for relativistic effects. Solvent effects were considered using an implicit solvation model representative of the experimental conditions, where relevant.

The analysis focused on qualitative interpretation of frontier orbital energies and spatial distributions, with the aim of rationalising experimentally observed trends in GPx-like catalytic activity rather than providing quantitative reactivity predictions. The resulting electronic descriptors were therefore interpreted in conjunction with experimental redox data and in silico pharmacokinetic profiles.

S4. Frontier molecular orbital analysis

The complete set of HOMO and LUMO isosurfaces for the selected chalcogen–triazole derivatives (**15a**, **15h**, **15i**, **15j**, **15l**, **16a**, and **16c**) is shown in Figures S69 and S70. These representations complement the representative examples discussed in the main text and allow direct visual comparison of orbital localisation patterns across selenium- and sulfur-containing analogues.

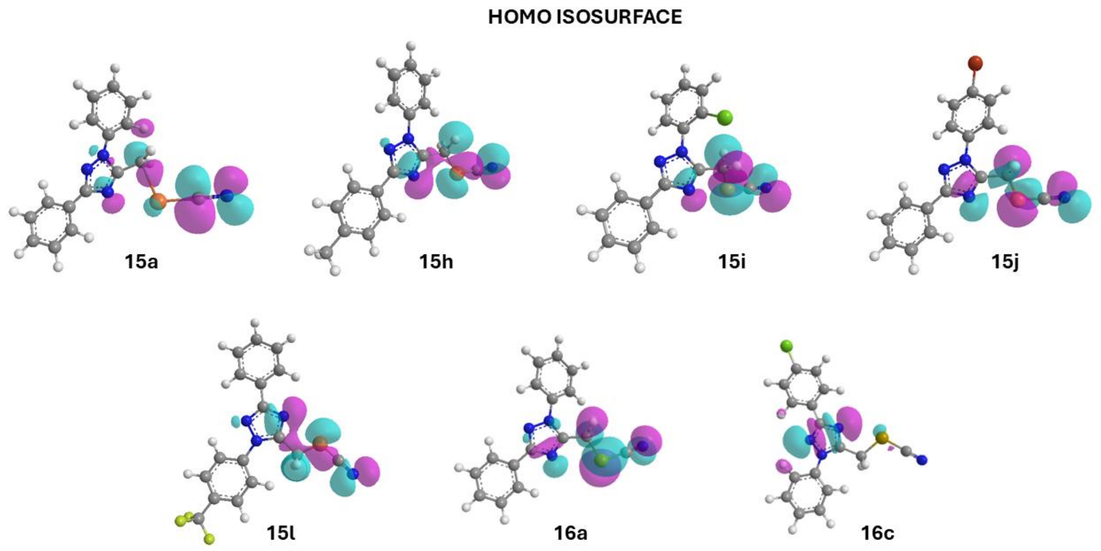

Figure S88. HOMO isosurfaces of the selected chalcogen–triazole derivatives (**15a**, **15h**, **15i**, **15j**, **15l**, **16a**, and **16c**) obtained from DFT calculations. The isosurfaces illustrate differences in orbital localisation associated with chalcogen identity and substitution pattern.

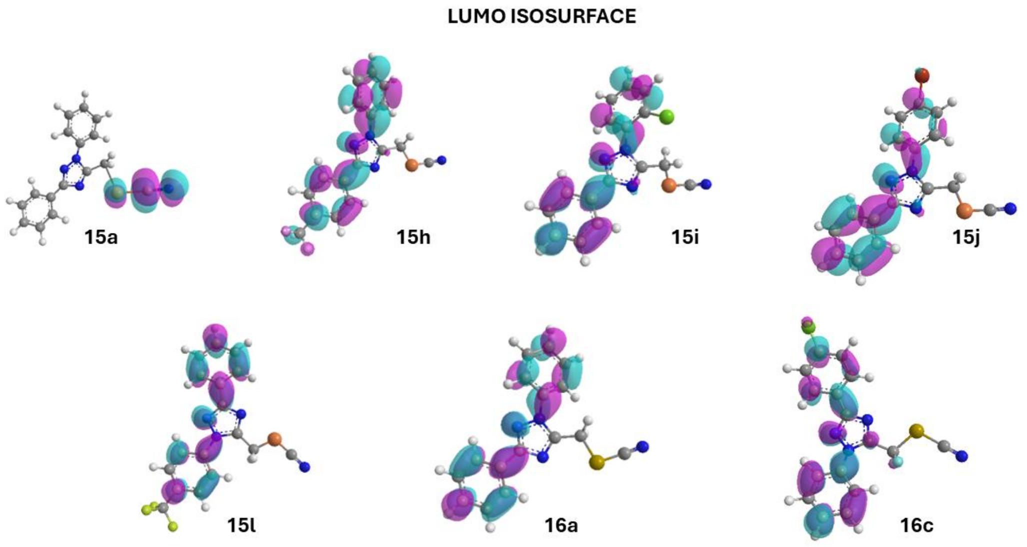

Figure S89. LUMO isosurfaces of the selected chalcogen–triazole derivatives (**15a**, **15h**, **15i**, **15j**, **15l**, **16a**, and **16c**) obtained from DFT calculations, highlighting variations in orbital delocalisation relevant to redox behaviour.
